# Human mitochondrial transcriptional factor A breaks the mitochondria-mediated vicious cycle in Alzheimer’s disease

**DOI:** 10.1038/srep37889

**Published:** 2016-11-29

**Authors:** Sugako Oka, Julio Leon, Kunihiko Sakumi, Tomomi Ide, Dongchon Kang, Frank M. LaFerla, Yusaku Nakabeppu

**Affiliations:** 1 Division of Neurofunctional Genomics, Department of Immunobiology and Neuroscience, Medical Institute of Bioregulation, Kyushu University, 3-1-1 Maidashi, Higashi-Ku, Fukuoka 812-8582, Japan; 2 Department of Cardiovascular Medicine, Graduate School of Medical Sciences, Kyushu University, 3-1-1 Maidashi, Higashi-Ku, Fukuoka 812-8582, Japan; 3 Department of Clinical Chemistry and Laboratory Medicine, Graduate School of Medical Sciences, Kyushu University, 3-1-1 Maidashi, Higashi-Ku, Fukuoka 812-8582, Japan; 4 Department of Neurobiology and Behavior, University of California, Irvine, CA 92697, USA

## Abstract

In the mitochondria-mediated vicious cycle of Alzheimer’s disease (AD), intracellular amyloid β (Aβ) induces mitochondrial dysfunction and reactive oxygen species, which further accelerate Aβ accumulation. This vicious cycle is thought to play a pivotal role in the development of AD, although the molecular mechanism remains unclear. Here, we examined the effects of human mitochondrial transcriptional factor A (hTFAM) on the pathology of a mouse model of AD (3xTg-AD), because TFAM is known to protect mitochondria from oxidative stress through maintenance of mitochondrial DNA (mtDNA). Expression of hTFAM significantly improved cognitive function, reducing accumulation of both 8-oxoguanine, an oxidized form of guanine, in mtDNA and intracellular Aβ in 3xTg-AD mice and increasing expression of transthyretin, known to inhibit Aβ aggregation. Next, we found that AD model neurons derived from human induced pluripotent stem cells carrying a mutant *PSEN1*_(P117L)_ gene, exhibited mitochondrial dysfunction, accumulation of 8-oxoguanine and single-strand breaks in mtDNA, and impaired neuritogenesis with a decreased expression of transthyretin, which is known to be downregulated by oxidative stress. Extracellular treatment with recombinant hTFAM effectively suppressed these deleterious outcomes. Moreover, the treatment increased expression of transthyretin, accompanied by reduction of intracellular Aβ. These results provide new insights into potential novel therapeutic targets.

Mitochondria are the principal mediators of cellular life and death through their ATP production, metabolic fatty acid oxidation, glucose metabolism, calcium or iron handling, and apoptosis regulation. Mitochondria, however, also generate reactive oxygen species (ROS) as byproducts of oxidative phosphorylation, and this generation increases during aging. Thus, mitochondrial DNA (mtDNA) is more susceptible than nuclear DNA to oxidative stress^1^.

Among the various types of oxidative damage, 8-oxoguanine (8-oxoG) is a major oxidation product in DNA/RNA and nucleotide pools^2^. Previously, we demonstrated that accumulation of 8-oxoG in mtDNA induces mitochondrial dysfunction, resulting in cell death^3^, and that mutant mice lacking both *Mth1* and *Ogg1* genes—the former encoding 8-oxo-dGTPase and the latter encoding 8-oxoG DNA glycosylase—accumulate 8-oxoG in neuronal mtDNA, causing neurodegeneration accompanied by mitochondrial dysfunction under oxidative conditions^4^. These results suggest that protecting mtDNA from oxidative stress is crucial for maintaining normal neuronal function.

Mitochondrial dysfunction reportedly plays a pivotal role in age-related neurodegenerative disorders, such as Alzheimer’s disease (AD)^5^,^6^. Accumulation of 8-oxoG in mtDNA and reduced mitochondrial function have been observed in the AD brain^7^,^8^. We also reported that gene expression of *NEUROD6*, a neurogenic basic helix-loop-helix transcription factor that triggers an antioxidant response and sustains mitochondrial biomass, is downregulated in the AD brain^6^. Mitochondrially transformed cells (cybrids) created from AD patients exhibit decreased complex IV activity and ROS generation^9^. The triple-transgenic mouse model of AD (3xTg-AD) shows decreased mitochondrial respiration with reduced levels of pyruvate dehydrogenase protein and activity as early as 3 months of age^10^, suggesting that mitochondrial dysfunction is important in early stages of AD.

Intracellular accumulation of amyloid β (Aβ) is thought to be the primary event in the pathogenesis of AD. Accumulation of Aβ in the cytoplasm induces mitochondrial dysfunction and ROS by interacting with the mitochondrial matrix component Aβ-binding alcohol dehydrogenase^11^. The increased ROS generation in aged mitochondria accelerates Aβ accumulation^12^. This mitochondria-mediated vicious cycle of oxidative stress and intracellular Aβ accumulation plays a pivotal role in the development of AD. However, the molecular mechanism for this vicious cycle remains unclear.

The mitochondrial transcriptional factor A (TFAM) plays an essential role in the maintenance of mitochondrial homeostasis by regulating its transcription and maintaining mtDNA^13^. TFAM binds mtDNA and forms a nucleoid-like structure, thus protecting mtDNA from DNA damage caused by ROS^13^,^14^,^15^,^16^. In late-onset AD, two TFAM polymorphisms have been reported in Caucasian and northern Han Chinese populations^17^,^18^. Moreover, our group previously reported that transgenic expression of human TFAM (hTFAM) ameliorates age-dependent memory impairment and decreases 8-oxoG accumulation in aged mice^19^, suggesting that increased expression of hTFAM may suppress mitochondrial dysfunction in a mouse model of AD, thereby ameliorating AD pathology and cognitive impairments.

In the present study, to clarify the mechanism of the mitochondria-mediated vicious cycle in AD, we examined the effects of hTFAM on the pathophysiology of 3xTg-AD mice and in a cell culture model of AD using human neurons derived from induced pluripotent stem cells (iPSCs).

## Results

### Establishment of triple-transgenic AD model mice expressing human TFAM protein

To investigate whether mitochondrial dysfunction is crucial for the development of AD, we introduced an hTFAM transgene^19^,^20^ into 3xTg-AD mice, which carry the *APP*_*Swe*_ and *MAPT*_*P301L*_ transgenes with a *Psen1*_M146V_ knock-in mutation^21^. The 3xTg-AD mice develop an age-related and progressive neuropathological phenotype, which includes Aβ plaques, neurofibrillary tangles, and synaptic dysfunction^21^, accompanied by mitochondrial dysfunction^22^. We thus established three lines of mice, non-transgenic control mice (Non-Tg), hemizygous 3xTg-AD mice without the hTFAM transgene (ADh/WT), and hemizygous 3xTg-AD mice with the hemizygous hTFAM transgene (ADh/hTFAMh). Expression of hTFAM in the brain was examined by western blot analysis (Supplementary Fig. S1a). An increase in the level of a 24-kDa polypeptide was detected in cortex extracts prepared from ADh/hTFAMh mice. Immunofluorescence microscopy revealed increased immunoreactivity for hTFAM in the ADh/hTFAMh brain, particularly in NeuN-positive neurons, compared with that in the ADh/WT brain (Supplementary Fig. S1b). In neurons, hTFAM immunoreactivity was mainly detected in cell body and some neurites (Supplementary Fig. S1b, bottom).

**Figure 1:**
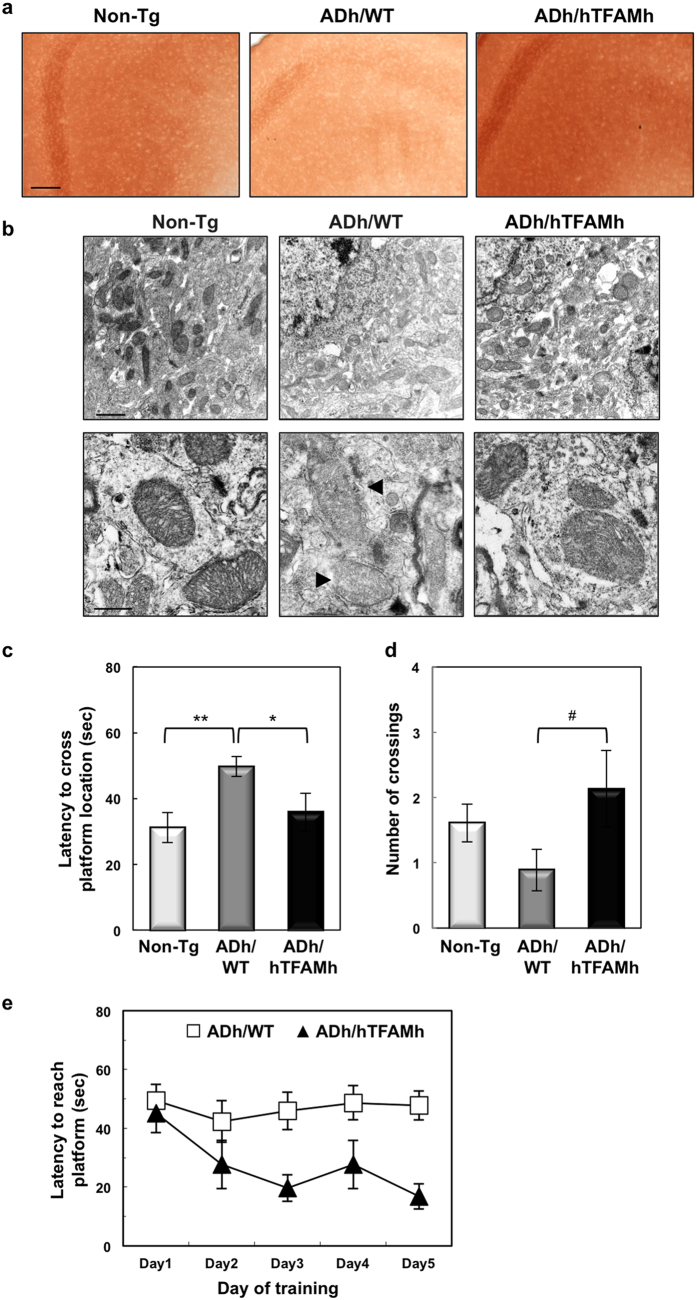
Expression of hTFAM improved mitochondrial function in the ADh/WT cerebral cortex and cognitive impairment in ADh/WT mice. (**a**) Expression of hTFAM improved mitochondrial function in the ADh/WT cerebral cortex. Mitochondrial function was determined by enzymatic staining for cytochrome c oxidase (COX). Frozen sections from the cortex of 13–14-month-old Non-Tg, ADh/WT, and ADh/hTFAMh mice were stained for COX (reddish brown). Scale bar, 20 μm. (**b**) COX enzymatic staining in each mitochondrion in the cortex was visualized by electron microscopy. Arrowheads show degenerating mitochondria characterized by diminished COX staining and disrupted membranes and cristae in the ADh/WT cortex. Scale bars, 1 μm (upper panels); 500 nm (lower panels). (**c**) Latency to cross over the previously hidden platform 24 h after the training trial in the d2 protocol of the Morris water maze using 11–12-month-old Non-Tg, ADh/WT, and ADh/hTFAMh mice. One-way ANOVA, *p* = 0.0076. Tukey’s HSD test (vs. ADh/WT), *p < 0.05, **p < 0.01. Data represent means ± SEM, n = 5–8. (**d**) The number of crossings over the previously hidden platform 24 h after the training trial in the d2 protocol of the Morris water maze using 11–12-month-old Non-Tg, ADh/WT, and ADh/hTFAMh mice. Student’s *t*-test (vs. ADh/WT), ^#^*p* = 0.0463. Data represent means ± SEM, n = 5–8. (**e**) Latency to reach the platform in the d5 protocol of the Morris water maze using 11–12-month-old ADh/WT and ADh/hTFAMh mice. Multivariate analysis of variance (MANOVA), genotype, *p* = 0.0011. Data represent means ± SEM, n = 5–8.

Mitochondrial function was determined by enzymatic staining for cytochrome c oxidase (COX). Histochemical detection of COX activity revealed that 13-month-old ADh/WT mice exhibited decreased COX activity in the cortex compared with Non-Tg mice (Fig. 1a). Electron microscopy results showed that most mitochondria detected in the cortex of Non-Tg mice were densely stained, but mitochondria in the cortex of ADh/WT mice were weakly stained (Fig. 1b, upper panels). As evident in a magnified image (Fig. 1b, lower panels, arrowheads), many degenerating mitochondria with characteristic disrupted membranes and cristae were found in the cortex of ADh/WT mice. In ADh/hTFAMh mice, we found that COX activity was largely restored and that most mitochondria were morphologically normal, indicating that hTFAM efficiently suppresses mitochondrial dysfunction in the 3xTg-AD mouse brain.

### Expression of hTFAM improves cognitive impairment in 3xTg-AD mice, and decreases intracellular Aβ accumulation in the brain

Hemizygous 3xTg-AD (ADh/WT) mice start to exhibit a decline in memory retention in the Morris water maze (MWM) as early as 6 months of age^23^. As shown in Fig. 1c, we first performed the probe test in the MWM (d2 protocol) using 11–12-month-old Non-Tg and ADh/WT mice, and confirmed that ADh/WT mice indeed exhibited significant decline in memory retention 24 h after the last training session compared with Non-Tg mice. The performance of ADh/hTFAMh and Non-Tg mice were similar in both their latency to cross the platform location (Fig. 1c) and number of crossings (Fig. 1d) indicating that hTFAM suppresses the decline of memory retention in 3xTg-AD mice. No significant differences were detected among any of the genotypes in swim speed and total swimming distance, indicating equal swimming ability (Supplementary Fig. S2). We next compared the performance of ADh/WT and ADh/hTFAMh mice in the 5-day training session (d5 protocol; Fig. 1e). ADh/WT mice exhibited poor memory retention, whereas ADh/hTFAMh mice performed significantly better. Based on these results, we conclude that hTFAM expression ameliorates the cognitive impairment seen in 3xTg-AD mice.

**Figure 2:**
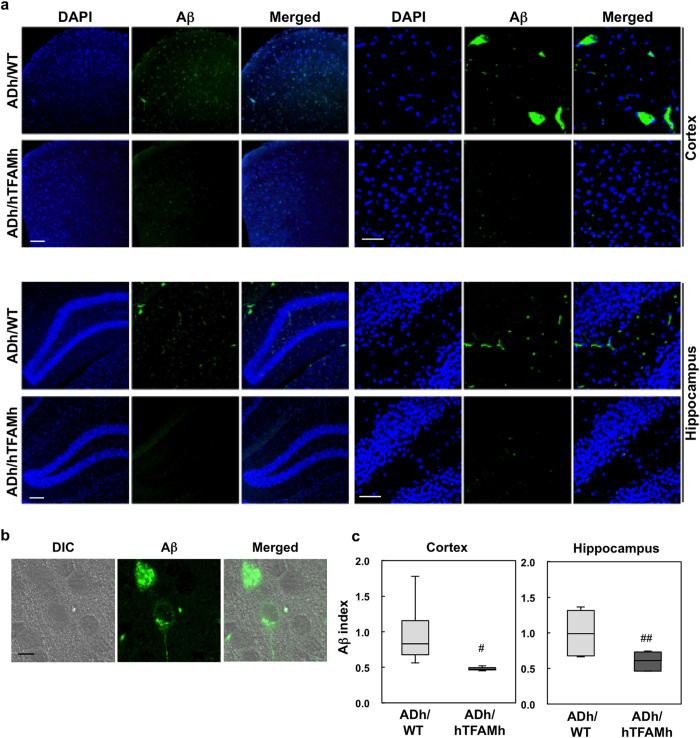
hTFAM suppresses Aβ accumulation in ADh/WT mice. (**a**) Immunofluorescence microscopy of Aβ in cortex and hippocampus of 13–14-month-old ADh/WT and ADh/hTFAMh mice using anti-human Aβ (**N**). Nuclei are stained with DAPI. Scale bar, 20 μm. (**b**) Intracellular localization of Aβ in the ADh/WT cortex. Cell morphology was confirmed by differential interference contrast microscopy (DIC) with Aβ immunofluorescence. Scale bar, 5 μm. (**c**) Quantitative measurement of Aβ immunofluorescence. Aβ immunofluorescence was measured as described in Supplementary Fig. S3, and the relative intensity (Aβ index) is shown in whisker-box plot. Kruskal–Wallis rank sum test: cortex, ^#^*p* = 0.0433, hippocampus, ^##^*p* = 0.0209, n = 3–4.

Immunofluorescence microscopy of Aβ using anti-Aβ (82E1), which recognizes the N-terminus of the Aβ-peptide, revealed that ADh/WT mice mainly accumulated intracellular Aβ in the cortex and hippocampus (Fig. 2a,b). The Aβ immunofluorescence intensity was measured as shown in Supplementary Fig. S3 and is presented as an Aβ index. The Aβ index was significantly decreased in the cortex and hippocampus of ADh/hTFAMh mice (Fig. 2c), indicating that hTFAM expression decreases intracellular Aβ accumulation in the 3xTg-AD mouse brain.

### hTFAM suppresses 8-oxoG accumulation in mtDNA

**Figure 3:**
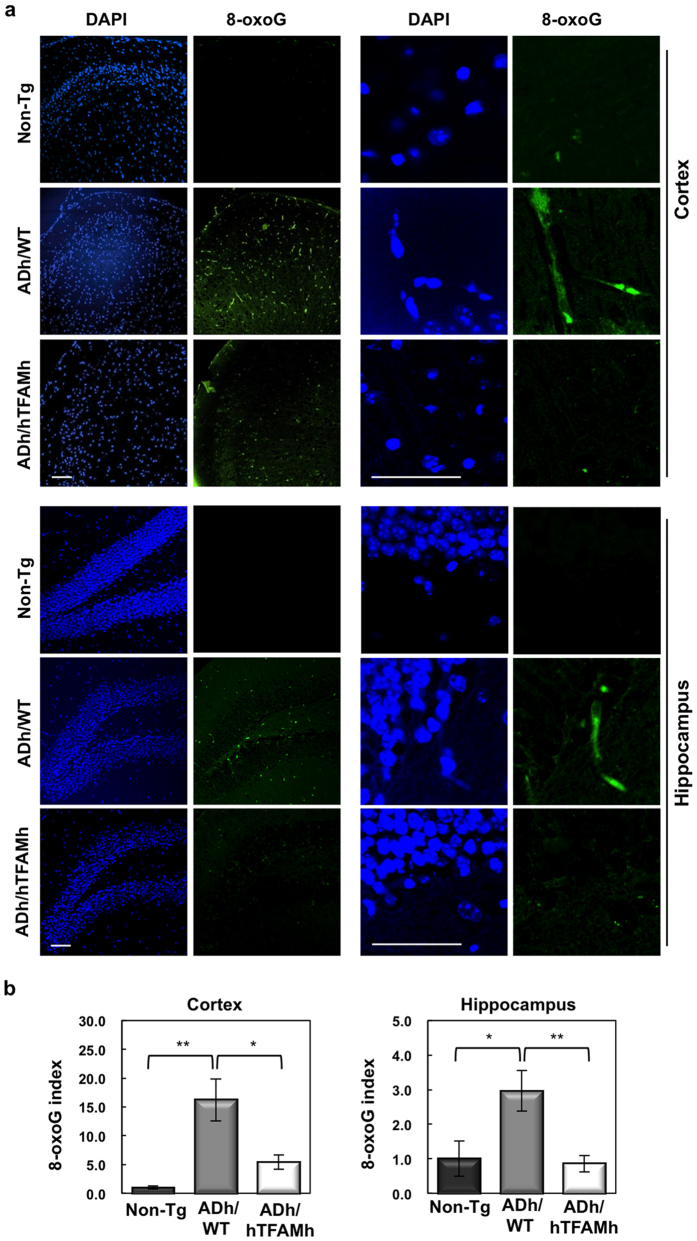
hTFAM markedly decreases accumulation of mitochondrial 8-oxoG in ADh/WT mice. (**a**) Immunofluorescence microscopy of 8-oxoG in cortex and hippocampus of 13–14-month-old ADh/WT and ADh/hTFAMh mice. hTFAM decreases mitochondrial 8-oxoG in the cortex and hippocampus. Scale bar, 20 μm. (**b**) Quantitative measurement of 8-oxoG immunofluorescence. 8-OxoG immunofluorescence was measured as described in Supplementary Fig. S3, and the relative intensity of 8-oxoG immunoreactivity (8-oxoG index) is shown in bar graphs. Cortex, one-way ANOVA, *p* = 0.0028. Tukey-Kramer’s HSD test: *p = 0.0193, **p = 0.0025; Hippocampus, one-way ANOVA, *p* = 0.0198. Tukey-Kramer’s HSD test: *p = 0.0394, **p = 0.0277. Data represent means ± SEM, n = 4.

Because mitochondrial dysfunction increases ROS production, we next immunohistochemically examined the accumulation of 8-oxoG in mtDNA, as a marker of oxidation in mtDNA. Brain slices were pretreated with RNase, to avoid detecting 8-oxoG in RNA, and then reacted with anti-8-oxo-dG. Under this condition, cytoplasmic 8-oxoG immunoreactivity represents 8-oxoG accumulation in mtDNA^24^. As shown in Fig. 3a and Supplementary Fig. S4, the ADh/WT brain exhibited strong 8-oxoG immunoreactivity in both the cortex and hippocampus. The 8-oxoG immunoreactivity mainly co-localized with mouse TFAM (Supplementary Fig. S5a), which may reflect a higher binding affinity of TFAM to C:8-oxoG than to C:G^25^, and was abolished by pretreatment with MutM, which excises 8-oxoG opposite cytosine in DNA^24^ (Supplementary Fig. S5b). These results demonstrate that 8-oxoG accumulates in mtDNA in the ADh/WT brain. In ADh/hTFAMh mice, 8-oxoG immunoreactivity was low, and quantitative measurement revealed that levels of 8-oxoG in the mtDNA of ADh/hTFAMh brains were significantly reduced compared with those in ADh/WT brains to 33% in the cortex and 29% in the hippocampus (Fig. 3b).

### Microarray analysis reveals altered gene expression in the hippocampus of ADh/hTFAMh mice

**Figure 4:**
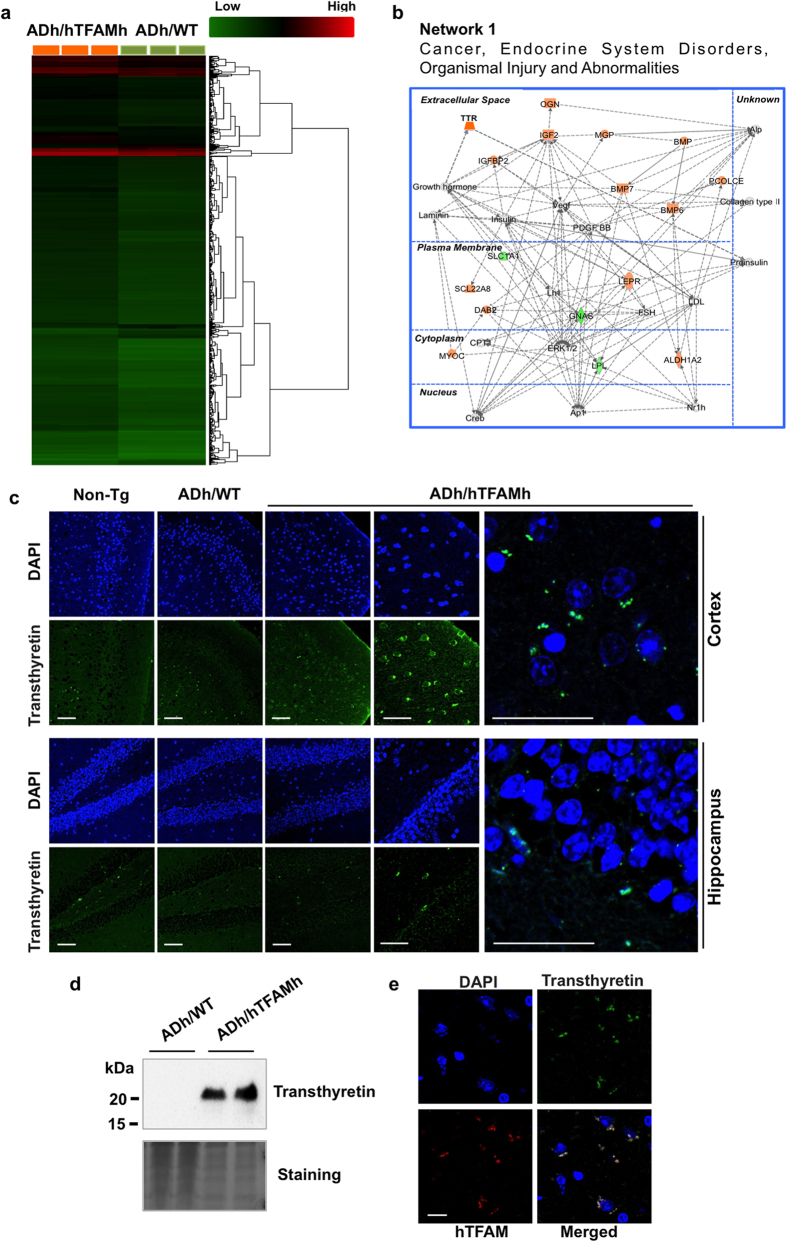
hTFAM markedly increases expression of a set of genes in ADh/WT mice. (**a**) Hierarchical portioning and clustering of 166 transcript clusters demonstrate 1.6-fold or greater difference between hippocampi from ADh/WT and ADh/hTFAMh mice. Student’s t-test *p* < 0.05, n = 3. (**b**) Top significant network. Solid and dashed lines indicate direct and indirect interactions, respectively. Molecular interactions involving only binding were excluded. Downregulated molecules are shown in green and upregulated molecules are shown in red. (**c**) hTFAM increases the level of transthyretin in ADh/WT mice. Scale bar, 20 μm. (**d**) Western blot analysis of extracts prepared from cerebral cortices using anti-transthyretin. Ponceau S staining was used as the loading control (lower panel). (**e**) Double-immunostaining with anti-human TFAM and anti-transthyretin in ADh/hTFAMh cortex. Scale bar, 20 μm.

To delineate the mechanism underlying the amelioration of AD pathophysiology by hTFAM, we next performed gene expression profiling using microarray analysis. A total of 2,565 transcript clusters were identified as significantly different between ADh/hTFAMh and ADh/WT hippocampus. Among them, 166 transcript clusters exhibited 1.6-fold or more difference between the two samples (p < 0.05), and these were further subjected to hierarchical partitioning and clustering (Fig. 4a), revealing that hTFAM expression significantly altered the gene expression profile in 3xTg-AD-h hippocampus. We found that hTFAM expression upregulated 22 annotated clusters (average raw expression level [log_2_] >6.6 in ADh/hTFAMh) and downregulated 20 annotated clusters (average raw expression level [log_2_] >6.6 in ADh/WT) (Supplementary Tables S1 and S2). Among the 42 annotated transcript clusters, 41 genes were functional/pathway eligible genes as determined by the computational gene network prediction tool, Ingenuity Pathway Analysis (IPA). These genes were categorized as genes significantly relevant to cancer, endocrine system disorders, and organismal injury and abnormalities, including 14 upregulated genes (*TTR*, *OGN*, *IGF2*, *IGFBP2*, *MGP*, *BMP*, *BMP7*, *BMP6*, *PCOLCE*, *LEPR*, *SCL22A8*, *DAB2*, *MYOC*, *ALDH1A2*), and three downregulated genes (*SLC1A1*, *GNAS*, *LPL*) (Fig. 4b).

Among the genes whose expression was significantly altered by hTFAM expression, *Ttr* gene encodes transthyretin, which binds Aβ and inhibits its aggregation^26^. We thus examined the levels of the transthyretin protein in the mouse brain using immunofluorescence microscopy and western blotting (Fig. 4c–e). As shown in Fig. 4c, transthyretin immunoreactivity was markedly increased in the cortex, and to a lesser extent in the hippocampus, in the ADh/hTFAMh brain compared with that in the ADh/WT brain, which was essentially the same as that observed in the Non-Tg brain. A 20-kDa polypeptide corresponding to transthyretin was detected in the ADh/hTFAMh cortex by western blotting (Fig. 4d). Transthyretin immunoreactivity was mostly detected in hTFAM-positive cells in the ADh/hTFAMh cortex (Fig. 4e), and hTFAM was mostly expressed in NeuN-positive cells (Supplementary Fig. S1b), suggesting mitochondrial localization of transthyretin in neurons expressing hTFAM.

### Recombinant hTFAM protein prevents mitochondrial dysfunction along with a reduction in 8-oxoG accumulation and mitochondrial DNA damage in AD model human neurons

**Figure 5:**
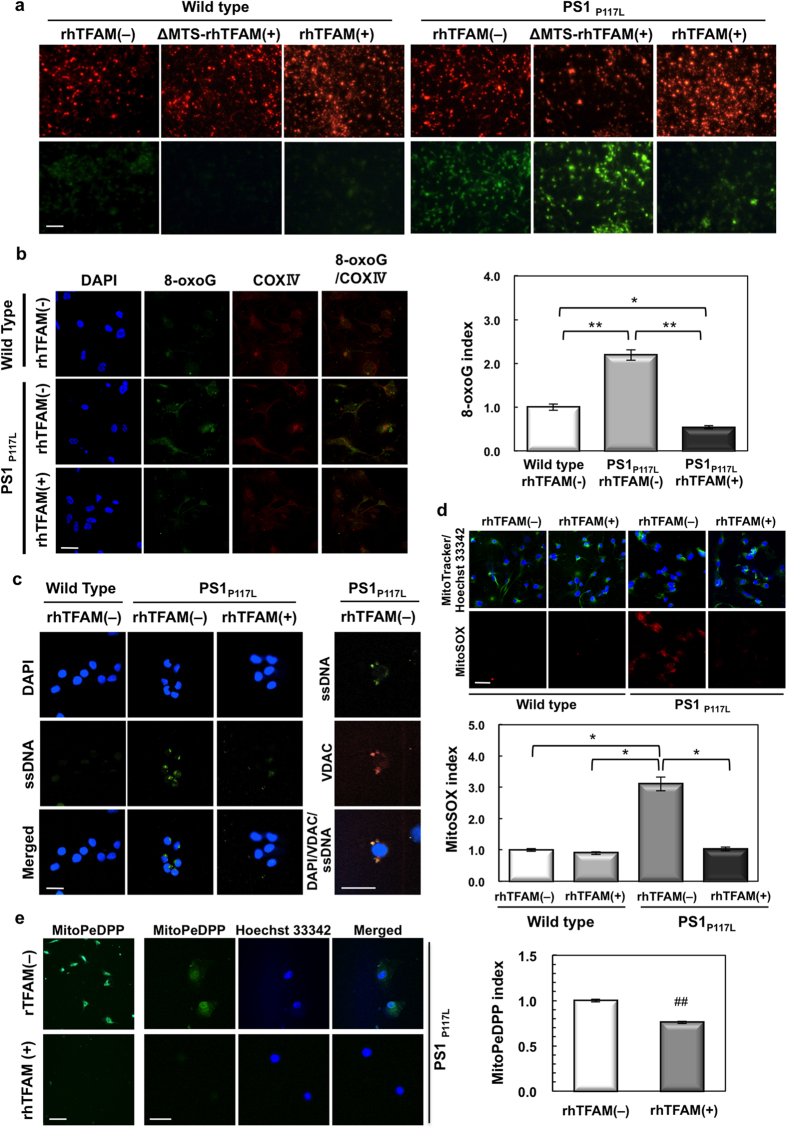
Recombinant human TFAM inhibits mitochondrial dysfunction and 8-oxoG accumulation in a human neuron model of AD. (**a**) Recombinant human TFAM [rhTFAM ( + )], but not mutant rhTFAM (ΔMTS-rhTFAM) lacking MTS, ameliorates mitochondrial dysfunction in PS1_P117L_ cells. Mitochondrial membrane potential was detected using JC-1. Intensities of green fluorescence in ΔMTS-rhTFAM-treated PS1_P117L_ neurons were as high as those seen in control PS1_P117L_ neurons (rhTFAM[−]), whose levels were higher than those observed in wild-type neurons with or without rhTFAM or ΔMTS-rhTFAM. Upper panels: JC-1 aggregates with intense red fluorescence, indicating energized mitochondria with high membrane potentials. Lower panels: JC-1 monomers with green fluorescence, indicating damaged mitochondria with low membrane potentials. Scale bar, 100 μm. (**b**) rhTFAM decreases mitochondrial 8-oxoG in PS1_P117L_ cells. COX IV was used as a mitochondrial marker. Fluorescence intensities of 8-oxoG in 50 cells were measured, and the 8-oxoG index is shown in the bar graph. One-way ANOVA, *p* < 0.0001. Tukey-Kramer HSD test, *p < 0.0026, **p < 0.0001. Data represent means ± SEM. (**c**) rhTFAM inhibits single-strand breaks in mitochondrial DNA in PS1_P117L_ cells. VDAC was used as a mitochondrial marker. (**d**) rhTFAM decreases mitochondrial superoxide production in PS1_P117L_ cells. Wild-type and PS1_P117L_ cells were treated with or without rhTFAM for 24 h, and then incubated with MitoTracker Green, a mitochondrial marker (upper panels, green) and MitoSOX, a mitochondrial superoxide indicator (lower panels, red). Nuclei were counterstained with Hoechst 33342 (upper panels, blue). Fluorescence intensities of MitoSOX in 50 cells were measured, and the MitoSOX index is shown as a bar graph. Two-way ANOVA, *p* < 0.0001. Tukey’s HSD test, *p < 0.0001. Data represent means ± SEM. (**e**) rhTFAM protein suppresses mitochondrial membrane lipid peroxidation in PS1_P117L_ cells. MitoPeDPP, a fluorescent probe specific for lipophilic peroxide in the mitochondrial inner membrane was applied to PS1_P117L_ cells with or without rhTFAM treatment. Nuclei were counterstained with Hoechst 33342. Fluorescence intensities of MitoPeDPP in 50 cells were measured, and the MitoPeDPP index is shown as a bar graph. Student’s *t*-test: ^##^*p* < 0.0001. Data represent means ± SEM. Scale bars, 40 μm (**b**–**e**).

To elucidate the mechanism by which hTFAM ameliorates AD pathophysiology, we next examined the effect of recombinant hTFAM protein in a human cell culture model of AD using neurons derived from iPSCs (Fig. 5). We used cholinergic neurons derived from human iPSCs established from normal subject, in which a wild-type or mutant (PS1_P117L_) copy of the *PSEN1* gene was introduced. Immunostaining of the neuronal marker MAP2 revealed that more than 95% of the cultured cells were MAP2-positive in both wild-type and PS1_P117L_ samples (Supplementary Fig. S6). We prepared recombinant hTFAM (rhTFAM) and ΔMTS-rhTFAM protein, the former being wild-type hTFAM, and the latter being mutant hTFAM lacking the mitochondrial targeting signal (MTS), allowing localization in nuclei but not in mitochondria. The rhTFAM protein rapidly incorporates into mitochondria in cultured cells when added into the medium, and thus contributes to maintaining mitochondrial function^27^.

First, the mitochondrial membrane potential was detected using JC-1 24 h after adding ΔMTS-rhTFAM or rhTFAM protein into the medium (Fig. 5a). JC-1 forms red fluorescent aggregates in energized mitochondria with high membrane potentials, whereas it dissociates to monomers with green fluorescence at low membrane potential. Wild-type neurons emitted only red fluorescence regardless of the absence or presence of wild-type or mutant rhTFAM protein in the media for 24 h. By contrast, PS1_P117L_ neurons emitted reduced red fluorescence but much stronger green fluorescence in the absence or presence of mutant ΔMTS-rhTFAM protein, indicating mitochondrial dysfunction and lowered membrane potential. However, in the presence of wild-type rhTFAM protein, PS1_P117L_ neurons emitted mainly red fluorescence, indicating that wild-type rhTFAM efficiently suppressed mitochondrial dysfunction caused by the PS1_P117L_ mutation.

Next, we found that PS1_P117L_ neurons exhibited an increase in mitochondrial 8-oxoG immunoreactivity and that rhTFAM treatment markedly decreased this immunoreactivity in PS1_P117L_ neurons (Fig. 5b). We thus concluded that rhTFAM protein prevents mitochondrial dysfunction and accumulation of 8-oxoG in mtDNA or ROS production in PS1_P117L_ neurons.

To examine whether rhTFAM protects mtDNA in PS1_P117L_ neurons, we detected single-strand breaks in cellular DNA using an anti-single-stranded DNA (ssDNA) antibody. Immunofluorescence microscopy revealed strong cytoplasmic ssDNA immunoreactivity in PS1_P117L_ neurons, but not in wild-type neurons in the absence of the rhTFAM protein (Fig. 5c), which co-localized with voltage-dependent anion channels (VDAC), a mitochondrial marker.

We then examined mitochondrial superoxide production in each neuron by using MitoSOX, a mitochondrial superoxide indicator (Fig. 5d). We found that PS1_P117L_ neurons produced significantly higher levels of superoxide in mitochondria than did wild-type neurons in the absence of rhTFAM. Treatment with rhTFAM efficiently decreased the level of superoxide in mitochondria of PS_P117L_ neurons to that observed in wild-type neurons. Moreover, we confirmed that rhTFAM also suppressed mitochondrial membrane lipid peroxidation in PS_P117L_ neurons by using MitoPeDPP, a probe specific for lipophilic peroxide in the mitochondrial inner membrane^28^ (Fig. 5e).

These results indicate that mtDNA, but not nuclear DNA, is damaged by the ROS produced in PS1_P117L_ neurons, and the rhTFAM protein effectively protects mtDNA from ROS, thereby maintaining mitochondrial function in a human neuron model of AD.

### Recombinant hTFAM increases the expression of transthyretin and suppresses intracellular Aβ accumulation in human AD model neurons

**Figure 6:**
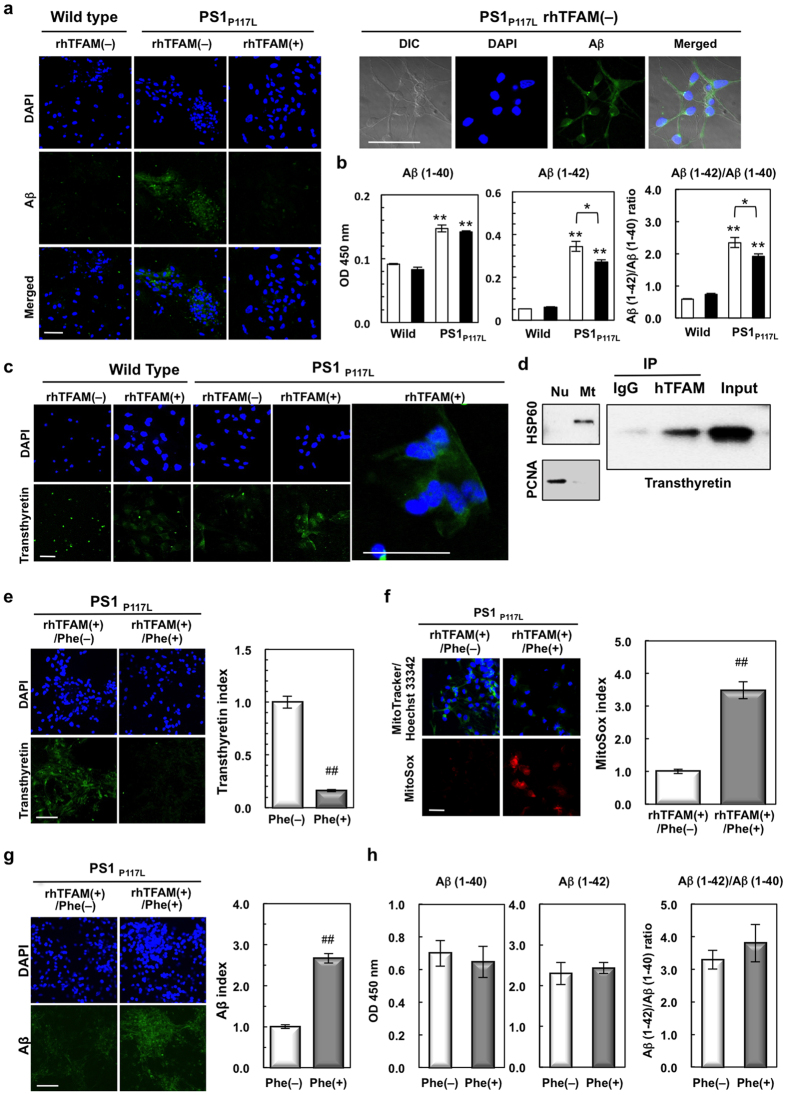
Recombinant human TFAM inhibits Aβ accumulation in a human neuron model of AD with increased expression of transthyretin. (**a**) rhTFAM decreases the accumulation of Aβ in PS1_P117L_ cells. Wild-type and PS1_P117L_ cells were treated with (rhTFAM[+]) or without rhTFAM (rhTFAM[-]) for 24 h. (**b**) rhTFAM has little effect on Aβ secretion in PS1_P117L_ cells. Secretion of Aβ(1–40) and Aβ(1–42) was measured by ELISA using the supernatant 24 h after the treatment of 100 nM ΔMTS_rhTFAM (open bars) or rhTFAM (closed bars) protein. Means ± SEM, n = 4. Two-way ANOVA, *p* < 0.0001. Tukey’s HSD test, **p < 0.0001 vs. wild-type cells (wild) with or without treatment. *p < 0.05. (**c**) rhTFAM increases transthyretin expression in PS1_P117L_ but not in wild-type neurons. (**d**) Interaction of transthyretin and rhTFAM in mitochondria of PS1_P117L_ cells treated with rhTFAM. PS1_P117L_ cells were treated with 100 nM rhTFAM for 24 h and then fractionated into mitochondrial (Mt) and nuclear (Nu) fractions (left panels). Proliferating nuclear antigen (PCNA, 35.5 kDa) or heat shock protein 60 (HSP60, 60 kDa) was detected as a nuclear or mitochondrial marker, respectively. Co-immunoprecipitation of transthyretin was performed using anti-hTFAM or normal IgG as a negative control (IgG) and the mitochondrial fraction prepared from PS1_P117L_ cells treated with rhTFAM (right panels). (**e**) Phenylalanine significantly decreased expression of transthyretin in rhTFAM-treated PS1_P117L_ cells. Cells were treated with 100 nM rhTFAM in the presence (Phe[+]) or absence (Phe[–]) of phenylalanine. Transthyretin index is shown in the bar graph. Student’s *t*-test, ^##^*p* < 0.0001. (**f**) Phenylalanine increased ROS production in rhTFAM-treated PS1_P117L_ cells. Student’s *t*-test, ^##^*p* < 0.0001. (**g**) Phenylalanine increased Aβ immunofluorescence in rhTFAM-treated PS1_P117L_ cells. Aβ index is shown in the bar graph. Student’s *t*-test, ^##^*p* < 0.0001. Data represent means ± SEM from 50 cells. (**h**) Phenylalanine did not alter Aβ secretion in rhTFAM-treated PS1_P117L_ cells. No significant alteration was observed for the Aβ (1–42)/Aβ (1–40) ratio or the level of each Aβ peptide. Data represent means ± SD, n = 3. All scale bars (**a**,**c**,**e**,**f**,**g**), 40 μm.

It is likely that hTFAM suppresses Aβ production or its accumulation in mouse models of AD. We thus examined Aβ accumulation and secretion in the human neuron model of AD. As shown in Fig. 6a, compared with wild-type neurons, PS_P117L_ neurons accumulated high levels of intracellular Aβ. However, in the presence of rhTFAM, intracellular Aβ accumulation was suppressed. Secreted Aβ(1–42) or Aβ(1–40) in the medium was measured using conditioned medium obtained 24 h after adding ΔMTS-rhTFAM or rhTFAM protein to the medium. The PS1_P117L_ neurons showed significantly greater increased secretion of Aβ(1–42) than Aβ(1–40) when compared with the wild type in the presence of ΔMTS-rhTFAM (Fig. 6b). In the presence of the rhTFAM protein, the levels of Aβ(1–42) or the relative ratio of Aβ(1–42)/Aβ(1–40) in PS1_P117L_ neurons was slightly, but significantly, decreased. These results indicate that hTFAM efficiently reduces intracellular Aβ accumulation, with a slight reduction in its production, but not secretion, as observed in the ADh/hTFAMh brain (Fig. 2).

To clarify how rhTFAM reduces intracellular Aβ accumulation in PS1_P117L_ neurons, we examined whether rhTFAM increases expression of transthyretin in the human neuron model of AD, as was observed in the ADh/hTFAMh mouse brain. In Fig. 6c, transthyretin immunoreactivity was detected in both wild-type and PS1_P117L_ neurons in the absence of recombinant protein, and rhTFAM markedly increased transthyretin immunoreactivity in PS1_P117L_ neurons, consistent with the findings in ADh/hTFAMh mice. By contrast, rhTFAM had no apparent effect on the level of transthyretin in wild-type neurons, suggesting that rhTFAM increases the level specifically in the neuron model of AD. Because cytoplasmic transthyretin was co-localized with hTFAM (Supplementary Fig. S7), we examined whether transthyretin interacts with rhTFAM using co-immunoprecipitation–western blot analysis of the mitochondrial fraction prepared from PS1_P117L_ neurons exposed to rhTFAM (Fig. 6d, left panels). Immunoprecipitation with anti-hTFAM, but not normal IgG, simultaneously recovered a substantial amount of transthyretin, indicating complex formation between rhTFAM and transthyretin in mitochondria of PS1_P117L_ neurons (Fig. 6d, right panels).

We next examined whether phenylalanine cancels the effects of rhTFAM treatment on PS1_P117L_ neurons, because phenylalanine has been shown to reduce the expression of transthyretin by decreasing transcriptional activity of the transthyretin promoter site^29^. When PS1_P117L_ neurons were exposed to rhTFAM in the presence of 0.9 mM phenylalanine, immunofluorescence intensity for transthyretin was significantly decreased (Fig. 6e), and the MitoSox index was reversed to the levels seen in PS1_P117L_ neurons without rhTFAM treatment (Fig. 6f), indicating that phenylalanine cancelled the rhTFAM effect to reduce the mitochondrial superoxide production. Moreover, phenylalanine increased intracellular Aβ accumulation in PS1_P117L_ cells treated with rhTFAM, without changing Aβ secretion or the ratio of secreted Aβ(1–42)/Aβ(1–40) (Fig. 6g,h).

These results strongly suggest that increased expression of transthyretin is tightly associated with the mechanisms by which hTFAM efficiently suppresses AD pathology and cognitive impairment caused by Aβ accumulation.

### Recombinant human TFAM improves neuritogenesis with increased expression of neuritogenesis-related genes in a neuron model of AD

To delineate the mechanism by which rhTFAM suppresses AD pathology in the neuron model of AD, we first performed microarray analyses using PS1_P117L_ and wild-type neurons. We identified 7,908 transcript clusters showing a significant difference between PS1_P117L_ and wild-type neurons (ANOVA, *p* < 0.05). Among them, 172 transcript clusters exhibited 12-fold or more difference between the two samples, and these were subjected to hierarchical partitioning and clustering (Supplementary Fig. S8a), revealing altered expression profiles in the neuron model of AD, with 148 clusters downregulated (average raw expression level [log_2_] >6.6 in the wild type) (Supplementary Table S3). Among them, 136 genes were functional/pathway eligible genes in the IPA. As shown in Supplementary Table S4, we identified genes that were significantly enriched in cellular assembly and organization-related functions. From these, 21 genes were sub-categorized as genes significantly relevant to cellular assembly and organization, cellular function and maintenance, and tissue development (Supplementary Fig. S8b). Next, we compared the altered gene expression profile in PS1_P117L_ neurons with that previously obtained from comparative analysis between human AD and non-AD brains^6^. We identified 37 genes that were commonly altered between the two studies (Supplementary Fig. S8c, Supplementary Table S5). These results indicate that PS1_P117L_ neurons are suitable as a human iPSC-derived neuron model of AD.

**Figure 7:**
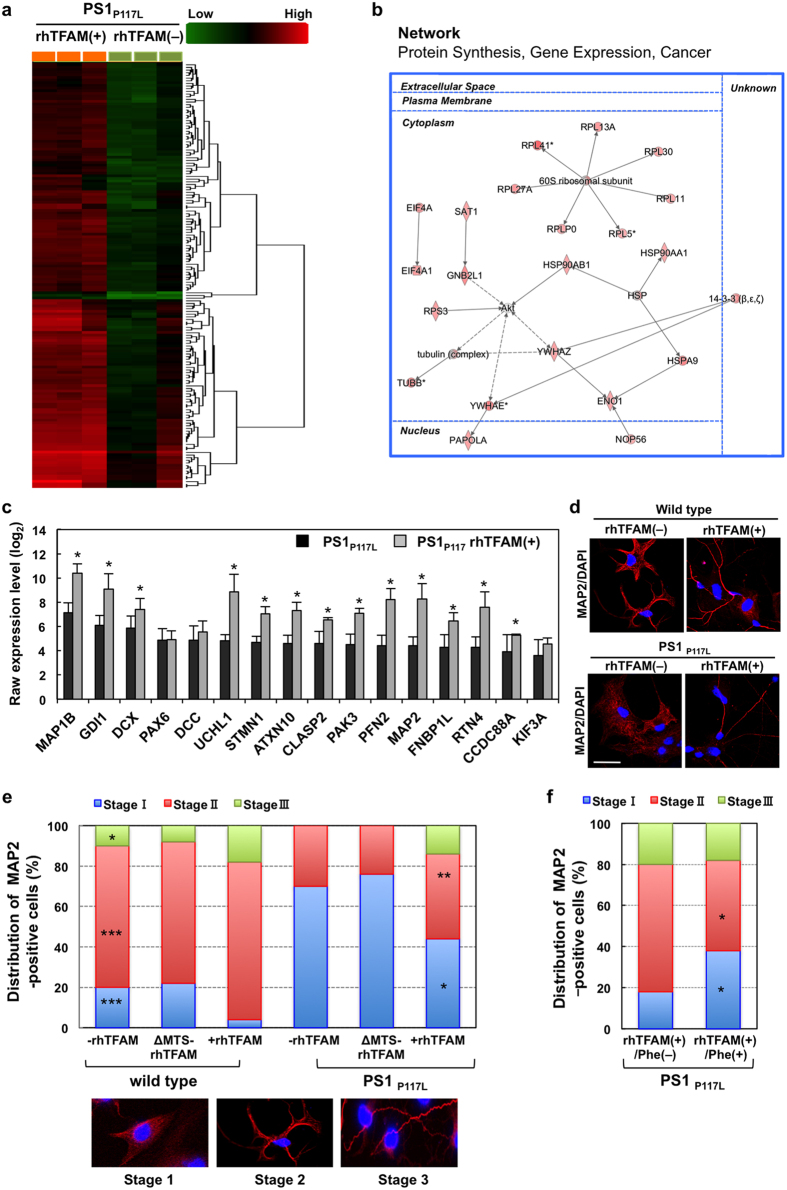
Recombinant human TFAM improves neuritogenesis with increased expression of neuritogenesis-related genes in a neuron model of AD. (**a**) Hierarchical partitioning and clustering of 138 clusters exhibited 8-fold or more increased expression (average raw expression level [log_2_] >6.6 in PS1_P117L_ + rhTFAM), in PS1_P117L_ cells with rhTFAM treatment. Student’s t-test: *p* < 0.05, Means ± SD, n = 3. (**b**) Top significant network consisted of 25-upregulated genes among 132 functional/pathway eligible genes in the IPA. (**c**) Effect of rhTFAM on the 16 neuritogenesis-related gene expression in PS1_P117L_ cells. Student’s t-test: *p* < 0.05. (**d**) rhTFAM improves neuritogenesis. Immunofluorescence microscopy of iPSCs-derived neurons treated with or without rhTFAM, using anti-MAP2. Nuclei are stained with DAPI. Scale bar, 20 μm. (**e**) MAP2-positive cells treated with or without rhTFAM or ΔMTS-rhTFAM were classified into three stages: stage 1, lacking neurites; stage 2, one or more minor neurites; stage 3, one neurite at least twice as long as any other. Fifty MAP2-positive cells in each culture condition were examined, and their distribution is shown in bar graphs. Fisher’s Exact test: *p = 0.0281, **p = 0.0062, ***p < 0.0001 versus PS1_P117L_ rhTFAM(–). (**f**) Phenylalanine inhibits neuritogenesis in rhTFAM-treated PS1_P117L_ cells. Fifty MAP2-positive cells in each culture condition were examined, and their distribution is shown in bar graphs. Fisher’s exact test, *p = 0.0017 vs. rhTFAM(+)/Phe(−).

Next, we performed microarray analyses using PS1_P117L_ neurons with or without rhTFAM treatment. We found 5,920 transcript clusters showing a significant difference in the presence or absence of rhTFAM in PS1_P117L_ neurons. Among them, 138 annotated transcript clusters exhibit 8-fold or more increased expression in the presence of rhTFAM (Supplementary Table S6). These clusters were subjected to hierarchical partitioning and clustering (Fig. 7a), and revealed significantly altered expression profiles in the neuron model of AD following treatment with rhTFAM. Among them, 132 genes were functional/pathway eligible genes in the IPA. The top significant network consisted of 25 upregulated genes, which associate with protein synthesis, gene expression, and cancer (*RPL13A*, *RPL41*, *RPL27A*, *RPLP0*, *RPL5*, *RPL11*, *RPL30*, *60S ribosomal subunit*, *HSP90AA1*, *HSP*, *HSP90AB1*, *HSPA9*, *ENO1*, *14-3-3*, *YWHAZ*, *RPS3*, *Tubulin*, *SAT1*, *GNB2L1*, *EIF4A*, *EIF4A1*, *TUBB*, *YWHAE*, *NOP56*, *PAPOLA*) (Fig. 7b). From these microarray analyses, we found that the expression levels of 16 genes associated with neuritogenesis were decreased in PS1_P117L_ neurons compared with those in the wild type, and that hTFAM significantly increased 13 of these genes (Supplementary Fig. S9, Fig. 7c).

To determine whether mitochondrial dysfunction caused by the PS1_P117L_ mutation impairs neuritogenesis, the morphology of PS1_P117L_ and wild-type neurons was examined by MAP2-immunofluorescence microscopy (Fig. 7d). As shown in the upper panels of Fig. 7e, each neuron was classified into three stages based on the pattern of their neurite outgrowth, as previously described^30^: stage 1, lacking neurites; stage 2, one or more minor neurites; stage 3, one neurite at least twice as long as any other. In the absence of rhTFAM, wild-type neurons and PS1_P117L_ neurons were approximately 10% and 0% in stage 3, 70% and 30% in stage 2, and 20% and 70% in stage 1, respectively (Fig. 7e, bar graphs), suggesting that neuritogenesis was impaired in this human neuron model of AD. Treatment with hTFAM increased the population of neurons in stage 2 (from 30% to 42%) and stage 3 (from 0% to 14%) in PS1_P117L_ neurons. By contrast, treatment with ΔMTS-rhTFAM had no obvious effect on neuritogenesis in both PS1_P117L_ and wild-type neurons (Fig. 7e, bar graphs). Finally, we observed that phenylalanine significantly increased the population of PS1_P117L_ neurons in stage 1 (from 18% to 38%) and decreased the population of neurons in stage 2 (from 62% to 44%) even in the presence of rhTFAM (Fig. 7f), indicating that phenylalanine cancelled the neuritogenesis improved by rhTFAM.

These results suggest that mitochondrial dysfunction leads to impaired neuritogenesis and that hTFAM has a crucial role in suppressing AD pathology and cognitive dysfunction by ameliorating the impairment of neuritogenesis.

## Discussion

In the present study, we demonstrated that hTFAM effectively suppresses the vicious cycle of the neuronal mitochondrial dysfunction that occurs during AD pathogenesis by increasing the expression of transthyretin, thereby ameliorating the AD pathophysiology observed in both human iPSC-derived AD neuron and 3xTg-AD mouse models.

**Figure 8:**
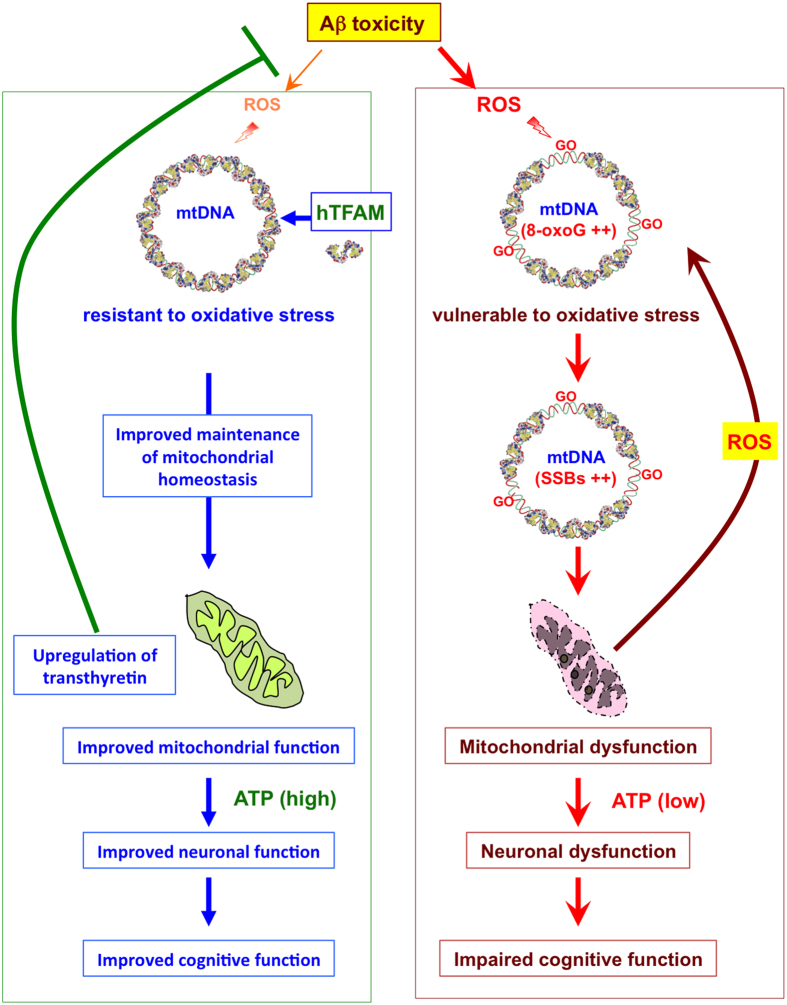
TFAM breaks the mitochondria-mediated vicious cycle of Alzheimer’s disease. hTFAM binds mtDNA and forms a nucleoid-like structure, thereby protecting mtDNA from ROS generated under conditions of Aβ toxicity. Improved maintenance of mitochondrial homeostasis reduced ROS generation from mitochondria, and results in suppression of oxidative modification of transthyretin and/or increase in its expression. Transthyretin reduces intracellular Aβ accumulation, thus further contributes to the break of mitochondria- mediated vicious cycle. hTFAM also improves neuronal function such as neuritogenesis through suppression of ATP depletion caused by mitochondrial dysfunction and increased expression of neuritogenesis-related genes. hTFAM effectively suppresses the vicious cycle of the neuronal mitochondrial dysfunction, thereby ameliorating the AD pathophysiology.

Treatment with hTFAM markedly improved mitochondrial function and significantly reduced accumulation of 8-oxoG in mtDNA and intracellular Aβ in both the 3xTg-AD mouse and human iPSC-derived AD neuron models. These data indicated that hTFAM effectively protects mtDNA from the ROS generated in damaged mitochondria under conditions of Aβ accumulation, and thus suppresses the vicious cycle of ROS generation and mitochondrial dysfunction. The hTFAM-induced reduction in Aβ accumulation may also contribute to suppression of the vicious cycle, and these combined effects may be the reason hTFAM expression effectively ameliorates AD pathophysiology (Fig. 8).

The brain utilizes a vast amount of energy to sustain its basic functions, such as maintaining or re-establishing of membrane potentials, signaling, and synthesis of macromolecules involved in neuritogenesis and synaptogenesis. While an adult human brain typically weighs only about 2% of the body weight, a resting brain consumes more than 20% of all the oxygen, thus indicating a 10-fold greater energy requirement than other tissues. This high demand for energy in the brain is mainly achieved by ATP production during oxidation of glucose or oxidative phosphorylation in the mitochondria^31^,^32^. In AD brains, two essential glucose metabolic pathways in mitochondria: Krebs cycle and oxidative phosphorylation are known to be distressed. Abnormal Krebs cycle or/and oxidative phosphorylation causes not only glucose hypometabolism resulting in ATP depletion in brain but also the increased generation of ROS, oxidative damage, and programmed cell death such as apoptosis. Because mitochondria are also the main location that suffers from ROS, oxidative stress further exacerbates mitochondrial dysfunction and this vicious cycle is more prone to occur and have been demonstrated to be an event occurring before the appearance of senile plaques and the onset of clinical manifestations^7^,^33^,^34^,^35^. Human TFAM has an ability to bind to DNA in a sequence-independent manner and is abundant enough to cover whole region of mitochondrial DNA, and thereby TFAM stabilizes mitochondrial DNA through formation of nucleoid and regulates the amount of mitochondrial DNA^15^,^16^. Overexpression of human TFAM in mice increases the amount of mitochondrial DNA and dramatically ameliorates the cardiac dysfunctions^20^, and also reverse of age-dependent memory impairment and mitochondrial DNA damage in brain^19^. The maintenance of integrity of mitochondrial DNA is thus important for keeping proper cellular functions both under physiological and pathological conditions.

The present study clearly shows that hTFAM efficiently breaks the mitochondria-mediated vicious cycle in both AD model neurons and mouse brains, resulting in an effective improvement of the AD pathophysiology including Aβ accumulation and cognitive dysfunction (Fig. 8). In the mice used to model AD (3xTg-AD-h) in the present study, it has been reported that synaptic dysfunction with mitochondrial impairment, but not neuronal loss or apoptosis, is the main pathophysiology resulting in impaired cognitive function^21^,^22^. The lack of neuronal loss in the 3xTg-AD-h mice may be the reason for the significant improvement in cognitive dysfunction by hTFAM. We thus demonstrated for the first time that an establishment of the mitochondria-mediated vicious cycle is one of the most critical processes during the progression of AD pathology. It is noteworthy that not only ROS production but also intracellular Aβ accumulation is significantly suppressed by hTFAM, probably through increased expression of transthyretin which is known to suppress Aβ aggregation^26^. Moreover, poor neuritogenesis found in the iPSC-derived AD neuron model which is also likely to be a consequence of mitochondrial dysfunction^30^, is also efficiently improved by hTFAM (Figs 5a and 7d,e). Inhibition of transthyretin mRNA expression by phenylalanine abolished the improvement in neuritogenesis with a significantly reduced level of transthyretin (Figs 6e and 7f), which is likely to be associated with hTFAM in mitochondria (Fig. 6d), suggesting that increased expression of transthyretin may also contribute to maintenance of mitochondrial homeostasis. Neurogenesis, including neuritogenesis, is an event independent from neurodegeneration *in vivo,* and neurogenesis is also known to be impaired in the 3xTg-AD brain^36^. If hTFAM can improve neurogenesis *in vivo*, it may be one of the mechanisms by which hTFAM significantly ameliorates cognitive dysfunction in the mouse model of AD (Fig. 1c–e). Improvement of neuritogenesis by rhTFAM may also suggest that degeneration of neurites in damaged neurons can be efficiently suppressed by hTFAM *in vivo*, although these issues need to be addressed in future studies.

We showed for the first time that human iPSC-derived AD neuron model carrying PS1_P117L_ mutation (PS1_P117L_ AD neurons) exhibit increased Aβ accumulation, mitochondrial dysfunction, and impaired neuritogenesis compared with wild-type neurons. Gene expression profiling revealed that the PS1_P117L_ AD neurons show significantly altered expression of genes associated with microtubule dynamics, growth of neuritis or neuritogenesis, formation of cellular protrusion or plasma membrane projections (Supplementary Table S4), including 16 neuritogenesis-related genes (*ATXN10, CCDC88A, CLASP2, DCC, DCX, FNBP1L, GDI1, KIF3A, MAP1B, MAP2, PAK3, PAX6, PFN2, RTN4, STMN1, UCHL1*) whose expression is significantly downregulated in the AD model neurons (Supplementary Fig. S9). Among them, expression levels of 8 genes (*ATXN10*, *DCC*, *KIF3A*, *MAP1B*, *PAK3*, *PFN2*, *RTN4, UCHL1*) were also significantly decreased in AD hippocampi (GEO DataSets GSE36980)^6^. Downregulation of *MAP1B* and *UCHL1* in the AD brain have been shown previously^37^,^38^, and moreover, PAK3^39^,^40^ and RTN4^41^,^42^ were known to be associated with AD pathology. Taken together, the PS1_P117L_ AD neurons reproduce major characteristics observed in neurons in AD brain, and are useful for understanding molecular mechanisms of AD pathology in neurons. Many studies have shown the role of mitochondria on neuritogenesis through calcium handling and ATP production^43^,^44^. Recently, we demonstrated that 8-oxoG accumulation in neuronal mtDNA results in mitochondrial dysfunction, and leads to poor neurite outgrowth with decreased complexity of neuritic arborization, using isolated cortical neurons from mice lacking both 8-oxo-dGTP-depleting MTH1 and 8-oxoG excising OGG1^30^, similar to the AD model neurons. In the present study, we showed that rhTFAM efficiently suppresses mitochondrial dysfunction accompanied by reduced accumulation of 8-oxoG and single strand breaks in mtDNA (Fig. 5c), thus resulting in improvement of impaired neuritogenesis (Fig. 7e). Microarray analysis also revealed that rhTFAM efficiently recovers the expression of 13 genes (*MAP1B*, *GDI1*, *DCX*, *UCHL1*, *STMN1*, *ATXN10*, *CLASP2*, *PAK3*, *PFN2*, *MAP2*, *FNBP1L*, *RTN4*, *CCDC88A*) among the 16 neuritogenesis-related genes downregulated in the PS1_P117L_ AD neurons (Supplementary Fig. S9). These results indicate that maintenance of mitochondrial homeostasis that can be achieved by suppression of oxidative damage in mtDNA is crucial for neuritogenesis, as well as for suppression of AD pathogenesis.

Transthyretin (also known as prealbumin) is a transport protein in blood or cerebrospinal fluid for the thyroid hormone thyroxine and retinol-binding protein bound to retinol, and is known to be mainly synthesized in liver and choroid plexus^45^,^46^. Several studies have shown that transthyretin is associated with AD pathogenesis. Transthyretin binds to Aβ(1-42) and Aβ(1-40) and inhibits formation of Aβ oligomer and fibril^26^,^47^, and levels of transthyretin in the cerebrospinal fluid were reported to be reduced in AD patients^48^,^49^. Moreover, it has been demonstrated that transthyretin overexpression suppresses both cognitive dysfunction and Aβ deposition in the cortex and hippocampus in 15-month-old APP23 Tg mice, while transthyretin deficiency in 5.5-month-old APP23 Tg mice increases the Aβ deposition^50^. Recently, neuronal production of transthyretin was reported to be increased in both cortical neurons in AD patient brains and primary cultured cortical and hippocampal neurons from APP23 mouse brains^51^, as we observed in the present study (Fig. 4c–e). Taken together, our results strongly suggest that increased expression of transthyretin triggered by hTFAM in both AD model neurons and mouse brain is protective against Aβ toxicity in these models, thereby ameliorating the AD pathophysiology. Expression levels of transthyretin mRNA in hippocampus of aged 3xTg-AD mice (14-month-old) are equivalent to those in Non-Tg control^6^. However, we noticed that hippocampal expression of transthyretin mRNA is significantly increased in 4-month-old 3xTg-AD brains compared with that in Non-Tg controls (our unpublished observations), suggesting a protective response in the early stage of AD pathogenesis. It has been reported that Aβ42 accumulated in the nuclei directly activated the p53 promoter through binding heat-shock elements (HSE)^52^,^53^. Interestingly, there are two potential HSEs, nTCCn(4 bp)GAAn(5 bp)TTCn at −654 base pairs and nGAAn(4 bp)TTCnAGGn at −393 base pairs upstream from the transcriptional start site of *transthyretin* gene (our unpublished observation). These findings suggest that expression of *transthyretin* gene is also transcriptionally regulated by nuclear Aβ42 in the early stage of AD pathogenesis. Transthyretin has a single free thiol, which is susceptible to modification/oxidation, and oxidative modification of transthyretin decreases its thermodynamic stability^54^,^55^. The level of transthyretin protein was increased in the PS1_P117L_ AD neurons by rhTFAM treatment (Fig. 5c), without increasing its mRNA level, suggesting that reduced ROS generation by rhTFAM results in suppression of oxidative modification of transthyretin and its stabilization. It is likely that TFAM increases the transthyretin level, at least through two independent pathways; however, further studies are needed to elucidate their efficacy and detailed mechanisms.

In conclusion, our results provide new insights into the molecular mechanisms of AD pathogenesis with the mitochondria-mediated vicious cycle, and suggest that breaking the vicious cycle is a potential new therapeutic strategy for AD.

## Methods

### Animals

The homozygous triple-transgenic mouse model of AD (3xTg-AD-H), carrying a homozygous *Psen1*_M146V_ knock-in mutation and the homozygous mutant transgenes for *APP*_Swe_ and *MAPT*_P301L_, were previously established^21^. These 3xTg-AD-H mice were backcrossed to C57BL/6J (Clea Japan, Tokyo, Japan), and the obtained hemizygous 3xTg-AD mice (3xTg-AD-h) were used to re-establish 3xTg-AD-H mice^6^. We generated human TFAM (hTFAM) transgenic mice as described previously^20^, and hemizygous hTFAM transgenic (hTFAM-h) mice and non-transgenic (NT) mice were maintained by crossing hTFAMh mice with C57BL/6J mice. The 3xTg-AD-H mice were mated with hTFAMh mice, and all hemizygous mice (ADh/hTFAMh) and 3xTg-AD-h mice without the hTFAM transgene (ADh/WT) were obtained. Non-transgenic control mice (Non-Tg) were obtained by crossing ADh/WT and NT mice. Genotyping was performed as described in the Supplementary Methods. Male mice were used for behavioral analysis at 11–12 months of age, and for other experiments at 13–14 months of age. All animals were maintained in an air-conditioned, specific pathogen-free room with time-controlled lighting. The handling and killing of all animals was performed in accordance with national prescribed guidelines, and ethical approval for the study was granted by the Animal Experiment Committee of Kyushu University.

### Antibodies

All antibodies used in this study are listed in the Supplementary Methods.

### Tissue processing and Immunohistochemistry

All procedures for tissue processing and immunohistochemistry are described in the Supplementary Methods.

### Western blot analysis

Frozen brain samples were homogenized in 2× sodium dodecyl sulfate (SDS) sample buffer (125 mM Tris-HCl, pH 6.8, 4% SDS, 10% glycerol) using a tissue homogenizer at 4 °C. Samples were diluted in 1× SDS sample buffer (62.5 mM Tris-HCl, pH 6.8, 2% SDS, 5% glycerol), with 2-mercaptoethanol and bromophenol blue, and boiled. The homogenates (20 μg protein) were subjected to western blotting.

### Quantitative immunodetection of mitochondrial 8-oxoG

For immunodetection of 8-oxoG in mtDNA, free-floating brain sections were pretreated as described previously^4^. Briefly, to detect 8-oxoG in mitochondrial DNA, sections were pretreated with RNase (5 mg/mL; Sigma-Aldrich) and were directly subjected to immunohistochemistry assays with the anti-8-oxo-dG antibody (1:100). To verify the specificity of 8-oxoG immunoreactivity, slides were pretreated with 10 μg/mL MutM protein with 8-oxoG DNA glycosylase activity (Sigma-Aldrich) in nicking buffer (10 mM Tris-HCl, 5 μM ZnCl_2_, 0.5 mM DTT, 0.5 mM EDTA, 1.5% [v/v] glycerol, 100 μg/mL bovine serum albumin) diluted in reaction buffer (50 mM Tris-HCl, pH 7.5, 0.1 mM MgCl_2_) at 37 °C for 1 h after RNase treatment. Slides were then subjected to immunofluorescence microscopy analysis using anti-8-oxo-dG (1:100). Confocal microscopy images were acquired using an LSM700 Confocal Microscope system (Carl Zeiss MicroImaging, Tokyo, Japan). The intensity of 8-oxoG immunofluorescence was measured in each digital image using ImageJ 1.49v (NIH) to obtain the 8-oxoG index.

### Histochemical detection of mitochondrial cytochrome c oxidase activity

For detection of mitochondrial cytochrome c oxidase activity in frozen sections, brain sections (100 μm thick) were incubated for 2.5 h at 37 °C in a solution containing cytochrome c (0.5 mg/mL, Sigma-Aldrich, C2506) and DAB (0.5 mg/mL, K-4100, Vector Laboratories Inc., Burlingame, CA, USA), and sucrose (40 mg/mL) in PBS. Sections were washed in PBS, dehydrated with ethanol, immersed in xylene, and mounted on glass slides. Digital images were acquired using an Axioskop2 Plus microscope, equipped with an AxioCam CCD camera and Axiovision 3.1 imaging software (Carl Zeiss MicroImaging, Tokyo, Japan).

### Behavioural analysis

The water maze consisted of a circular polyvinyl chloride pool (100 cm in diameter and 30 cm high) that was filled to a depth of 15 cm with room-temperature tap water (25 ± 1 °C). The performance of mice was scored using a video camera-based computer tracking system (Watermaze3, Actimetrics Inc., Evanston, IL, USA) on a Sony computer, with the camera fixed to the ceiling 2 m above the pool. Mice were trained to swim to a 15-cm-diameter, circular, clear, Plexiglas platform submerged 1 cm beneath the surface of the water that was invisible to the mice while swimming. The platform was kept in the same location throughout training. On each trial, the mouse was placed into the pool at one of four designated start points in a pseudorandom order. Mice were allowed to find and escape onto the submerged platform. If a mouse failed to find the platform within 60 s, it was manually guided to the platform and allowed to remain there for 15 s. After this, each mouse was placed into a holding cage under a warming lamp for 60 s until the start of the next trial. Retention of spatial memory was assessed in two different protocols (d2 and d5). In the d2 protocol, mice were given three training trials on the first day, and probe trials were performed 1.5 h and 24 h after the last training trial. The probe trials consisted of a 60-s free swim in the pool without the platform. The parameters measured during the probe trial included (1) initial latency to cross the platform location, (2) number of platform location crosses, and (3) total swimming time and distance. In the d5 protocol, mice were given three training trials per day for 5 days with a hidden platform, and initial latency to reach the platform and total swimming time and distance were measured. In both protocols, there were no significant differences among genotypes in swim speed.

### Neurons derived from human iPSCs

Cholinergic neurons derived from human iPSCs expressing either a wild-type or mutant (P117L) copy of the *PSEN1* gene (ReproNeuro Ach-AD: RCESDA104, 106, respectively) were obtained from ReproCELL Inc. (Yokohama, Japan). Cells were plated in 12-well plates at a density of 2.0 × 10^5^ cells/mL in the maturation medium according to the manufacturer’s protocol, and incubated for 14 days with changing medium at 3, 7 and 14 days after seeding. Cells were treated for 24 h with wild-type rhTFAM or MTS-lacking mutant rhTFAM (ΔMTS-rhTFAM) protein (100 nM), prepared as described previously^27^. The mitochondrial membrane potential was detected using 5,5′,6,6′-tetrachloro-1,1′,3,3′-tetraethylbenzimidazolylcarbocyanine iodide, (JC-1) (Life Technologies). Cells treated with rhTFAM protein were incubated in the presence of 5 μg/ml JC-1 for 20 min at 37 °C, and the washed twice with PBS before being observed under an Axioskop 2 microscope equipped with an AxioCam camera (Carl Zeiss). To detect mitochondrial 8-oxoG, cells were fixed with methanol, treated with 5 mg/mL RNase at 37 °C for 1 h, followed by denaturation with ice-cold 25 mM NaOH in 50% (v/v) ethanol for 5 min at room temperature. Cells were then incubated with anti-8-oxo-dG or anti-COX IV. Secretion of Aβ(1–42) and Aβ(1–40) in 100 μL of cell culture supernatant was measured using human β amyloid ELISA kits according to the manufacturer’s instructions (296–64401, 292–62301; Wako Pure Chemical Industries Ltd., Osaka, Japan). To determine ROS production in mitochondria, mitochondrial superoxide indicator MitoSOX (Life Technologies Japan) and 3-[4-(perylenylphenylphosphino)phenoxy] propyltriphenylphosphonium iodide (MitoPeDPP), which is a fluorescence probe that specifically reacts with lipid peroxide in the mitochondrial inner membrane^28^ (Dojindo Molecular Technologies, Inc., Kumamoto, Japan), were used. Cells were incubated in the presence or absence of 100 nM rhTFAM protein for 24 h, and were incubated in 0.5 μM MitoSOX together with 0.32 μM MitoTracker Green (Life Technologies) or 0.2 μM MitoPeDPP solution for 20 min at 37 °C. After being washed twice with PBS, the cells were observed using an LSM 700 confocal microscope system. The intensity of MitoSOX immunofluorescence was measured in each digital image using ImageJ 1.49 v. To inhibit the expression of transthyretin, L-phenylalanine (0.9 mM, Sigma-Aldrich) was used^29^.

### Immunoprecipitation

Procedures for immunoprecipitation are described in the Supplementary Methods.

### Gene expression profiling with microarray analysis

Total RNA was isolated using Isogen from the hippocampus, or neurons derived from human iPSCs treated for 24 h with or without rhTFAM protein (100 nM), and 100 ng RNA was subjected to microarray analysis. Expression profiles were determined using mouse or human Gene 1.0 ST array systems according to the manufacturer’s instructions (Affymetrix Inc., Santa Clara, CA, USA). To generate amplified and biotinylated sense-strand DNA targets from the entire expressed transcripts, the Ambion WT Expression kit (Ambion, Austin, TX, USA) and the GeneChip WT Terminal Labeling and Controls kit (Affymetrix) were used. Generated CEL files were imported into the Transcriptome Analysis Console 3.0 software (Affymetrix), and gene-level estimates were obtained for all transcript clusters. The gene-level estimates were further subjected to statistical analysis and hierarchical and partitioning clustering. Transcript clusters with differential expression were investigated for functional interrelatedness and networks, excluding microRNA**–**mRNA interactions, using the Ingenuity Pathway Analysis (IPA) program (Ingenuity Systems, Mountain View, CA, USA, version 01–00). All microarray data were deposited in the GEO database (accession numbers GSE80242, GSE80244, GSE80245).

### Statistical analysis

Statistical analysis of microarray data was performed using Transcriptome Analysis Console software. Gene-level estimates from mouse microarray data were subjected to one-way ANOVA, and the obtained transcript clusters with *p* values < 0.05 were subjected to a specific comparison (ADh/hTFAMh vs. ADh/WT), with a fold-change >1.6 as a threshold for the comparison. Gene-level estimates from human AD microarray data were subjected to one-way ANOVA, and the obtained transcript clusters with *p* values < 0.05 were subjected to specific comparisons of PS1_P117L_ versus wild-type or PS1_P117L_ + rhTFAM versus PS1_P117L_, with a fold-change >12 or 8 as a threshold for the comparison. Statistical analyses of other experiments were performed using JMP Pro 11.0.0 software (SAS Institute Japan Ltd., Tokyo, Japan). The statistical significance between groups was determined using parametric and nonparametric methods according to data distribution, as described. P values < 0.05 were considered statistically significant.

## Additional Information

**How to cite this article**: Oka, S. *et al*. Human mitochondrial transcriptional factor A breaks the mitochondria-mediated vicious cycle in Alzheimer’s disease. *Sci. Rep.* **6**, 37889; doi: 10.1038/srep37889 (2016).

**Publisher's note:** Springer Nature remains neutral with regard to jurisdictional claims in published maps and institutional affiliations.

## Supplementary Material

Supplementary Information
