# Supplementary material for: Human mitochondrial transcriptional factor A breaks the mitochondria-mediated vicious cycle in Alzheimer’s disease
Source: Sci Rep. 2016 Nov 29;6:37889. doi: 10.1038/srep37889 (PMC5126576; doi:10.1038/srep37889)
Supplement: Supplementary Information [file srep37889-s1.pdf]

Supplementary information for:

Human mitochondrial transcriptional factor A breaks the mitochondria-mediated vicious cycle in Alzheimer's disease

Sugako Oka[1], Julio Leon[1], Kunihiko Sakumi[1], Tomomi Ide[2], Dongchon Kang[3], Frank M. LaFerla[4], and Yusaku Nakabeppu[1]

[1]Division of Neurofunctional Genomics, Department of Immunobiology and Neuroscience, Medical Institute of Bioregulation, [2]Department of Cardiovascular Medicine, and [3]Department of Clinical Chemistry and Laboratory Medicine, Graduate School of Medical Sciences, Kyushu University, 3-1-1 Maidashi, Higashi-Ku, Fukuoka 812-8582, Japan
[4]Department of Neurobiology and Behavior, University of California, Irvine, CA 92697, USA

This file contains:

Supplementary Tables S1 – S8
Supplementary Figures S1 –S9
Supplementary Methods
Supplementary References

Supplementary Table S1. List of transcript clusteres which exhibit significantly increased expression in ADh/hTFAMh hippocampus.

Transcript cluster ID	Gene symbol	p-value (ADh/hTFAMh vs ADh/WT)	Fold change** (ADh/hTFAMh vs ADh/WT)	Raw expression level (log2)*		Standard Deviation (log2)		Gene description
				ADh/hTFAMh	ADh/WT	ADh/hTFAMh	ADh/WT	
10356403	Kcnj13	0.0164	14.89	11.06	7.16	1.44	0.68	potassium inwardly-rectifying channel, subfamily J, member 13
10454192	Ttr	0.0050	8.74	13.63	10.51	0.70	0.53	transthyretin
10569344	Igf2	0.0159	3.51	10.52	8.71	0.60	0.33	insulin-like growth factor 2
10438769	Cldn1	0.0307	3.14	8.47	6.82	0.88	0.09	claudin 1
10543921	Slc13a4	0.0166	3.08	9.10	7.48	0.57	0.41	solute carrier family 13 (sodium/sulfate symporters), member 4
10440091	Col8a1	0.0396	2.72	8.06	6.61	0.78	0.20	collagen, type VIII, alpha 1
10586865	Aldh1a2	0.0108	2.58	8.07	6.71	0.32	0.27	aldehyde dehydrogenase family 1, subfamily A2
10412207	Gpx8	0.0461	2.57	9.07	7.71	0.74	0.28	glutathione peroxidase 8 (putative)
10597960	Slc6a20a	0.0166	2.44	9.95	8.66	0.05	0.45	solute carrier family 6 (neurotransmitter transporter), member 20A
10534862	Pcolce	0.0301	2.44	8.90	7.62	0.53	0.39	procollagen C-endopeptidase enhancer protein
10422728	Dab2	0.0188	2.35	9.14	7.91	0.43	0.21	disabled 2, mitogen-responsive phosphoprotein
10347277	Igfbp2	0.0493	2.23	8.78	7.62	0.74	0.08	insulin-like growth factor binding protein 2
10467153	Slc16a12	0.0179	1.88	7.32	6.41	0.38	0.11	solute carrier family 16 (monocarboxylic acid transporters), member 12
10405063	Ogn	0.0354	1.83	8.10	7.23	0.12	0.36	osteoglycin
10484463	Serping1	0.0196	1.80	8.86	8.01	0.09	0.32	serine (or cysteine) peptidase inhibitor, clade G, member 1
10351131	Myoc	0.0223	1.78	7.52	6.68	0.29	0.07	myocilin
10535174	Tmem184a	0.0330	1.73	7.36	6.57	0.42	0.07	transmembrane protein 184a
10461115	Slc22a8	0.0150	1.70	9.08	8.31	0.01	0.26	solute carrier family 22 (organic anion transporter), member 8
10548879	Mgp	0.0154	1.67	7.79	7.05	0.12	0.24	matrix Gla protein
10490129	Bmp7	0.0160	1.63	8.76	8.05	0.22	0.20	bone morphogenetic protein 7

*Average raw expression level (log2) in ADh/hTFAMh >6.6. **Fold change >1.6 with p <0.05 (unpaired t-test).

Gene symbols in red indicate Alzheimer's disease-related genes.

Supplementary Table S2. List of transcript clusteres which exhibit significantly decreased expression in ADh/hTFAMh hippocampus.

Transcript cluster ID	Gene symbol	p-value (ADh/hTFAMh vs ADh/WT)	Fold change** (ADh/hTFAMh vs ADh/WT)	Raw expression level (log2)*		Standard Deviation (log2)		Gene description
				ADh/hTFAMh	ADh/WT	ADh/hTFAMh	ADh/WT	
10582916	Gm17535	0.0323	-3.95	8.81	10.79	0.86	0.40	predicted gene 17535
10356269	LOC100041708	0.0168	-3.19	6.69	8.37	0.66	0.33	nuclear body protein SP140-like; uncharacterized LOC102634585; RIKEN cDNA A530032D15Rik gene; Sp140 nuclear body protein; predicted gene 2427
10582896	Gm10718	0.0307	-3.16	9.26	10.92	0.73	0.42	predicted genes 10718; 11168
10582888	Gm10719	0.0328	-3.14	10.07	11.72	0.81	0.32	predicted genes 10719; 10721
10582884	Gm11168	0.0423	-2.77	5.99	7.46	0.70	0.33	predicted gene 11168
10490203	Gm20721	0.0183	-2.65	8.10	9.51	0.26	0.53	predicted gene 20721
10526838	Gm10874	0.0294	-2.10	7.51	8.58	0.15	0.53	predicted gene 10874
10582862	Sp140	0.0170	-2.07	5.63	6.68	0.20	0.32	Sp140 nuclear body protein; predicted gene 2427; nuclear body protein SP140-like; nuclear body protein SP140-like protein-like; uncharacterized LOC102633858; predicted genes 7281; 6264
10582899	Gm10717	0.0423	-2.01	8.89	9.90	0.48	0.24	predicted genes 10717; 10715
10608368	Gm20806	0.0164	-1.99	5.85	6.84	0.35	0.30	Y-linked testis-specific protein 1-like; spermiogenesis specific transcript on the Y 2; spermiogenesis specific transcript on the Y 2-like; predicted genes 20806; 20738; 21784; 21316; 21249; 20924; 20823; 2184521672; 21308; 21180; 20867; 20816; 20803; 21350; 21890; 21683; 20909; 20801; 20747; 20925; 21340; 21302; 20852; 21469; 20917; 21242; 21065; 21661; 21462; 21443; 21427; 21412; 21344; 21201; 21155; 21151; 21076; 20932; 20927; 20919; 20863; 20861; 20846; 20809; 20808; 20791; 20865; 21791; 21118; 21642; 21617; 21506; 21470; 21396; 21245; 21127; 20921; 20907; 20902; 20892; 20891; 20887; 20881; 20859; 20856; 20842; 20841; 20805; 20804; 20799; 20926; 21943; 20815; 21292; 21573; 21330; 21281; 21247; 21184; 21163; 20818; 20825; 21244; 20826; 20854; 21763; 21275; 20934; 20898; 20848; 20847; 20840; 20879; 21812; 21721; 21728; 21920; 21889; 20914; 20822; 20877; 20830; 20812; 20828; 21301; 20834; 20918; 20821
10608295	Gm21943	0.0133	-1.96	6.03	7.00	0.32	0.25	Y-linked testis-specific protein 1-like; spermiogenesis specific transcript on the Y 2; spermiogenesis specific transcript on the Y 2-like; predicted genes 21943; 20815; 20747; 20806; 20738; 21784; 21316; 21249; 20924; 20823; 21845; 21672; 21308; 21180; 20867; 20816; 20803; 21350; 21642; 21617; 21506; 21470; 21396; 21245; 21127; 20921; 20907; 20902; 20892; 20891; 20887; 20881; 20859; 20856; 20842; 20841; 20805; 20804; 20799; 21573; 21330; 21281; 21247; 21184; 21163; 20818; 21890; 21683; 20909; 20801; 20825; 20925; 21340; 21302; 20852; 20854; 21469; 20917; 21242; 21065; 21661; 21462; 21443; 21427; 21412; 21344; 21201; 21155; 21151; 21076; 20932; 20927; 20919; 20863; 20861; 20846; 20809; 20808; 20791; 20865; 21275; 20934; 20898; 20848; 20847; 20840; 21812; 21791; 21728; 21118; 21920; 21889; 20926; 21292; 21244; 20826; 20877; 20812; 21763; 20879; 21721; 20918; 20821; 20914; 20830; 21301; 20834; 20781; 21736
10528810	Gm10471	0.0122	-1.95	5.86	6.83	0.39	0.03	spermatogenesis associated glutamate (E)-rich protein 4a; RIKEN cDNA 5031410I06 gene; spermatogenesis associated glutamate (E)-rich protein 4b; spermatogenesis associated glutamate (E)-rich protein 4e; predicted genes 10471; 1979; 21680; 10220; 7361; 7347; 21698; 21663; 5862; 21671; 21190;
10608377	Gm20747	0.0167	-1.91	5.92	6.85	0.39	0.28	spermiogenesis specific transcript on the Y 2; spermiogenesis specific transcript on the Y 2-like; Y-linked testis-specific protein 1-like; predicted genes 20747; 20806; 20738; 21784; 21316; 21249; 20924; 20823; 21845; 21672; 21308; 21180; 20867; 20816; 20803; 21350; 21890; 21683; 20909; 20801; 20825; 20925; 21340; 21302; 20852; 21469; 20917; 21242; 21065; 21661; 21462; 21443; 21427; 21412; 21344; 21201; 21155; 21151; 21076; 20932; 20927; 20919; 20863; 20861; 20846; 20809; 20808; 20791; 20865; 21275; 20934; 20898; 20848; 20847; 20840; 21812; 21791; 21118; 21920; 21889; 21642; 21617; 21506; 21470; 21396; 21245; 21127; 20921; 20907; 20902; 20892; 20891; 20887; 20881; 20859; 20856; 20842; 20841; 20805; 20804; 20799; 20926; 21943; 20815; 21292; 21573; 21330; 21281; 21247; 21184; 21163; 20818; 21244; 20826; 20854; 20879; 21721; 20918; 21728; 20821; 20914;; 20822; 20877; 20830; 20812; 20828; 21301; 20834; 21763; 20781; 21736
10608480	LOC102639563	0.0086	-1.91	6.61	7.54	0.31	0.05	uncharacterized LOC102639563; uncharacterized LOC102633922; Y-linked testis-specific protein 1-like; spermiogenesis specific transcript on the Y 1; predicted genes 21719; 20831; 21678; 21440; 21425; 20928; 20851; 20833; 20827; 20807; 20793; 20777; 20773; 20772; 20737; 21871; 21660; 20836; 20795; 21907; 21454; 20910; 21310; 21768

Supplementary Table S2. (continued)

Transcript cluster ID	Gene symbol	p-value (ADh/hTFAMh vs ADh/WT)	Fold change** (ADh/hTFAMh vs ADh/WT)	Raw expression level (log2)*		Standard Deviation (log2)		Gene description
				ADh/hTFAMh	ADh/WT	ADh/hTFAMh	ADh/WT	
10580056	Gm10644	0.0264	-1.85	7.77	8.66	0.23	0.30	predicted gene 10644
10608606	Gm20815	0.0177	-1.84	5.87	6.75	0.36	0.29	Y-linked testis-specific protein 1-like; spermiogenesis specific transcript on the Y 2; spermiogenesis specific transcript on the Y 2-like; predicted genes 20815; 20747; 20806; 20738; 21784; 21316; 21249; 20924; 20823; 21845; 21672; 21308; 21180; 20867; 20816; 20803; 21350; 21890; 21683; 20909; 20801; 20825; 20925; 21340; 21302; 20852; 21469; 20917; 21242; 21065; 21661; 21462; 21443; 21427; 21412; 21344; 21201; 21155; 21151; 21076; 20932; 20927; 20919; 20863; 20861; 20846; 20809; 20808; 20791; 20865; 21275; 20934; 20898; 20848; 20847; 20840; 21791; 21118; 21920; 21889; 21642; 21617; 21506; 21470; 21396; 21245; 21127; 20921; 20907; 20902; 20892; 20891; 20887; 20881; 20859; 20856; 20842; 20841; 20805; 20804; 20799; 20926; 21943; 21292; 21573; 21330; 21281; 21247; 21184; 21163; 20818; 21244; 20826; 20854; 20879; 21812; 21721; 20918; 21728; 20821; 20914; 20822; 20877; 20830; 20812; 20828; 21301; 20834; 21763
10564057	Gm22252	0.0247	-1.81	8.40	9.25	0.22	0.39	predicted gene 22252
10608531	Gm21882	0.0177	-1.75	6.24	7.05	0.16	0.28	RIKEN cDNA 4932431L22 gene; serine-rich, secreted, X-linked; serine-rich, secreted, Y-linked; predicted genes 21882; 21820; 21776; 21732; 21773; 21913; 21921
10572130	Lpl	0.0004	-1.74	8.34	9.14	0.09	0.08	lipoprotein lipase
10605067	Pnck	0.0267	-1.69	9.76	10.52	0.05	0.32	pregnancy upregulated non-ubiquitously expressed CaM kinase
10607792	Glra2	0.0137	-1.67	6.83	7.57	0.14	0.29	glycine receptor, alpha 2 subunit
10528821	Gm10220	0.0084	-1.67	6.30	7.04	0.25	0.05	RIKEN cDNA 5031410I06 gene; spermatogenesis associated glutamate (E)-rich protein 4a; spermatogenesis associated glutamate (E)-rich protein 4b; spermatogenesis associated glutamate (E)-rich protein 4e; 10220; 21698; 21663; 10471; 1979; 7361; 7347; 5862; 21680; 17019; 21671; 21655; 21190
10563961	Gm23145	0.0379	-1.66	6.87	7.61	0.20	0.44	predicted gene, 23145
10351041	Gm24489	0.0270	-1.65	7.71	8.43	0.08	0.30	predicted gene, 24489; growth arrest specific 5
10528191	Speer4c	0.0027	-1.65	6.03	6.76	0.19	0.08	spermatogenesis associated glutamate (E)-rich protein 4c; spermatogenesis associated glutamate (E)-rich protein 4e; spermatogenesis associated glutamate (E)-rich protein 4d; spermatogenesis associated glutamate (E)-rich protein pseudogenespermatogenesis associated glutamate (E)-rich protein 4a; spermatogenesis associated glutamate (E)-rich protein 4b; RIKEN cDNA 4930572O03 gene; RIKEN cDNA 5031410I06 gene; predicted genes 10354; 17019; 9758; 21083; 21190; 21149; 10471; 7347; 21655; 10220;1979; 21698; 21663, 21680; 5862
10603228	Gm14369	0.0221	-1.61	6.66	7.34	0.30	0.24	predicted genes 14369; 14333; 15275; 21883; 21789; 21870; 14355; 14362; 15281; 14364; 15254; 21990

*Average raw expression level (log2) in ADh/WT >6.6. **Fold change <-1.6 with p <0.05 (unpaired t-test).
A gene symbol in red indicates Alzheimer's disease-related gene.

Supplementary Table S3. List of transcript clusteres which exhibit significantly decreased expression in PS1$_{P117L}$ cells.

Transcript cluster ID	Gene symbol	p-value (PS1$_{P117L}$ vs Wild)	Fold change** (PS1$_{P117L}$ vs Wild type)	Raw expression level (log2)*		Standard Deviation (log2)		Gene description
				PS1$_{P117L}$	Wild type	PS1$_{P117L}$	Wild type	
7981964	SNORD116-8	0.0079	-119.02	4.15	11.05	1.9	0.79	small nucleolar RNA, C/D box 116-8; small nucleolar RNA, C/D box 116-3; small nucleolar RNA, C/D box 116-9; small nucleolar RNA, C/D box 116-6
7981996	SNORD116-24	0.0188	-105.82	4.66	11.39	1.61	1.75	small nucleolar RNA, C/D box 116-24
7938329	SNORA23	0.0115	-83.38	4.59	10.97	1.89	0.85	small nucleolar RNA, H/ACA box 23
7981953	SNORD116-3	0.0117	-79.96	5.08	11.4	2.01	0.87	small nucleolar RNA, C/D box 116-3; small nucleolar RNA, C/D box 116-9; small nucleolar RNA, C/D box 116-5; small nucleolar RNA, C/D box 116-7; small nucleolar RNA, C/D box 116-8
7981978	SNORD116-15	0.0247	-75.92	6.66	12.91	1.81	1.31	small nucleolar RNA, C/D box 116-15
7981982	SNORD116-19	0.0315	-56.56	6.23	12.06	1.7	1.36	small nucleolar RNA, C/D box 116-19; small nucleolar RNA, C/D box 116-17; small nucleolar RNA, C/D box 116-20; small nucleolar RNA, C/D box 116-21; small nucleolar RNA, C/D box 116 cluster; small nuclear ribonucleoprotein polypeptide N; small nucleolar RNA, C/D box 116-15; small nucleolar RNA, C/D box 116-16; small nucleolar RNA, C/D box 116-18; small nucleolar RNA, C/D box 116-22; imprinted in Prader-Willi syndrome (non-protein coding); small nucleolar RNA, C/D box 107; Prader Willi/Angelman region RNA, SNRPN neighbor; small nucleolar RNA, C/D box 116-4; small nucleolar RNA, C/D box 115-7; small nucleolar RNA, C/D box 115-13; small nucleolar RNA, C/D box 115-26; small nucleolar RNA, C/D box 116-28; uncharacterized LOC101930404; small nucleolar RNA, C/D box 116-14
8175531	CDR1	0.0144	-52.81	4.89	10.61	1.86	0.68	cerebellar degeneration-related protein 1, 34kDa
7981958	SNORD116-5	0.0080	-51.44	4.51	10.2	1.62	1.09	small nucleolar RNA, C/D box 116-5; small nucleolar RNA, C/D box 116-7; small nucleolar RNA, C/D box 116-3; small nucleolar RNA, C/D box 116-9
7981988	SNORD116-20	0.0185	-43.46	6	11.44	1.38	1.59	small nucleolar RNA, C/D box 116-20; small nucleolar RNA, C/D box 116-19; small nucleolar RNA, C/D box 116-17; small nucleolar RNA, C/D box 116-21; small nucleolar RNA, C/D box 116 cluster
7981976	SNORD116-14	0.0461	-42.25	6.08	11.48	1.86	1.91	small nucleolar RNA, C/D box 116-14; small nuclear ribonucleoprotein polypeptide N
8047926	MAP2	0.0065	-40.09	4.41	9.74	1.31	0.75	microtubule-associated protein 2
8077366	LRRN1	0.0089	-39.20	3.66	8.95	0.82	1.25	leucine rich repeat neuronal 1
7925525	CEP170	0.0073	-38.81	3.69	8.97	0.83	1.29	centrosomal protein 170kDa; centrosomal protein 170kDa pseudogene 1
8154305	SELT	0.0246	-37.24	3.35	8.57	1.96	0.45	selenoprotein T
7897801	RNU5E-1	0.0127	-32.77	6.24	11.28	1.04	1.45	RNA, U5E small nuclear 1
7930208	INA	0.0007	-30.91	5.29	10.24	0.67	0.63	internexin neuronal intermediate filament protein, alpha
8147030	STMN2	0.0005	-29.69	5.56	10.45	0.19	0.82	stathmin 2
7981960	SNORD116-6	0.0032	-29.11	3.77	8.64	0.73	1.06	small nucleolar RNA, C/D box 116-6
8019930	MYL12B	0.0260	-27.90	3.98	8.79	1.87	0.49	myosin, light chain 12B, regulatory
7899480	SNORA73A	0.0473	-26.67	5.69	10.43	1.93	1.22	small nucleolar RNA, H/ACA box 73A; small nucleolar RNA host gene 3 (non-protein coding)
7981998	SNORD116-25	0.0041	-26.06	3.64	8.35	0.38	1.3	small nucleolar RNA, C/D box 116-25
7981994	SNORD116-23	0.0070	-25.22	4.16	8.81	0.97	0.93	small nucleolar RNA, C/D box 116-23
8135544	FOXP2	0.0073	-25.04	3.13	7.78	0.71	1.03	forkhead box P2
8062395	NNAT	0.0044	-24.60	5.98	10.6	1.02	0.63	neuronatin
8049961	FBXO25	0.0040	-23.70	2.28	6.85	0.44	1.11	F-box protein 25; uncharacterized LOC728323
7981955	SNORD116-4	0.0157	-23.70	4.35	8.91	0.97	1.42	small nucleolar RNA, C/D box 116-4; imprinted in Prader-Willi syndrome (non-protein coding); small nucleolar RNA, C/D box 107; Prader Willi/Angelman region RNA, SNRPN neighbor; small nucleolar RNA, C/D box 116-22; small nucleolar RNA, C/D box 115-7; small nucleolar RNA, C/D box 115-13; small nucleolar RNA, C/D box 115-26; small nucleolar RNA, C/D box 116-28; uncharacterized LOC101930404; small nuclear ribonucleoprotein polypeptide N
8012110	GABARAP	0.0222	-23.52	5.59	10.15	1.53	0.52	GABA(A) receptor-associated protein
7954104	ATF7IP	0.0111	-23.44	4.07	8.62	0.9	1.01	activating transcription factor 7 interacting protein
7981949	SNORD116-1	0.0074	-22.95	4.52	9.05	1.22	1	small nucleolar RNA, C/D box 116-1
8086752	SNORD13P3	0.0308	-22.63	5.54	10.04	1.57	1.25	small nucleolar RNA, C/D box 13 pseudogene 3
8000217	SMG1	0.0054	-22.52	5.14	9.64	0.91	0.84	SMG1 phosphatidylinositol 3-kinase-related kinase; SMG1 pseudogene 5; SMG1 pseudogene 2; SMG1 pseudogene 1; SMG1 pseudogene 3; serine/threonine-protein kinase SMG1-like; bolA family member 2; solute carrier family 7 (amino acid transporter light chain, L system), member 5 pseudogene 1; putative L-type amino acid transporter 1-like protein IMAA-like; SMG1 pseudogene 7
8157890	PBX3	0.0009	-22.35	5.22	9.7	0.31	0.73	pre-B-cell leukemia homeobox 3
7981990	SNORD116-21	0.0077	-22.17	3.43	7.9	1.32	0.19	small nucleolar RNA, C/D box 116-21; small nucleolar RNA, C/D box 116-19; small nucleolar RNA, C/D box 116-17; small nucleolar RNA, C/D box 116-20; small nucleolar RNA, C/D box 116 cluster
7982084	SNORD115-1	0.0083	-22.06	5.34	9.8	1.23	0.84	small nucleolar RNA, C/D box 115-1; small nucleolar RNA, C/D box 115-5; small nucleolar RNA, C/D box 115-9; small nucleolar RNA, C/D box 115-11; small nucleolar RNA, C/D box 115-12; small nucleolar RNA, C/D box 115-13; small nucleolar RNA, C/D box 115-16; small nucleolar RNA, C/D box 115-22; small nucleolar RNA, C/D box 115-26; small nucleolar RNA, C/D box 115-29; small nucleolar RNA, C/D box 115-36; small nucleolar RNA, C/D box 115-39; small nucleolar RNA, C/D box 115-40; small nucleolar RNA, C/D box 115-41; small nucleolar RNA, C/D box 115-43; imprinted in Prader-Willi syndrome (non-protein coding); small nucleolar RNA, C/D box 107; Prader Willi/Angelman region RNA, SNRPN neighbor; small nucleolar RNA, C/D box 116-4; small nucleolar RNA, C/D box 116-22; small nucleolar RNA, C/D box 115-7; small nucleolar RNA, C/D box 116-28; uncharacterized LOC101930404; small nuclear ribonucleoprotein polypeptide N; small nucleolar RNA, C/D box 115-4; small nucleolar RNA, C/D box 115-6; small nucleolar RNA, C/D box 115-8; small nucleolar RNA, C/D box 115-10; small nucleolar RNA, C/D box 115-14; small nucleolar RNA, C/D box 115-15; small nucleolar RNA, C/D box 115-20; small nucleolar RNA, C/D box 115-21; small nucleolar RNA, C/D box 115-34; small nucleolar RNA, C/D box 115-38; small nucleolar RNA, C/D box 115-42; small nucleolar RNA, C/D box 115-44; small nucleolar RNA, C/D box 115-24; small nucleolar RNA, C/D box 115-3; small nucleolar RNA, C/D box 115-17; small nucleolar RNA, C/D box 115-18; small nucleolar RNA, C/D box 115-19; small nucleolar RNA, C/D box 115-25; small nucleolar RNA, C/D box 115-30
8146216	VDAC3	0.0198	-22.05	4.07	8.53	1.39	0.77	voltage-dependent anion channel 3
8134339	PEG10	0.0143	-21.77	6.52	10.97	1.34	0.76	paternally expressed 10
7941272	MALAT1	0.0116	-21.75	6.78	11.22	1.44	0.62	metastasis associated lung adenocarcinoma transcript 1 (non-protein coding)
8055476	YWHAE	0.0021	-21.63	5.61	10.04	0.97	0.48	tyrosine 3-monooxygenase/tryptophan 5-monooxygenase activation protein, epsilon; tyrosine 3-monooxygenase/tryptophan 5-monooxygenase activation protein, epsilon pseudogene 5
8101212	CCNI	0.0090	-21.38	5.55	9.97	1.21	0.74	cyclin I
8060503	SNORD57	0.0026	-21.28	5.39	9.8	0.66	0.79	small nucleolar RNA, C/D box 57; NOP56 ribonucleoprotein; small nucleolar RNA, C/D box 86; small nucleolar RNA, C/D box 110; microRNA 1292
8007228	ATP6V0A1	0.0051	-21.07	4.14	8.53	0.79	0.93	ATPase, H+ transporting, lysosomal V0 subunit a1
7903092	FNBP1L	0.0048	-20.80	4.3	8.68	0.69	1.01	formin binding protein 1-like
8112940	SSBP2	0.0080	-20.78	5.25	9.63	0.77	0.92	single-stranded DNA binding protein 2
8174543	DCX	0.0047	-20.49	5.89	10.24	0.87	0.99	doublecortin
8177858	TUBB	0.0151	-20.25	6.13	10.47	1.3	0.86	tubulin, beta class I; tubulin, beta pseudogene 1
8135277	KMT2E	0.0042	-19.92	4.2	8.51	0.15	1.15	lysine (K)-specific methyltransferase 2E
8113073	ARRDC3	0.0058	-19.71	4.53	8.83	0.29	1.14	arrestin domain containing 3
8074925	GUSBP2	0.0099	-19.55	4.83	9.12	0.95	0.96	glucuronidase, beta pseudogene 2; glucuronidase, beta pseudogene; glucuronidase, beta pseudogene 11; glucuronidase, beta pseudogene 1; glucuronidase, beta pseudogene 4; beta-glucuronidase-like protein SMA4-like; glucuronidase, beta pseudogene 9; glucuronidase, beta; uncharacterized LOC102725134
8094778	UCHL1	0.0178	-19.07	4.81	9.07	1.45	0.5	ubiquitin carboxyl-terminal esterase L1 (ubiquitin thiolesterase)
7965467	RPL41	0.0356	-19.06	6.14	10.39	1.85	0.17	ribosomal protein L41; ribosomal protein L41 pseudogene 1; ribosomal protein L41 pseudogene 2
7975989	SLIRP	0.0288	-18.91	4.45	8.69	1.63	0.36	SRA stem-loop interacting RNA binding protein
8052269	CCDC88A	0.0119	-18.83	3.93	8.16	0.09	1.38	coiled-coil domain containing 88A
7913869	STMN1	0.0013	-18.80	4.69	8.93	0.6	0.5	stathmin 1
8169389	PAK3	0.0013	-18.74	4.53	8.76	0.39	0.84	p21 protein (Cdc42/Rac)-activated kinase 3
8026875	SNORA68	0.0023	-18.71	5.43	9.66	0.39	1	small nucleolar RNA, H/ACA box 68; ribosomal protein L18a
8086077	CLASP2	0.0030	-18.59	4.58	8.8	0.16	1.03	cytoplasmic linker associated protein 2
8091446	PFN2	0.0062	-18.51	4.43	8.64	0.91	0.87	profilin 2
8110522	CANX	0.0254	-18.24	5.8	9.99	1.45	0.86	calnexin
7947274	MPPED2	0.0022	-18.21	5.12	9.3	0.37	0.85	metallophosphoesterase domain containing 2
8098195	MSMO1	0.0081	-18.12	4.08	8.26	1.05	0.83	methylsterol monooxygenase 1
8052010	CALM1	0.0262	-17.95	4.11	8.28	1.55	0.86	calmodulin 1 (phosphorylase kinase, delta); calmodulin 2 (phosphorylase kinase, delta); calmodulin 3 (phosphorylase kinase, delta)
7963054	TUBA1A	0.0125	-17.71	5.7	9.85	1.3	0.41	tubulin, alpha 1a
7948894	RNU2-2P	0.0338	-17.68	5.73	9.87	1.13	1.49	RNA, U2 small nuclear 2, pseudogene; RNA, U2 small nuclear 1; WD repeat domain 74; RNA, U2 small nuclear 36, pseudogene

Supplementary Table S3. (continued)

Transcript cluster ID	Gene symbol	p-value ($PS1_{P117L}$ vs Wild)	Fold change** ($PS1_{P117L}$ vs Wild type)	Raw expression level (log2)*		Standard Deviation (log2)		Gene description
				$PS1_{P117L}$	Wild type	$PS1_{P117L}$	Wild type	
8166784	TSPAN7	0.0140	-17.48	5.13	9.26	1.01	0.92	tetraspanin 7
7979864	ERH	0.0130	-17.27	5	9.11	1.18	0.69	enhancer of rudimentary homolog (Drosophila)
7982094	SNORD115-44	0.0177	-16.93	3	7.08	1.04	1.17	small nucleolar RNA, C/D box 115-44
8133106	SNORA22	0.0035	-16.81	5.1	9.17	0.28	1.12	small nucleolar RNA, H/ACA box 22
7943160	SCARNA9	0.0005	-16.80	3.18	7.25	0.5	0.54	small Cajal body-specific RNA 9; small Cajal body-specific RNA 9-like
8056909	ATF2	0.0041	-16.61	4.43	8.48	0.23	1	activating transcription factor 2
7965048	NAP1L1	0.0017	-16.47	4.1	8.14	0.52	0.62	nucleosome assembly protein 1-like 1
7969533	SLAIN1	0.0055	-16.32	4.57	8.6	0.5	0.97	SLAIN motif family, member 1
7957338	SYT1	0.0023	-16.11	5.33	9.34	0.53	0.9	synaptotagmin I
7944656	SC5D	0.0085	-16.03	4.13	8.14	0.22	1.31	sterol-C5-desaturase
8021245	DCC	0.0182	-15.82	4.85	8.83	0.88	1.19	DCC netrin 1 receptor
8073548	SEPT3	0.0023	-15.70	5.49	9.46	0.49	0.76	septin 3
8109697	CCNG1	0.0039	-15.63	4.46	8.42	0.18	0.96	cyclin G1
8053834	ANKRD36C	0.0425	-15.57	4.21	8.17	1.18	1.54	ankyrin repeat domain 36C; ankyrin repeat domain 36; ankyrin repeat domain 36B
8021376	NEDD4L	0.0049	-15.54	4.35	8.31	0.22	1.05	neural precursor cell expressed, developmentally down-regulated 4-like, E3 ubiquitin protein ligase
7976598	PAPOLA	0.0330	-15.52	4.21	8.16	1.21	1.2	poly(A) polymerase alpha
8062880	YWHAB	0.0040	-15.25	4.76	8.69	0.32	1.04	tyrosine 3-monooxygenase/tryptophan 5-monooxygenase activation protein, beta
8106743	VCAN	0.0051	-15.19	4.3	8.23	0.23	1.13	versican
7918825	CSDE1	0.0221	-15.18	5.18	9.1	1.54	0.96	cold shock domain containing E1, RNA-binding
8152096	YWHAZ	0.0102	-15.12	5.57	9.49	0.91	0.9	tyrosine 3-monooxygenase/tryptophan 5-monooxygenase activation protein, zeta; tyrosine 3-monooxygenase/tryptophan 5-monooxygenase activation protein, zeta pseudogene 3
8145793	SNORD13	0.0141	-15.06	6.45	10.37	1.18	1.13	small nucleolar RNA, C/D box 13
8091778	SCARNA7	0.0000	-15.01	4.12	8.02	0.14	0.33	small Cajal body-specific RNA 7
7946610	EIF4G2	0.0010	-14.96	5.36	9.26	0.19	0.75	eukaryotic translation initiation factor 4 gamma, 2
8047538	BMPR2	0.0137	-14.80	4.56	8.44	0.46	1.38	bone morphogenetic protein receptor, type II (serine/threonine kinase)
7982000	SNORD116-26	0.0161	-14.64	5.2	9.07	0.88	1.42	small nucleolar RNA, C/D box 116-26
8133688	SNORA14A	0.0078	-14.58	4.39	8.25	0.44	1.05	small nucleolar RNA, H/ACA box 14A
7905329	MLLT11	0.0096	-14.55	7.04	10.9	1.01	0.69	myeloid/lymphoid or mixed-lineage leukemia (trithorax homolog, Drosophila); translocated to, 11
7982058	SNORD115-5	0.0048	-14.53	5.31	9.17	0.91	0.75	small nucleolar RNA, C/D box 115-5; small nucleolar RNA, C/D box 115-9; small nucleolar RNA, C/D box 115-11; small nucleolar RNA, C/D box 115-12; small nucleolar RNA, C/D box 115-22; small nucleolar RNA, C/D box 115-26; small nucleolar RNA, C/D box 115-29; small nucleolar RNA, C/D box 115-36; small nucleolar RNA, C/D box 115-43; imprinted in Prader-Willi syndrome (non-protein coding); small nucleolar RNA, C/D box 107; Prader Willi/Angelman region RNA, SNRPN neighbor; small nucleolar RNA, C/D box 116-4; small nucleolar RNA, C/D box 116-22; small nucleolar RNA, C/D box 115-7; small nucleolar RNA, C/D box 115-13; small nucleolar RNA, C/D box 116-28; uncharacterized LOC101930404; small nuclear ribonucleoprotein polypeptide N; small nucleolar RNA, C/D box 115-1; small nucleolar RNA, C/D box 115-3; small nucleolar RNA, C/D box 115-15; small nucleolar RNA, C/D box 115-16; small nucleolar RNA, C/D box 115-17; small nucleolar RNA, C/D box 115-18; small nucleolar RNA, C/D box 115-19; small nucleolar RNA, C/D box 115-20; small nucleolar RNA, C/D box 115-21; small nucleolar RNA, C/D box 115-25; small nucleolar RNA, C/D box 115-39; small nucleolar RNA, C/D box 115-40; small nucleolar RNA, C/D box 115-41; small nucleolar RNA, C/D box 115-44; small nucleolar RNA, C/D box 115-4; small nucleolar RNA, C/D box 115-6; small nucleolar RNA, C/D box 115-8; small nucleolar RNA, C/D box 115-10; small nucleolar RNA, C/D box 115-14; small nucleolar RNA, C/D box 115-30; small nucleolar RNA, C/D box 115-31; small nucleolar RNA, C/D box 115-34; small nucleolar RNA, C/D box 115-38; small nucleolar RNA, C/D box 115-42; small nucleolar RNA, C/D box 115-24
8099982	APBB2	0.0317	-14.51	4.1	7.96	0.66	1.34	amyloid beta (A4) precursor protein-binding, family B, member 2
8178298	GABBR1	0.0015	-14.42	4.16	8.01	0.29	0.8	gamma-aminobutyric acid (GABA) B receptor, 1
8170891	GDI1	0.0130	-14.36	6.09	9.94	1.27	0.81	GDP dissociation inhibitor 1
7911619	GNB1	0.0038	-14.16	5.86	9.68	0.68	0.71	guanine nucleotide binding protein (G protein), beta polypeptide 1
8130578	SNORA20	0.0094	-14.12	4.82	8.64	0.09	1.34	small nucleolar RNA, H/ACA box 20
8005765	WSB1	0.0031	-14.11	4.84	8.65	0.29	1.02	WD repeat and SOCS box containing 1
8106280	HMGCR	0.0060	-14.03	4.35	8.16	0.31	1.01	3-hydroxy-3-methylglutaryl-CoA reductase
8147503	LAPTM4B	0.0018	-14.02	4.85	8.66	0.78	0.42	lysosomal protein transmembrane 4 beta
8052526	XPO1	0.0073	-13.96	3.93	7.74	0.27	1.02	exportin 1
8113504	NREP	0.0005	-13.78	6.16	9.94	0.11	0.61	neuronal regeneration related protein
8160011	KIAA2026	0.0096	-13.75	4.82	8.6	0.34	1.18	KIAA2026
8001197	NETO2	0.0356	-13.69	4.52	8.3	0.36	1.57	neuropilin (NRP) and tolloid (TLL)-like 2
8024436	OAZ1	0.0048	-13.65	5.38	9.15	0.92	0.57	ornithine decarboxylase antizyme 1
7906061	SYT11	0.0117	-13.61	4.41	8.18	0.68	0.9	synaptotagmin XI
8052204	RTN4	0.0169	-13.60	4.28	8.05	1.29	0.85	reticulon 4
8073799	ATXN10	0.0042	-13.59	4.58	8.34	0.71	0.69	ataxin 10
7995739	GNAO1	0.0036	-13.50	5.56	9.32	0.29	0.87	guanine nucleotide binding protein (G protein), alpha activating activity polypeptide O
7984453	PIAS1	0.0077	-13.48	5.39	9.14	0.62	0.99	protein inhibitor of activated STAT, 1
8083233	ZIC1	0.0025	-13.46	5.09	8.84	0.34	0.9	Zic family member 1
8004497	EIF4A1	0.0147	-13.31	4.37	8.11	1.33	0.47	eukaryotic translation initiation factor 4A1; SENP3-EIF4A1 readthrough (NMD candidate); small nucleolar RNA, H/ACA box 67; small nucleolar RNA, H/ACA box 48; small nucleolar RNA, C/D box 10; uncharacterized LOC101928634
8046306	GORASP2	0.0363	-13.28	4.13	7.87	1.11	0.93	golgi reassembly stacking protein 2, 55kDa
7963750	CBX5	0.0080	-13.21	4.88	8.6	0.47	1.2	chromobox homolog 5
7946815	PIK3C2A	0.0246	-13.12	4.11	7.83	0.05	1.43	phosphatidylinositol-4-phosphate 3-kinase, catalytic subunit type 2 alpha
8156905	SANTD3-TMEFF	0.0030	-13.09	5.28	8.99	0.63	0.72	MSANTD3-TMEFF1 readthrough; transmembrane protein with EGF-like and two follistatin-like domains 1
8094948	SLAIN2	0.0180	-13.02	4.42	8.12	1.05	0.89	SLAIN motif family, member 2
7947338	PAX6	0.0103	-12.85	4.88	8.56	0.74	0.93	paired box 6
7973850	AKAP6	0.0082	-12.80	4.63	8.31	0.42	1	A kinase (PRKA) anchor protein 6
8179595	GABBR1	0.0014	-12.79	4.26	7.94	0.13	0.79	gamma-aminobutyric acid (GABA) B receptor, 1
8114030	KIF3A	0.0305	-12.70	3.6	7.27	0.49	1.31	kinesin family member 3A
8030362	SNORD33	0.0019	-12.68	6.2	9.87	0.51	0.77	small nucleolar RNA, C/D box 33; ribosomal protein L13a; small nucleolar RNA, C/D box 35A; small nucleolar RNA, C/D box 34; small nucleolar RNA, C/D box 32A
8172158	CASK	0.0057	-12.65	4.88	8.54	0.56	1.12	calcium/calmodulin-dependent serine protein kinase (MAGUK family)
8169617	PGRMC1	0.0012	-12.65	5.21	8.87	0.26	0.72	progesterone receptor membrane component 1
7932911	KIF5B	0.0076	-12.63	5.06	8.72	0.39	1.01	kinesin family member 5B
7958130	HSP90B1	0.0030	-12.58	5.19	8.84	0.24	0.95	heat shock protein 90kDa beta (Grp94), member 1; microRNA 3652
7969796	TM9SF2	0.0091	-12.58	5.03	8.68	0.87	1.25	transmembrane 9 superfamily member 2
7903188	PTBP2	0.0065	-12.55	4.46	8.11	0.33	1.08	polypyrimidine tract binding protein 2
7897561	KIF1B	0.0085	-12.50	4.94	8.59	0.23	1.09	kinesin family member 1B
8072407	TUG1	0.0024	-12.43	5.27	8.91	0.09	0.9	taurine up-regulated 1 (non-protein coding)
7962537	SLC38A2	0.0306	-12.42	4.14	7.77	0.91	1.24	solute carrier family 38, member 2
8116520	GNB2L1	0.0248	-12.39	6.94	10.57	1.23	0.46	guanine nucleotide binding protein (G protein), beta polypeptide 2-like 1; small nucleolar RNA, C/D box 95; small nucleolar RNA, C/D box 96A
7932554	ARHGAP21	0.0157	-12.37	4.56	8.19	0.5	1.17	Rho GTPase activating protein 21
8104463	MARCH6	0.0049	-12.32	5.16	8.78	0.34	1	membrane-associated ring finger (C3HC4) 6, E3 ubiquitin protein ligase
8158372	SET	0.0040	-12.32	5.03	8.65	0.6	0.74	SET nuclear proto-oncogene; SET-like protein
8037079	ATP1A3	0.0050	-12.31	5.85	9.47	0.8	0.81	ATPase, Na+/K+ transporting, alpha 3 polypeptide; uncharacterized LOC101927137
8016832	MMD	0.0039	-12.30	4.09	7.71	0.56	0.74	monocyte to macrophage differentiation-associated
8155849	ANXA1	0.0064	-12.26	4.86	8.48	0.76	0.96	annexin A1
7920317	ILF2	0.0057	-12.26	4.99	8.61	0.36	0.96	interleukin enhancer binding factor 2
7977775	DAD1	0.0145	-12.23	6.24	9.85	1.13	0.59	defender against cell death 1
8111941	HMGCS1	0.0050	-12.20	4.74	8.35	0.47	0.88	3-hydroxy-3-methylglutaryl-CoA synthase 1 (soluble)
8175076	ZNF280C	0.0408	-12.19	3.86	7.46	0.34	1.53	zinc finger protein 280C
7907310	PRRC2C	0.0076	-12.16	5.23	8.83	0.48	0.9	proline-rich coiled-coil 2C
8046824	FSIP2	0.0151	-12.08	4.17	7.76	0.29	1.36	fibrous sheath interacting protein 2
8106098	MAP1B	0.0077	-12.08	7.13	10.72	0.77	0.8	microtubule-associated protein 1B
7950731	PRCP	0.0336	-12.06	4.01	7.6	0.73	1.16	prolylcarboxypeptidase (angiotensinase C)
7982006	SNORD116-29	0.0010	-12.05	3.74	7.34	0.33	0.69	small nucleolar RNA, C/D box 116-29
8017634	DDX5	0.0086	-12.02	4.73	8.32	0.82	0.85	DEAD (Asp-Glu-Ala-Asp) box helicase 5; microRNA 3064; microRNA 5047
7967900	ZMYM2	0.0077	-12.01	4.6	8.18	0.62	0.95	zinc finger, MYM-type 2

*Average raw expression level (log2) in Wild-type cells >6.6. ** Fold change >12 with p <0.05 (unpaired t-test).
Gene symbols in red indicate Alzheimer's disease-related gene.

Supplementary Table S4. List of genes which were significantly enriched in cellular assembly and organization-related functions among those whose expression is significantly altered in $PS1_{P117L}$ cells.

Functions annotation	p-value	Genes*	# Genes
Organization of cytoplasm	4.51E-06	*ANXA1,ARHGAP21,ATXN10,CANX,CCDC88A,CEP170,CLASP2,DCC,DCX,FNBP1L,GABARAP,GDI1,GNAO1,GORASP2,INA,KIF3A,KIF5B,MAP1B,MAP2,PAK3,PAX6,PFN2,PIK3C2A,RTN4,SLAIN1,SLAIN2,STMN1,STMN2,SYT1,TUBB,UCHL1,XPO1*	32
Organization of cytoskeleton	5.36E-06	*ANXA1,ATXN10,CANX,CCDC88A,CEP170,CLASP2,DCC,DCX,FNBP1L,GABARAP,GDI1,GNAO1,INA,KIF3A,KIF5B,MAP1B,MAP2,PAK3,PAX6,PFN2,PIK3C2A,RTN4,SLAIN1,SLAIN2,STMN1,STMN2,SYT1,TUBB,UCHL1,XPO1*	30
Microtubule dynamics	1.75E-06	*ATXN10,CANX,CCDC88A,CEP170,CLASP2,DCC,DCX,FNBP1L,GABARAP,GDI1,GNAO1,KIF3A,KIF5B,MAP1B,MAP2,PAK3,PAX6,PFN2,PIK3C2A,RTN4,SLAIN1,SLAIN2,STMN1,STMN2,SYT1,TUBB,UCHL1,XPO1*	28
Growth of neurites	5.54E-08	*APBB2,CLASP2,DCC,DCX,EIF4G2,GNAO1,HMGCR,KIF3A,MAP1B,MAP2,NEDD4L,PFN2,RTN4,SET,STMN2,TUBA1A,VCAN,YWHAZ*	18
Formation of cellular protrusions	1.09E-03	*ATXN10,CCDC88A,CLASP2,DCC,DCX,FNBP1L,GDI1,GNAO1,KIF3A,KIF5B,MAP1B,MAP2,PAK3,PAX6,PFN2,RTN4,STMN1,UCHL1*	18
Formation of plasma membrane projections	1.64E-05	*ATXN10,CCDC88A,CLASP2,DCC,DCX,FNBP1L,GDI1,KIF3A,KIF5B,MAP1B,MAP2,PAK3,PAX6,PFN2,RTN4,STMN1,UCHL1*	17
Outgrowth of neurites	2.29E-07	*APBB2,DCC,DCX,EIF4G2,GNAO1,HMGCR,KIF3A,MAP1B,MAP2,PFN2,RTN4,SET,STMN2,TUBA1A,VCAN,YWHAZ*	16
Neuritogenesis	4.71E-05	*ATXN10,CCDC88A,CLASP2,DCC,DCX,FNBP1L,GDI1,KIF3A,MAP1B,MAP2,PAK3,PAX6,PFN2,RTN4,STMN1,UCHL1*	16
Development of cytoplasm	8.30E-06	*ANXA1,CLASP2,DCX,GDI1,GNAO1,GNB1,MAP1B,MAP2,PAK3,PFN2,SLAIN2,SLIRP,STMN1,STMN2,TUBB*	15
Formation of cytoskeleton	1.34E-05	*CLASP2,DCX,GDI1,GNAO1,GNB1,MAP1B,MAP2,PAK3,PFN2,SLAIN2,STMN1,STMN2,TUBB*	13
Formation of filaments	2.61E-05	*CLASP2,DCX,GNAO1,GNB1,INA,MAP1B,MAP2,PAK3,PFN2,SLAIN2,STMN1,STMN2,TUBB*	13
Axonogenesis	9.86E-06	*CLASP2,DCC,GDI1,MAP1B,MAP2,PAK3,PAX6,RTN4,STMN1,UCHL1*	10
Organization of organelle	1.46E-02	*ARHGAP21,CEP170,CLASP2,FNBP1L,GORASP2,INA,MALAT1,PAK3,SLAIN2,TUBB*	10

*Functions in which more than 10 genes are enriched are listed.

Supplementary Table S5. List of genes in common between Group A and B shown in Supplementary Figure S8c.

Gene symbol	Fold change		Gene symbol	Fold change	
	$PS1_{P117L}$ vs Wild type	AD vs non-AD		$PS1_{P117L}$ vs Wild type	AD vs non-AD
ARG2	-6.12	-1.41	*NREP*	-13.78	-1.55
ATP1A3	-12.31	-1.50	*PI4KA*	-9.43	-1.55
ATP2B1	-8.60	-1.49	*R3HDM1*	-6.98	-1.46
BCL11A	-8.55	-1.90	*RAB3A*	-9.35	-1.49
CADPS	-6.20	-1.42	*RUNX1T1*	-9.35	-1.43
CAP2	-9.81	-1.52	*SCN2A*	-7.51	-1.53
CHP1	-6.19	-1.43	*SHC3*	-8.19	-1.45
DCLK1	-11.86	-1.41	*SNAP25*	-7.09	-1.60
ENC1	-6.37	-1.78	*SNAP91*	-7.53	-1.50
GABRA3	-6.86	-1.63	*STXBP1*	-11.79	-1.44
GNG3	-6.88	-1.59	*SYT4*	-6.78	-1.55
GRIN2B	-6.82	-1.87	*TAGLN3*	-6.68	-1.57
INA	-30.91	-1.56	*TMSB10/TMSB4X*	-8.56	-1.49
MAPK9	-6.99	-1.45	*TSPAN7*	-17.48	-1.52
MLLT11	-14.55	-1.50	*USMG5*	-9.98	-1.44
NEDD4L	-15.54	-1.50	*VAMP2*	-11.67	-1.41
NLK	-9.28	-1.57	*WASF1*	-7.31	-1.69
NMNAT2	-10.07	-1.46	*WDR47*	-7.39	-1.50
NNAT	-24.60	-1.54			

Gene symbols in red indicate Alzheimer's disease-related genes.

Supplementary Table S6. List of transcript clusteres which exhibit significantly increased expression in PS1$_{P117L}$+rhTFAM cells.

Transcript cluster ID	Gene symbol	p-value (PS1$_{P117L}$+rhTFAM vs PS1$_{P117L}$)	Fold change** (PS1$_{P117L}$+rhTFAM vs PS1$_{P117L}$)	Raw expression level (log2)*		Standard Deviation (log2)		Gene description
				PS1$_{P117L}$+rhTFAM	PS1$_{P117L}$	PS1$_{P117L}$+rhTFAM	PS1$_{P117L}$	
7981964	SNORD116-8	0.02721	57.28	9.99	4.15	0.98	1.90	small nucleolar RNA, C/D box 116-8; small nucleolar RNA, C/D box 116-3; small nucleolar RNA, C/D box 116-9; small nucleolar RNA, C/D box 116-6
7981996	SNORD116-24	0.02923	46.42	10.20	4.66	1.24	1.61	small nucleolar RNA, C/D box 116-24
7981953	SNORD116-3	0.03553	40.27	10.41	5.08	0.96	2.01	small nucleolar RNA, C/D box 116-3; small nucleolar RNA, C/D box 116-9; small nucleolar RNA, C/D box 116-5; small nucleolar RNA, C/D box 116-7; small nucleolar RNA, C/D box 116-8
7981978	SNORD116-15	0.03876	35.72	11.82	6.66	0.98	1.81	small nucleolar RNA, C/D box 116-15
8154305	SELT	0.02694	31.26	8.32	3.35	0.49	1.96	selenoprotein T
7981958	SNORD116-5	0.02445	26.58	9.25	4.51	1.09	1.62	small nucleolar RNA, C/D box 116-5; small nucleolar RNA, C/D box 116-7; small nucleolar RNA, C/D box 116-3; small nucleolar RNA, C/D box 116-9
8019930	MYL12B	0.02658	26.35	8.70	3.98	0.13	1.87	myosin, light chain 12B, regulatory
8020110	RAB31	0.00691	25.55	9.22	4.55	0.05	1.32	RAB31, member RAS oncogene family
7981982	SNORD116-19	0.04514	25.10	10.88	6.23	0.87	1.70	small nucleolar RNA, C/D box 116-19; small nucleolar RNA, C/D box 116-17; small nucleolar RNA, C/D box 116-20; small nucleolar RNA, C/D box 116-21; small nucleolar RNA, C/D box 116 cluster; small nuclear ribonucleoprotein polypeptide N; small nucleolar RNA, C/D box 116-15; small nucleolar RNA, C/D box 116-16; small nucleolar RNA, C/D box 116-18; small nucleolar RNA, C/D box 116-22; imprinted in Prader-Willi syndrome (non-protein coding); small nucleolar RNA, C/D box 107; Prader Willi/Angelman region RNA, SNRPN neighbor; small nucleolar RNA, C/D box 116-4; small nucleolar RNA, C/D box 115-7; small nucleolar RNA, C/D box 115-13; small nucleolar RNA, C/D box 115-26; small nucleolar RNA, C/D box 116-28; uncharacterized LOC101930404; small nucleolar RNA, C/D box 116-14
8171297	MID1	0.00279	24.84	9.50	4.86	0.23	1.10	midline 1
7981976	SNORD116-14	0.03755	22.72	10.58	6.08	0.94	1.86	small nucleolar RNA, C/D box 116-14; small nuclear ribonucleoprotein polypeptide N
7981988	SNORD116-20	0.01776	21.40	10.42	6.00	0.93	1.38	small nucleolar RNA, C/D box 116-20; small nucleolar RNA, C/D box 116-19; small nucleolar RNA, C/D box 116-17; small nucleolar RNA, C/D box 116-21; small nucleolar RNA, C/D box 116 cluster
8006433	CCL2	0.03491	20.95	10.73	6.34	0.16	1.84	chemokine (C-C motif) ligand 2
7975989	SLIRP	0.02341	20.32	8.79	4.45	0.34	1.63	SRA stem-loop interacting RNA binding protein
8141016	TFPI2	0.00195	20.00	9.83	5.51	0.32	0.94	tissue factor pathway inhibitor 2
8098195	MSMO1	0.00286	19.21	8.34	4.08	0.42	1.05	methylsterol monooxygenase 1
8086752	SNORD13P3	0.02063	17.80	9.69	5.54	0.75	1.57	small nucleolar RNA, C/D box 13 pseudogene 3
8034512	SNORD41	0.00604	17.64	8.27	4.13	1.01	0.71	small nucleolar RNA, C/D box 41
8111941	HMGCS1	0.00019	16.85	8.81	4.74	0.30	0.47	3-hydroxy-3-methylglutaryl-CoA synthase 1 (soluble)
7965467	RPL41	0.03931	16.83	10.22	6.14	0.05	1.85	ribosomal protein L41; ribosomal protein L41 pseudogene 1; ribosomal protein L41 pseudogene 2
8094778	UCHL1	0.01883	16.63	8.87	4.81	0.17	1.45	ubiquitin carboxyl-terminal esterase L1 (ubiquitin thiolesterase)
8155849	ANXA1	0.00126	16.58	8.91	4.86	0.28	0.76	annexin A1
8132070	GARS	0.00053	16.58	8.36	4.31	0.34	0.57	glycyl-tRNA synthetase
7981960	SNORD116-6	0.02380	16.51	7.82	3.77	1.34	0.73	small nucleolar RNA, C/D box 116-6
8110522	CANX	0.01863	16.50	9.84	5.80	0.24	1.45	calnexin
8175531	CDR1	0.04618	16.09	8.90	4.89	0.69	1.86	cerebellar degeneration-related protein 1, 34kDa
8134339	PEG10	0.01358	16.02	10.52	6.52	0.10	1.34	paternally expressed 10
8102800	SLC7A11	0.00034	15.51	8.91	4.96	0.41	0.44	solute carrier family 7 (anionic amino acid transporter light chain, xc- system), member 11
8012110	GABARAP	0.02367	15.27	9.53	5.59	0.19	1.53	GABA(A) receptor-associated protein
8011011	YWHAE	0.01174	14.31	9.90	6.06	0.22	1.23	tyrosine 3-monooxygenase/tryptophan 5-monooxygenase activation protein, epsilon
8047926	MAP2	0.01543	14.30	8.25	4.41	0.08	1.31	microtubule-associated protein 2
7979864	ERH	0.01422	14.09	8.82	5.00	0.38	1.18	enhancer of rudimentary homolog (Drosophila)
8052010	CALM1	0.02110	14.06	7.93	4.11	0.58	1.55	calmodulin 1 (phosphorylase kinase, delta); calmodulin 2 (phosphorylase kinase, delta); calmodulin 3 (phosphorylase kinase, delta)
8002303	NQO1	0.00785	14.06	8.93	5.12	0.26	1.13	NAD(P)H dehydrogenase, quinone 1
8091446	PFN2	0.00343	13.98	8.23	4.43	0.16	0.91	profilin 2
7981949	SNORD116-1	0.04812	13.60	8.29	4.52	1.37	1.22	small nucleolar RNA, C/D box 116-1
7897620	PGD	0.00055	13.58	8.80	5.04	0.56	0.32	phosphogluconate dehydrogenase
8117995	TUBB	0.01560	13.49	9.88	6.13	0.15	1.30	tubulin, beta class I; tubulin, beta pseudogene 1
8114455	HSPA9	0.00130	13.08	8.16	4.45	0.20	0.71	heat shock 70kDa protein 9 (mortalin)
8027002	GDF15	0.00086	12.99	9.13	5.43	0.27	0.59	growth differentiation factor 15
8145793	SNORD13	0.00843	12.76	10.13	6.45	0.75	1.18	small nucleolar RNA, C/D box 13
7948894	RNU2-2P	0.03621	12.75	9.40	5.73	0.86	1.13	RNA, U2 small nuclear 2, pseudogene; RNA, U2 small nuclear 1; WD repeat domain 74; RNA, U2 small nuclear 36, pseudogene
8024436	OAZ1	0.00352	12.46	9.02	5.38	0.15	0.92	ornithine decarboxylase antizyme 1
7981955	SNORD116-4	0.03843	12.30	7.97	4.35	1.03	0.97	small nucleolar RNA, C/D box 116-4; imprinted in Prader-Willi syndrome (non-protein coding); small nucleolar RNA, C/D box 107; Prader Willi/Angelman region RNA, SNRPN neighbor; small nucleolar RNA, C/D box 116-22; small nucleolar RNA, C/D box 115-7; small nucleolar RNA, C/D box 115-13; small nucleolar RNA, C/D box 115-26; small nucleolar RNA, C/D box 116-28; uncharacterized LOC101930404; small nuclear ribonucleoprotein polypeptide N
8116520	GNB2L1	0.01572	12.06	10.53	6.94	0.18	1.23	guanine nucleotide binding protein (G protein), beta polypeptide 2-like 1; small nucleolar RNA, C/D box 95; small nucleolar RNA, C/D box 96A
8115327	SPARC	0.00557	12.03	9.66	6.07	0.35	0.88	secreted protein, acidic, cysteine-rich (osteonectin)
7958174	TXNRD1	0.00413	11.73	8.12	4.56	0.56	0.76	thioredoxin reductase 1
8089062	CLDND1	0.00332	11.69	8.12	4.57	0.33	0.91	claudin domain containing 1
8064375	SRXN1	0.00670	11.64	7.95	4.41	0.72	0.68	sulfiredoxin 1
7963054	TUBA1A	0.02298	11.58	9.23	5.70	0.33	1.30	tubulin, alpha 1a
8124531	HIST1H3I	0.01933	11.57	8.69	5.16	0.03	1.28	histone cluster 1, H3i
7916843	GNG12	0.00323	11.51	8.08	4.55	0.52	0.70	guanine nucleotide binding protein (G protein), gamma 12
7897801	RNU5E-1	0.04806	11.50	9.77	6.24	1.00	1.04	RNA, U5E small nuclear 1
8146216	VDAC3	0.04767	11.46	7.58	4.07	0.89	1.39	voltage-dependent anion channel 3
7982117	GABRG3	0.00032	11.33	8.80	5.30	0.18	0.51	gamma-aminobutyric acid (GABA) A receptor, gamma 3
7981994	SNORD116-23	0.03416	11.24	7.65	4.16	0.91	0.97	small nucleolar RNA, C/D box 116-23
7938291	SNORA45A	0.04881	10.81	9.59	6.16	1.32	0.60	small nucleolar RNA, H/ACA box 45A; ribosomal protein L27a
7926368	VIM	0.01552	10.76	10.53	7.11	0.14	1.20	vimentin
8048847	AGFG1	0.00672	10.69	8.43	5.01	0.32	0.91	ArfGAP with FG repeats 1
8030362	SNORD33	0.01302	10.69	9.62	6.20	1.13	0.51	small nucleolar RNA, C/D box 33; ribosomal protein L13a; small nucleolar RNA, C/D box 35A; small nucleolar RNA, C/D box 34; small nucleolar RNA, C/D box 32A
8004497	EIF4A1	0.01647	10.61	7.78	4.37	0.31	1.33	eukaryotic translation initiation factor 4A1; SENP3-EIF4A1 readthrough (NMD candidate); small nucleolar RNA, H/ACA box 67; small nucleolar RNA, H/ACA box 48; small nucleolar RNA, C/D box 10; uncharacterized LOC101928634
8170009	FAM127A	0.00142	10.55	7.94	4.54	0.15	0.65	family with sequence similarity 127, member A
8094625	KLHL5	0.00997	10.51	7.68	4.29	0.60	0.99	kelch-like family member 5
8101212	CCNI	0.01353	10.42	8.93	5.55	0.10	1.21	cyclin I
7977775	DAD1	0.01282	10.42	9.62	6.24	0.32	1.13	defender against cell death 1
8172022	TMEM47	0.02015	10.35	8.98	5.61	0.18	1.34	transmembrane protein 47
7962537	SLC38A2	0.00677	10.19	7.49	4.14	0.43	0.91	solute carrier family 38, member 2
7945182	APLP2	0.00385	10.07	8.39	5.06	0.35	0.75	amyloid beta (A4) precursor-like protein 2
7911619	GNB1	0.00197	10.06	9.19	5.86	0.19	0.68	guanine nucleotide binding protein (G protein), beta polypeptide 1
8065325	RPS15AP1	0.02527	10.05	7.50	4.17	0.48	1.15	ribosomal protein S15a pseudogene 1
7987225	NOP10	0.00615	10.03	8.08	4.75	0.25	1.01	NOP10 ribonucleoprotein
7949410	MALAT1	0.00860	10.01	7.68	4.36	0.79	0.81	metastasis associated lung adenocarcinoma transcript 1 (non-protein coding)
7942586	RPS3	0.03247	10.01	9.45	6.13	0.37	1.33	ribosomal protein S3
7966135	CORO1C	0.00143	9.95	8.02	4.71	0.60	0.33	coronin, actin binding protein, 1C
8135774	PTPRZ1	0.00501	9.89	8.07	4.77	0.39	0.92	protein tyrosine phosphatase, receptor-type, Z polypeptide 1
8152096	YWHAZ	0.00578	9.88	8.88	5.57	0.24	0.91	tyrosine 3-monooxygenase/tryptophan 5-monooxygenase activation protein, zeta; tyrosine 3-monooxygenase/tryptophan 5-monooxygenase activation protein, zeta pseudogene 3
7969003	ITM2B	0.00562	9.87	7.85	4.55	0.22	0.87	integral membrane protein 2B
8117054	CAP2	0.00217	9.86	7.92	4.62	0.17	0.67	CAP, adenylate cyclase-associated protein, 2 (yeast)
8074925	GUSBP2	0.01387	9.81	8.12	4.83	0.55	0.95	glucuronidase, beta pseudogene 2; glucuronidase, beta pseudogene; glucuronidase, beta pseudogene 11; glucuronidase, beta pseudogene 1; glucuronidase, beta pseudogene 4; beta-glucuronidase-like protein SMA4-like; glucuronidase, beta pseudogene 9; glucuronidase, beta; uncharacterized LOC102725134
8052204	RTN4	0.01269	9.79	7.57	4.28	0.27	1.29	reticulon 4

Supplementary Table S6. (continued)

Transcript cluster ID	Gene symbol	p-value ($PS1_{P117L}$+rhTFAM vs $PS1_{P117L}$)	Fold change** ($PS1_{P117L}$+rhTFAM vs $PS1_{P117L}$)	Raw expression level (log2)*		Standard Deviation (log2)		Gene description
				$PS1_{P117L}$+rhTFAM	$PS1_{P117L}$	$PS1_{P117L}$+rhTFAM	$PS1_{P117L}$	
7944656	SC5D	0.00030	9.79	7.43	4.13	0.45	0.22	sterol-C5-desaturase
8000217	SMG1	0.00603	9.75	8.43	5.14	0.32	0.91	SMG1 phosphatidylinositol 3-kinase-related kinase; SMG1 pseudogene 5; SMG1 pseudogene 2; SMG1 pseudogene 1; SMG1 pseudogene 3; serine/threonine-protein kinase SMG1-like; bolA family member 2; solute carrier family 7 (amino acid transporter light chain, L system), member 5 pseudogene 1; putative L-type amino acid transporter 1-like protein IMAA-like; SMG1 pseudogene 7
8168416	USMG5	0.00158	9.69	8.59	5.32	0.14	0.66	up-regulated during skeletal muscle growth 5 homolog (mouse); USMG5 pseudogene 1
8151935	RPL30	0.01914	9.66	9.31	6.04	0.02	1.46	ribosomal protein L30
7976598	PAPOLA	0.01952	9.61	7.47	4.21	0.22	1.21	poly(A) polymerase alpha
8166469	SAT1	0.00676	9.57	8.65	5.39	0.58	0.86	spermidine/spermine N1-acetyltransferase 1
8096301	SPP1	0.00909	9.52	9.25	6.00	0.38	0.94	secreted phosphoprotein 1
8106098	MAP1B	0.00288	9.51	10.38	7.13	0.14	0.77	microtubule-associated protein 1B
8119993	HSP90AB1	0.00477	9.40	8.58	5.35	0.18	0.85	heat shock protein 90kDa alpha (cytosolic), class B member 1
8017210	AP1S2	0.00780	9.30	7.61	4.39	0.32	1.17	adaptor-related protein complex 1, sigma 2 subunit; adaptor-related protein complex 1, sigma 2 subunit pseudogene
7912198	ENO1	0.00517	9.30	9.67	6.45	0.19	0.99	enolase 1, (alpha)
8058765	FN1	0.01374	9.28	8.21	5.00	0.20	1.04	fibronectin 1
7946610	EIF4G2	0.00003	9.24	8.57	5.36	0.19	0.19	eukaryotic translation initiation factor 4 gamma, 2
8082431	RAB7A	0.00349	9.24	9.34	6.13	0.31	0.84	RAB7A, member RAS oncogene family
7961693	LDHB	0.00021	9.23	8.87	5.67	0.16	0.44	lactate dehydrogenase B
7933084	NAMPT	0.00490	9.20	7.78	4.58	0.60	0.85	nicotinamide phosphoribosyltransferase; nicotinamide phosphoribosyltransferase-like
7898875	RPL11	0.01990	9.17	8.29	5.09	0.35	1.20	ribosomal protein L11
7929816	SCD	0.00088	9.17	9.09	5.90	0.15	0.58	stearoyl-CoA desaturase (delta-9-desaturase)
7964234	ATP5B	0.00640	9.13	9.27	6.08	0.17	1.04	ATP synthase, H+ transporting, mitochondrial F1 complex, beta polypeptide
8162047	HNRNPK	0.00160	9.11	7.79	4.60	0.07	0.72	heterogeneous nuclear ribonucleoprotein K
7981335	HSP90AA1	0.00046	9.10	8.93	5.75	0.48	0.29	heat shock protein 90kDa alpha (cytosolic), class A member 1
7996947	CYB5B	0.00672	9.07	7.64	4.46	0.38	0.89	cytochrome b5 type B (outer mitochondrial membrane)
8109750	RPLP0	0.00840	9.07	10.01	6.83	0.07	1.12	ribosomal protein, large, P0; ribosomal protein, large, P0 pseudogene 6
8111677	LIFR	0.00703	9.00	7.71	4.54	0.20	1.08	leukemia inhibitory factor receptor alpha
7990916	RPS17	0.01120	9.00	9.24	6.07	0.21	0.96	ribosomal protein S17
7920123	S100A10	0.00438	8.95	8.76	5.59	0.27	0.79	S100 calcium binding protein A10
8144669	FDFT1	0.00035	8.94	8.74	5.58	0.32	0.38	farnesyl-diphosphate farnesyltransferase 1
8131666	ITGB8	0.00136	8.93	8.58	5.43	0.37	0.51	integrin, beta 8
8146967	CRISPLD1	0.04611	8.92	7.10	3.94	0.80	1.26	cysteine-rich secretory protein LCCL domain containing 1
8060503	SNORD57	0.00309	8.90	8.55	5.39	0.54	0.66	small nucleolar RNA, C/D box 57; NOP56 ribonucleoprotein; small nucleolar RNA, C/D box 86; small nucleolar RNA, C/D box 110; microRNA 1292
8163185	TXN	0.00843	8.82	8.19	5.04	0.68	1.07	thioredoxin
7940565	FADS2	0.00251	8.79	8.73	5.59	0.18	0.80	fatty acid desaturase 2
7959023	MAP1LC3B2	0.00003	8.79	8.55	5.42	0.19	0.21	microtubule-associated protein 1 light chain 3 beta 2; microtubule-associated protein 1 light chain 3 beta
8076511	RPL5	0.01071	8.72	10.23	7.11	0.08	0.98	ribosomal protein L5; small nucleolar RNA, C/D box 21; ribosomal protein L5 pseudogene 34
7905329	MLLT11	0.01270	8.67	10.16	7.04	0.18	1.01	myeloid/lymphoid or mixed-lineage leukemia (trithorax homolog, Drosophila); translocated to, 11
8018966	TIMP2	0.01109	8.63	8.24	5.13	0.23	1.01	TIMP metallopeptidase inhibitor 2; differential display clone 8
8061772	MAPRE1	0.00671	8.62	7.19	4.08	0.40	0.76	microtubule-associated protein, RP/EB family, member 1
8007228	ATP6V0A1	0.00598	8.60	7.24	4.14	0.47	0.79	ATPase, H+ transporting, lysosomal V0 subunit a1
7918825	CSDE1	0.02334	8.58	8.28	5.18	0.32	1.54	cold shock domain containing E1, RNA-binding
7999468	LITAF	0.00005	8.58	8.35	5.25	0.27	0.12	lipopolysaccharide-induced TNF factor
8167069	UBA1	0.00088	8.56	8.47	5.37	0.21	0.51	ubiquitin-like modifier activating enzyme 1
8062880	YWHAB	0.00071	8.49	7.84	4.76	0.49	0.32	tyrosine 3-monooxygenase/tryptophan 5-monooxygenase activation protein, beta
7958130	HSP90B1	0.00061	8.46	8.27	5.19	0.47	0.24	heat shock protein 90kDa beta (Grp94), member 1; microRNA 3652
8178050	CSNK2B	0.00095	8.42	8.80	5.72	0.09	0.60	casein kinase 2, beta polypeptide
8158372	SET	0.00102	8.41	8.10	5.03	0.14	0.60	SET nuclear proto-oncogene; SET-like protein
8088339	ARF4	0.00030	8.40	7.88	4.80	0.01	0.45	ADP-ribosylation factor 4
8113469	PJA2	0.00062	8.36	7.62	4.55	0.30	0.45	praja ring finger 2, E3 ubiquitin protein ligase
7911339	MT-TN	0.00768	8.33	10.37	7.32	0.20	0.87	mitochondrially encoded tRNA asparagine
8007141	EIF1	0.00529	8.27	10.16	7.11	0.29	0.82	eukaryotic translation initiation factor 1
8146550	SDCBP	0.00214	8.27	8.49	5.44	0.15	0.74	syndecan binding protein (syntenin)
7968297	POMP	0.01679	8.25	7.79	4.74	0.21	1.10	proteasome maturation protein
7967563	UBC	0.01701	8.24	11.19	8.14	0.14	1.09	ubiquitin C
7910014	CNIH4	0.00036	8.21	6.98	3.95	0.30	0.31	cornichon family AMPA receptor auxiliary protein 4
8061114	DSTN	0.00184	8.10	6.87	3.85	0.41	0.67	destrin (actin depolymerizing factor)
7906061	SYT11	0.00837	8.04	7.42	4.41	0.61	0.68	synaptotagmin XI
8056734	TLK1	0.00514	8.04	7.34	4.33	0.26	0.80	tousled-like kinase 1
8102415	CAMK2D	0.00016	8.03	7.20	4.19	0.36	0.08	calcium/calmodulin-dependent protein kinase II delta
8008834	CLTC	0.00075	8.01	8.09	5.09	0.35	0.46	clathrin, heavy chain (Hc)

*Average raw expression level (log2) in $PS1_{P117L}$+rhTFAM cells >6.6. ** Fold change >8 with p <0.05 (unpaired t-test).
Gene symbols in red indicate Alzheimer's disease-related genes.

Supplementary Table S7. List of transcript clusteres which exhibit significantly increased expression in AD hippocampus.

Transcript cluster ID	Gene symbol	p-value (AD vs non-AD)	Fold change** (AD vs non-AD)	Raw expression level (log2)*		Standard Deviation (log2)		Gene description
				AD	non-AD	AD	non-AD	
8155849	ANXA1	0.013106	2.91	10.77	9.23	0.87	0.73	annexin A1
7939341	CD44	0.016508	2.23	10.42	9.26	0.94	0.89	CD44 molecule (Indian blood group)
8013364	SLC47A2	0.009655	2.19	7.42	6.29	0.75	0.59	solute carrier family 47 (multidrug and toxin extrusion), member 2
7986350	ARRDC4	0.001075	2.13	10.05	8.96	0.73	0.29	arrestin domain containing 4
8166690	CHDC2	0.010639	2.10	7.45	6.38	0.86	0.57	calponin homology domain containing 2
7983523	RNU6-1014P	0.007630	2.06	7.57	6.53	0.84	0.56	RNA, U6 small nuclear 1014, pseudogene
8175666	GABRE	0.019813	2.02	8.18	7.16	0.74	0.52	gamma-aminobutyric acid (GABA) A receptor, epsilon; microRNA 224; microRNA 452
8132557	AEBP1	0.003424	1.99	9.78	8.79	0.72	0.32	AE binding protein 1
7907222	PRRX1	0.005844	1.88	8.83	7.93	0.68	0.38	paired related homeobox 1
7948167	APLNR	0.023326	1.87	10.43	9.53	0.89	0.62	apelin receptor
8162404	ECM2	0.010828	1.87	7.55	6.65	0.87	0.29	extracellular matrix protein 2, female organ and adipocyte specific
8135990	FLNC	0.031860	1.84	8.75	7.87	0.76	0.40	filamin C, gamma
8155707	TJP2	0.013743	1.81	10.24	9.39	0.74	0.41	tight junction protein 2
8169931	ARHGAP36	0.041393	1.80	7.99	7.14	1.16	0.32	Rho GTPase activating protein 36
8147516	MATN2	0.020150	1.80	8.86	8.02	0.53	0.34	matrilin 2; uncharacterized LOC100506558
7904726	TXNIP	0.011178	1.80	12.06	11.21	0.38	0.76	thioredoxin interacting protein
8089145	ABI3BP	0.026714	1.74	9.08	8.28	0.71	0.52	ABI family, member 3 (NESH) binding protein
8158431	PHYHD1	0.011705	1.74	10.48	9.67	0.63	0.34	phytanoyl-CoA dioxygenase domain containing 1
8172043	SRPX	0.025506	1.71	8.35	7.57	0.76	0.37	sushi-repeat containing protein, X-linked
8078155	GALNT15	0.001312	1.69	9.55	8.80	0.71	0.44	polypeptide N-acetylgalactosaminyltransferase 15
7954398	SPX	0.006870	1.68	8.94	8.19	0.60	0.29	spexin hormone
8091385	CP	0.045595	1.65	10.00	9.28	0.86	1.40	ceruloplasmin (ferroxidase)
7954090	EMP1	0.043753	1.65	9.31	8.59	0.74	0.76	epithelial membrane protein 1
8116921	EDN1	0.020688	1.64	8.23	7.51	0.54	0.37	endothelin 1
8121749	GJA1	0.008571	1.64	11.16	10.44	0.50	0.41	gap junction protein, alpha 1, 43kDa
8007454	RND2	0.008222	1.64	10.05	9.33	0.58	0.26	Rho family GTPase 2
8012837	PIRT	0.009119	1.63	8.65	7.94	0.78	0.32	phosphoinositide-interacting regulator of transient receptor potential channels
8179322	HSPA1A	0.039579	1.62	12.18	11.49	0.57	0.43	heat shock 70kDa protein 1A; heat shock 70kDa protein 1B; heat shock 70kDa protein 1-like

*Average raw expression level (log2) in non-AD >6.6. **Fold change >1.6 with p <0.05 (unpaired t-test).
Expression data from 7 AD hiipocampus (NCBI GEO DATA sets: GSM907854-9807834) and 10 non-AD hippocumpus (NCBI GEO DATA sets: GSM907861-907870) were reanalyzed using the Transcriptome Analysis Console 3.0 software (Affimetrix).

Supplementary Table S8. List of transcript clusteres which exhibit significantly decreased expression in AD hippocampus.

Transcript cluster ID	Gene symbol	p-value (AD vs non-AD)	Fold change** (AD vs non-AD)	Raw expression level (log2)*		Standard Deviation (log2)		Gene description
				AD	non-AD	AD	non-AD	
7964722	WIF1	0.003979	-2.63	8.17	9.57	1.02	0.49	WNT inhibitory factor 1
8113234	PCSK1	0.002595	-2.51	7.94	9.27	1.05	0.52	proprotein convertase subtilisin/kexin type 1
8075838	PVALB	0.002622	-2.47	6.97	8.27	0.49	0.72	parvalbumin
7906919	RGS4	0.005034	-2.44	10.44	11.72	1.20	0.32	regulator of G-protein signaling 4
7925761	OR14I1	0.020368	-2.33	7.50	8.72	0.89	0.59	olfactory receptor, family 14, subfamily I, member 1
8138882	NEUROD6	0.000740	-2.30	7.79	9.00	0.64	0.54	neuronal differentiation 6
8135601	MET	0.000762	-2.27	7.50	8.68	0.76	0.72	MET proto-oncogene, receptor tyrosine kinase
8109663	GABRA1	0.000465	-2.25	10.33	11.50	0.79	0.25	gamma-aminobutyric acid (GABA) A receptor, alpha 1
7994131	PRKCB	0.004647	-2.22	10.00	11.15	0.80	0.43	protein kinase C, beta
7943319	CNTN5	0.019808	-2.21	7.80	8.94	0.46	0.75	contactin 5
8152119	NCALD	0.001142	-2.21	9.32	10.47	0.70	0.41	neurocalcin delta
7899841	HPCA	0.000236	-2.19	10.36	11.49	0.42	0.42	hippocalcin
8107764	CTXN3	0.016117	-2.16	6.61	7.73	0.64	0.69	cortexin 3
7971526	HTR2A	0.033828	-2.16	8.70	9.81	1.05	0.85	5-hydroxytryptamine (serotonin) receptor 2A, G protein-coupled
7994152	CACNG3	0.001330	-2.15	9.54	10.64	0.63	0.43	calcium channel, voltage-dependent, gamma subunit 3
7901342	ELAVL4	0.001348	-2.07	8.52	9.57	0.69	0.27	ELAV like neuron-specific RNA binding protein 4
8134463	NPTX2	0.001502	-2.06	8.78	9.82	0.32	0.65	neuronal pentraxin II
7958860	RPH3A	0.004472	-2.04	9.38	10.40	0.83	0.50	rabphilin 3A homolog (mouse)
8067157	CBLN4	0.029786	-2.03	7.84	8.86	0.79	0.65	cerebellin 4 precursor
7999364	GRIN2A	0.002357	-2.02	9.54	10.55	0.41	0.45	glutamate receptor, ionotropic, N-methyl D-aspartate 2A
7986195	SV2B	0.003140	-1.99	11.21	12.20	0.79	0.27	synaptic vesicle glycoprotein 2B
7969665	HS6ST3	0.000473	-1.97	8.89	9.87	0.45	0.33	heparan sulfate 6-O-sulfotransferase 3
8066347	PTPRT	0.008047	-1.97	9.02	9.99	0.70	0.38	protein tyrosine phosphatase, receptor type, T
7979721	TMEM229B	0.000636	-1.97	6.79	7.77	0.40	0.42	transmembrane protein 229B
7979529	KCNH5	0.028327	-1.94	7.13	8.09	0.80	0.54	potassium voltage-gated channel, subfamily H (eag-related), member 5
8099524	LDB2	0.006118	-1.93	9.14	10.09	0.58	0.42	LIM domain binding 2
8059279	EPHA4	0.001835	-1.92	10.03	10.97	0.60	0.38	EPH receptor A4
8003667	SERPINF1	0.000003	-1.92	7.90	8.84	0.36	0.17	serpin peptidase inhibitor, clade F (alpha-2 antiplasmin, pigment epithelium derived factor), member 1
8154953	KIAA1045	0.000379	-1.91	8.99	9.93	0.51	0.26	KIAA1045
8052399	BCL11A	0.000731	-1.90	8.66	9.59	0.48	0.32	B-cell CLL/lymphoma 11A (zinc finger protein)
8080918	FAM19A1	0.005712	-1.90	7.85	8.77	0.65	0.61	family with sequence similarity 19 (chemokine (C-C motif)-like), member A1
7939215	KIAA1549L	0.004584	-1.90	9.44	10.36	0.61	0.40	KIAA1549-like
8085716	SATB1	0.000105	-1.90	9.58	10.51	0.44	0.20	SATB homeobox 1
7936249	SORCS1	0.040611	-1.90	7.75	8.68	0.71	0.53	sortilin-related VPS10 domain containing receptor 1
8149720	EGR3	0.008593	-1.89	7.52	8.44	0.39	0.57	early growth response 3
7966172	SVOP	0.003861	-1.89	9.30	10.21	0.66	0.42	SV2 related protein homolog (rat)
8150419	ZMAT4	0.001638	-1.89	7.99	8.91	0.39	0.38	zinc finger, matrin-type 4
8164937	FAM163B	0.010025	-1.87	9.72	10.62	0.52	0.49	family with sequence similarity 163, member B
7961422	GRIN2B	0.005338	-1.87	10.09	10.99	0.53	0.45	glutamate receptor, ionotropic, N-methyl D-aspartate 2B
8031057	PRKCG	0.036215	-1.87	9.98	10.88	0.35	0.75	protein kinase C, gamma
8120300	MLIP	0.020879	-1.86	8.87	9.76	1.07	0.37	muscular LMNA-interacting protein
7948588	SYT7	0.001031	-1.86	9.26	10.16	0.45	0.25	synaptotagmin VII
7975066	AKAP5	0.012745	-1.85	8.17	9.06	0.49	0.65	A kinase (PRKA) anchor protein 5
8111998	HCN1	0.000739	-1.85	10.44	11.33	0.59	0.32	hyperpolarization activated cyclic nucleotide-gated potassium channel 1
8146908	KCNB2	0.002459	-1.85	7.62	8.51	0.36	0.37	potassium voltage-gated channel, Shab-related subfamily, member 2
8163149	PTPN3	0.001715	-1.85	7.12	8.01	0.42	0.49	protein tyrosine phosphatase, non-receptor type 3
8112333	HTR1A	0.047208	-1.84	8.29	9.17	0.63	0.85	5-hydroxytryptamine (serotonin) receptor 1A, G protein-coupled
8152314	RSPO2	0.029654	-1.84	7.67	8.55	0.62	0.51	R-spondin 2
8022976	RIT2	0.000901	-1.83	7.13	8.01	0.55	0.28	Ras-like without CAAX 2
7909689	SMYD2	0.000255	-1.83	7.96	8.84	0.35	0.26	SET and MYND domain containing 2
8048112	VWC2L	0.009796	-1.83	7.16	8.03	0.75	0.54	von Willebrand factor C domain containing protein 2-like
8149629	GFRA2	0.000179	-1.82	8.83	9.69	0.46	0.29	GDNF family receptor alpha 2
8035318	UNC13A	0.013400	-1.82	9.22	10.08	0.50	0.39	unc-13 homolog A (C. elegans)
8044976	CNTNAP5	0.005350	-1.80	9.15	10.00	0.61	0.44	contactin associated protein-like 5
7952350	SCN3B	0.006170	-1.80	10.81	11.66	0.59	0.40	sodium channel, voltage-gated, type III, beta subunit
7979663	RAB15	0.000055	-1.79	9.34	10.18	0.45	0.21	RAB15, member RAS oncogene family
8155864	RORB	0.039148	-1.79	8.55	9.39	0.46	0.67	RAR-related orphan receptor B
8112615	ENC1	0.000343	-1.78	10.86	11.70	0.50	0.25	ectodermal-neural cortex 1 (with BTB domain)
8105596	RGS7BP	0.001764	-1.78	10.03	10.86	0.50	0.34	regulator of G-protein signaling 7 binding protein
8085311	ATP2B2	0.002424	-1.77	10.12	10.94	0.41	0.41	ATPase, Ca++ transporting, plasma membrane 2
8129254	MAN1A1	0.000281	-1.77	8.57	9.39	0.30	0.28	mannosidase, alpha, class 1A, member 1
7966026	NUAK1	0.002568	-1.77	8.94	9.76	0.60	0.33	NUAK family, SNF1-like kinase, 1
7950162	PDE2A	0.000459	-1.77	9.84	10.66	0.46	0.26	phosphodiesterase 2A, cGMP-stimulated
8000757	DOC2A	0.004144	-1.76	9.13	9.95	0.41	0.41	double C2-like domains, alpha
7897236	KCNAB2	0.002293	-1.76	10.40	11.21	0.38	0.34	potassium voltage-gated channel, shaker-related subfamily, beta member 2
8015049	KRT222	0.001420	-1.76	7.08	7.89	0.48	0.32	keratin 222
7974895	LINC00643	0.004401	-1.76	7.93	8.75	0.43	0.47	long intergenic non-protein coding RNA 643
8095751	PARM1	0.003425	-1.76	10.00	10.81	0.71	0.27	prostate androgen-regulated mucin-like protein 1
8077376	ITPR1	0.044290	-1.75	8.81	9.62	0.70	0.44	inositol 1,4,5-trisphosphate receptor, type 1
8160546	LINGO2	0.049546	-1.75	8.35	9.16	0.52	0.66	leucine rich repeat and Ig domain containing 2
8086615	LRRC2	0.002718	-1.75	6.45	7.26	0.69	0.32	leucine rich repeat containing 2
8076169	NPTXR	0.000031	-1.75	9.91	10.72	0.25	0.26	neuronal pentraxin receptor
8041225	EHD3	0.000040	-1.74	9.38	10.18	0.27	0.24	EH-domain containing 3
8161526	LOC440896	0.014682	-1.74	7.18	7.99	0.51	0.72	uncharacterized LOC440896; uncharacterized LOC101927424; uncharacterized LOC101929800; uncharacterized LOC101929179
7950578	PAK1	0.006495	-1.74	10.56	11.36	0.68	0.44	p21 protein (Cdc42/Rac)-activated kinase 1
8100507	HOPX	0.000048	-1.73	7.81	8.60	0.39	0.19	HOP homeobox
7918052	OLFM3	0.014245	-1.73	7.49	8.28	0.62	0.48	olfactomedin 3
8076644	SULT4A1	0.003988	-1.73	9.42	10.22	0.61	0.24	sulfotransferase family 4A, member 1
7939024	ANO3	0.004631	-1.72	8.34	9.12	0.63	0.43	anoctamin 3
8027770	FXYD7	0.001571	-1.72	9.98	10.76	0.48	0.32	FXYD domain containing ion transport regulator 7
7982102	GABRA5	0.000444	-1.72	10.12	10.90	0.41	0.26	gamma-aminobutyric acid (GABA) A receptor, alpha 5
8070279	KCNJ6	0.000365	-1.72	8.09	8.87	0.34	0.25	potassium inwardly-rectifying channel, subfamily J, member 6
8049532	LRRFIP1	0.002474	-1.72	7.78	8.56	0.53	0.23	leucine rich repeat (in FLII) interacting protein 1
8147848	OXR1	0.000977	-1.72	9.42	10.21	0.61	0.14	oxidation resistance 1
7948912	CHRM1	0.000582	-1.71	9.03	9.81	0.33	0.29	cholinergic receptor, muscarinic 1
8077572	CPNE9	0.027957	-1.71	6.83	7.60	0.40	0.66	copine family member IX
7914342	FABP3	0.000322	-1.71	10.08	10.86	0.31	0.32	fatty acid binding protein 3, muscle and heart (mammary-derived growth inhibitor)
8148040	MAL2	0.008553	-1.71	11.44	12.21	0.71	0.32	mal, T-cell differentiation protein 2 (gene/pseudogene)
7965918	NT5DC3	0.003147	-1.71	9.17	9.95	0.54	0.21	5-nucleotidase domain containing 3
8116932	PHACTR1	0.000594	-1.71	9.45	10.23	0.42	0.29	phosphatase and actin regulator 1
8056327	GRB14	0.017449	-1.70	6.89	7.65	0.43	0.63	growth factor receptor-bound protein 14
8130408	IPCEF1	0.009008	-1.70	9.53	10.29	0.67	0.34	interaction protein for cytohesin exchange factors 1; CNKSR family member 3
7918449	KCNA2	0.002838	-1.70	8.85	9.62	0.38	0.34	potassium voltage-gated channel, shaker-related subfamily, member 2
8133258	WBSCR17	0.000283	-1.70	8.90	9.66	0.48	0.21	Williams-Beuren syndrome chromosome region 17
8120654	KCNQ5	0.019802	-1.69	9.11	9.87	0.78	0.45	potassium voltage-gated channel, KQT-like subfamily, member 5
7990643	LINGO1	0.000397	-1.69	8.12	8.88	0.34	0.37	leucine rich repeat and Ig domain containing 1
8007607	RUNDC3A	0.002381	-1.69	11.24	12.00	0.35	0.36	RUN domain containing 3A
8136115	STRIP2	0.020054	-1.69	7.38	8.14	0.23	0.63	striatin interacting protein 2
8128818	WASF1	0.005046	-1.69	10.29	11.05	0.41	0.29	WAS protein family, member 1
7976073	FLRT2	0.017541	-1.68	8.83	9.58	0.43	0.40	fibronectin leucine rich transmembrane protein 2; uncharacterized LOC100506718
8145361	NEFM	0.017271	-1.68	10.31	11.05	0.56	0.42	neurofilament, medium polypeptide
8019074	NPTX1	0.020210	-1.68	10.30	11.05	0.35	0.51	neuronal pentraxin I

Supplementary Table S8. (continued)

Transcript cluster ID	Gene symbol	p-value (AD vs non-AD)	Fold change** (AD vs non-AD)	Raw expression level (log2)*		Standard Deviation (log2)		Gene description
				AD	non-AD	AD	non-AD	
8098782	CPLX1	0.000103	-1.67	9.46	10.20	0.34	0.24	complexin 1
8026926	MAST3	0.003840	-1.67	10.67	11.41	0.28	0.36	microtubule associated serine/threonine kinase 3
7967660	RIMBP2	0.000069	-1.67	9.48	10.22	0.27	0.22	RIMS binding protein 2
8178508	ATP6V1G2	0.000175	-1.66	11.02	11.76	0.41	0.16	ATPase, H+ transporting, lysosomal 13kDa, V1 subunit G2; ATP6V1G2-DDX39B readthrough (NMD candidate); DEAD (Asp-Glu-Ala-Asp) box polypeptide 39B
8162254	DIRAS2	0.016401	-1.66	11.06	11.79	0.75	0.33	DIRAS family, GTP-binding RAS-like 2
7955502	SCN8A	0.010902	-1.66	9.69	10.42	0.49	0.40	sodium channel, voltage gated, type VIII, alpha subunit
8170671	ATP2B3	0.001438	-1.65	8.92	9.64	0.37	0.33	ATPase, Ca++ transporting, plasma membrane 3
8053349	LRRTM1	0.012826	-1.65	8.90	9.63	0.38	0.56	leucine rich repeat transmembrane neuronal 1
8123739	NRN1	0.000201	-1.65	11.34	12.06	0.41	0.23	neuritin 1
8149699	PHYHIP	0.030330	-1.65	10.36	11.08	0.39	0.49	phytanoyl-CoA 2-hydroxylase interacting protein
7993083	RBFOX1	0.007251	-1.65	9.75	10.47	0.51	0.35	RNA binding protein, fox-1 homolog (C. elegans) 1
8140113	STX1A	0.006379	-1.65	9.31	10.03	0.57	0.29	syntaxin 1A (brain)
8077879	SYN2	0.005129	-1.65	11.34	12.07	0.54	0.33	synapsin II
8107307	CAMK4	0.034229	-1.64	9.42	10.13	0.57	0.44	calcium/calmodulin-dependent protein kinase IV
8054338	CREG2	0.009623	-1.64	9.65	10.37	0.41	0.43	cellular repressor of E1A-stimulated genes 2
8046792	DUSP19	0.046424	-1.64	6.41	7.13	0.35	0.42	dual specificity phosphatase 19
7994052	HS3ST2	0.008928	-1.64	8.21	8.93	0.42	0.57	heparan sulfate (glucosamine) 3-O-sulfotransferase 2
7935361	SLIT1	0.006159	-1.64	9.21	9.92	0.32	0.35	slit homolog 1 (Drosophila)
8157463	C9orf91	0.001493	-1.63	8.91	9.61	0.46	0.25	chromosome 9 open reading frame 91
8175696	GABRA3	0.006911	-1.63	9.73	10.44	0.78	0.44	gamma-aminobutyric acid (GABA) A receptor, alpha 3
8135405	GPR22	0.030835	-1.63	8.10	8.81	0.70	0.36	G protein-coupled receptor 22
7962375	PRICKLE1	0.009569	-1.63	9.26	9.96	0.42	0.35	prickle homolog 1 (Drosophila)
8021301	RAB27B	0.004728	-1.63	9.91	10.62	0.95	0.26	RAB27B, member RAS oncogene family
7925457	RGS7	0.000678	-1.63	9.31	10.01	0.56	0.15	regulator of G-protein signaling 7
7952268	THY1	0.000757	-1.63	10.90	11.60	0.29	0.25	Thy-1 cell surface antigen
8113577	TRIM36	0.003203	-1.63	7.70	8.40	0.47	0.35	tripartite motif containing 36
8132036	WIPF3	0.000434	-1.63	8.34	9.05	0.26	0.34	WAS/WASL interacting protein family, member 3
8111533	LMBRD2	0.003320	-1.62	9.49	10.19	0.42	0.20	LMBR1 domain containing 2
7938925	NELL1	0.005644	-1.62	8.58	9.28	0.54	0.48	NEL-like 1 (chicken)
7902435	TPI1P1	0.000077	-1.62	9.68	10.37	0.36	0.17	triosephosphate isomerase 1 pseudogene 1
7905918	EFNA3	0.000223	-1.61	8.63	9.31	0.31	0.27	ephrin-A3
7962455	NELL2	0.005204	-1.61	10.60	11.29	0.50	0.32	NEL-like 2 (chicken)
7956524	PIP4K2C	0.002968	-1.61	8.91	9.60	0.25	0.28	phosphatidylinositol-5-phosphate 4-kinase, type II, gamma
7990774	RASGRF1	0.001767	-1.61	9.95	10.64	0.48	0.34	Ras protein-specific guanine nucleotide-releasing factor 1
8140398	YWHAG	0.000287	-1.61	10.97	11.66	0.34	0.19	tyrosine 3-monooxygenase/tryptophan 5-monooxygenase activation protein, gamma

*Average raw expression level (log2) in non-AD >6.6. **Fold change <−1.6 with p <0.05 (unpaired t-test).

Expression data from 7 AD hiipocampus (NCBI GEO DATA sets: GSM907854-9807834) and 10 non-AD hippocumpus (NCBI GEO DATA sets: GSM907861-907870) were reanalyzed using the Transcriptome Analysis Console 3.0 software (Affimetrix).

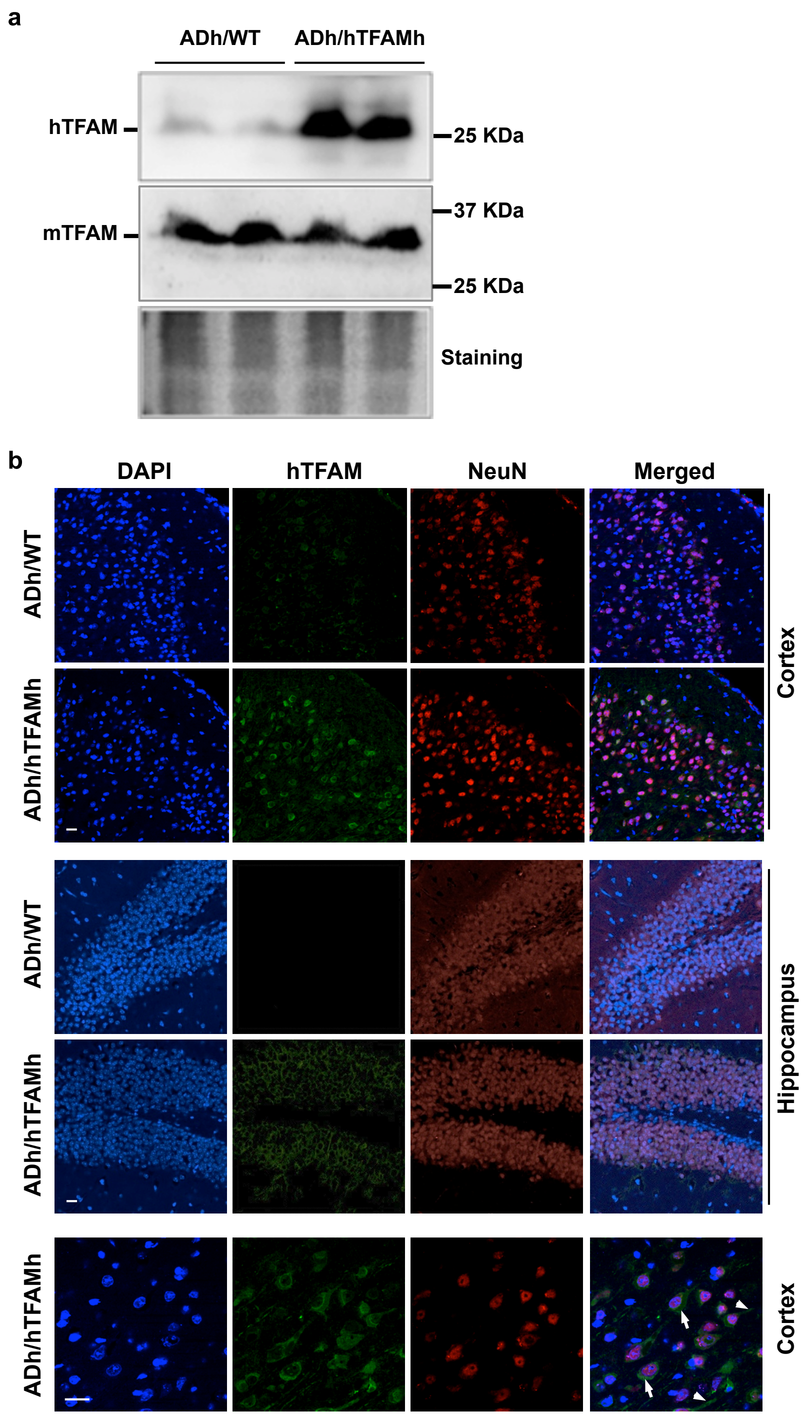

Supplementary Figure S1. Characterization of 13-month-old ADh/hTFAMh mice. (a) Western blot analysis of human and mouse TFAM proteins in the cerebral cortex from 13-month-old ADh/WT or ADh/hTFAMh mice. Total protein extracts (20 µg) were probed with anti-hTFAM (hTFAM, top panel). The blot was reprobed with anti-mTFAM antibody (mTFAM, middle panel). Ponceau S staining was used as the loading control (lower panel). (b) Double-immunofluorescence microscopy with anti-hTFAM and anti-NeuN, a marker of neuronal nuclei. Nuclei were stained with DAPI. Signals for hTFAM and NeuN co-localize in the cerebral cortex and hippocampus of 13-month-old ADh/hTFAMh mice. In the bottom right panel, the expression of hTFAM (green) in cell bodies and neurites is indicated with arrowheads and arrows, respectively. Scale bar, 20 µm.

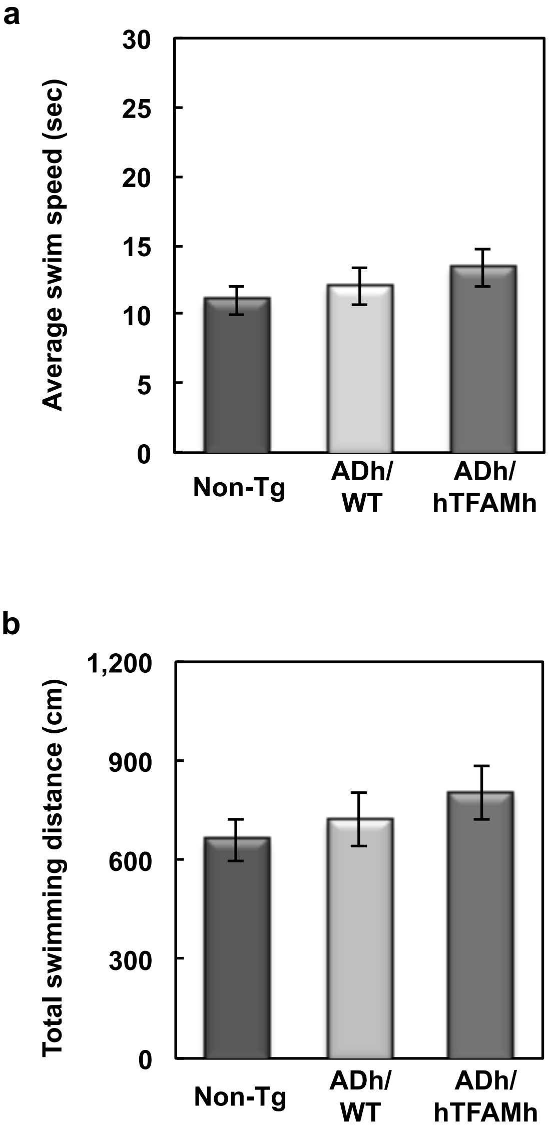

Supplementary Figure S2. ADh/WT and ADh/hTFAMh mice exhibited equal swimming ability in Morris water maze. (a) Average swim speed, and (b) total swimming distance in d2 protocol of Morris water maze. One-way ANOVA, $p = 0.2786$, $p = 0.2811$, respectively.

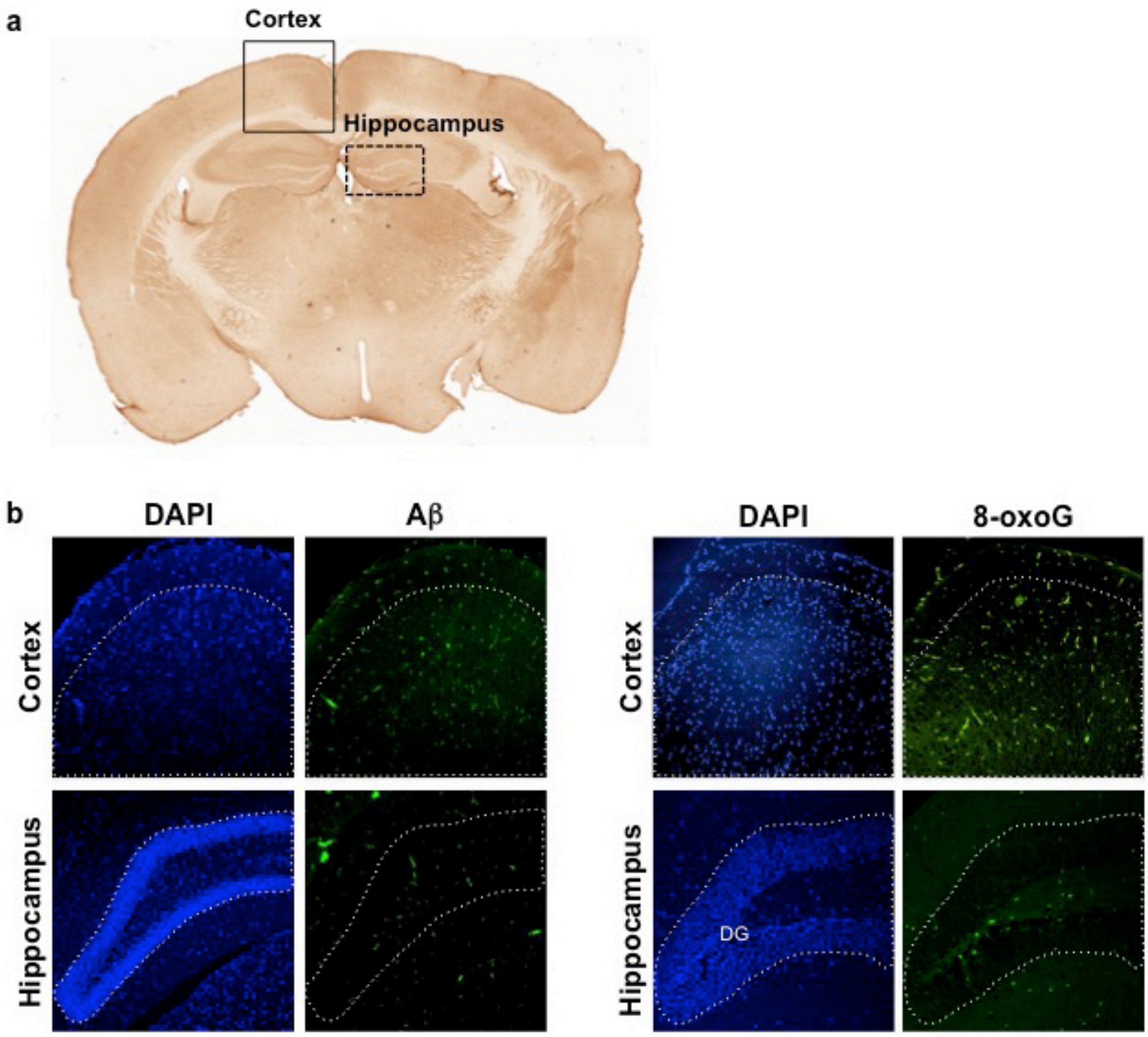

Supplementary Figure S3. **Quantitative measurement of immunofluorescence intensity.** (a) Whole-brain image. In Figs. 2 and 3, the cortex and hippocampus (boxed regions) were shown, respectively. (b) Regions for quantitative measurement of immunofluorescence intensity. To obtain the relative index of Aβ or 8-oxoG, immunofluorescence intensity levels in the regions enclosed by dotted lines were measured in each digital image using ImageJ 1.49v software (National Institutes of Health, Bethesda, MD, USA). Immunofluorescence intensity in cortical layers II–IV and in the dentate gyrus (DG) of the hippocampus was measured and plotted.

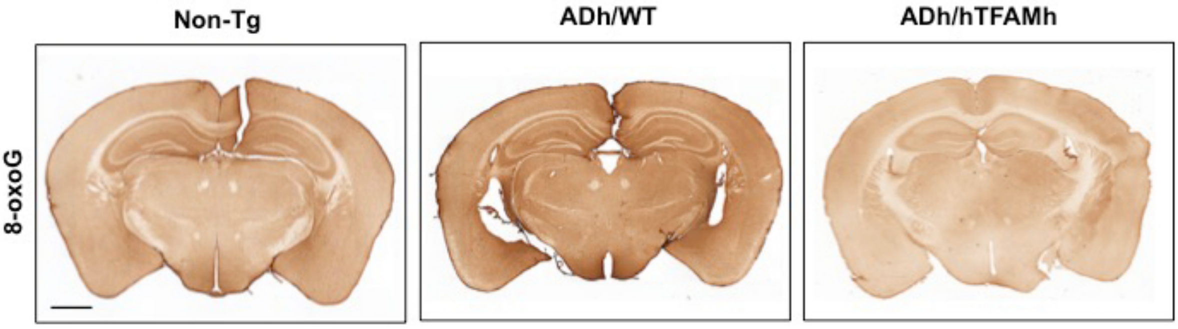

Supplementary Figure S4. Expression of hTFAM suppressed the increase in 8-oxoG levels in mitochondrial DNA of 14-month-old ADh/WT mice. Frozen tissue blocks were cut on a microtome (40 μm thick) and collected as free-floating sections in phosphate-buffered saline (PBS). Sections pretreated with 5 mg/mL RNase were subjected to immunohistochemical analysis with the anti-8-oxo-dG antibody and processed using a Vector ABC kit with the appropriate secondary antibody. The DAB reaction was used to visualize the bound secondary antibody. Virtual slice images of whole brain were acquired using the Nikon Eclipse 80i microscope (Nikon, Co., Tokyo, Japan) equipped with a Stereo Investigator 10 Virtual Slice Module (MBF Bioscience Japan, Chiba, Japan). Scale bar, 1 mm.

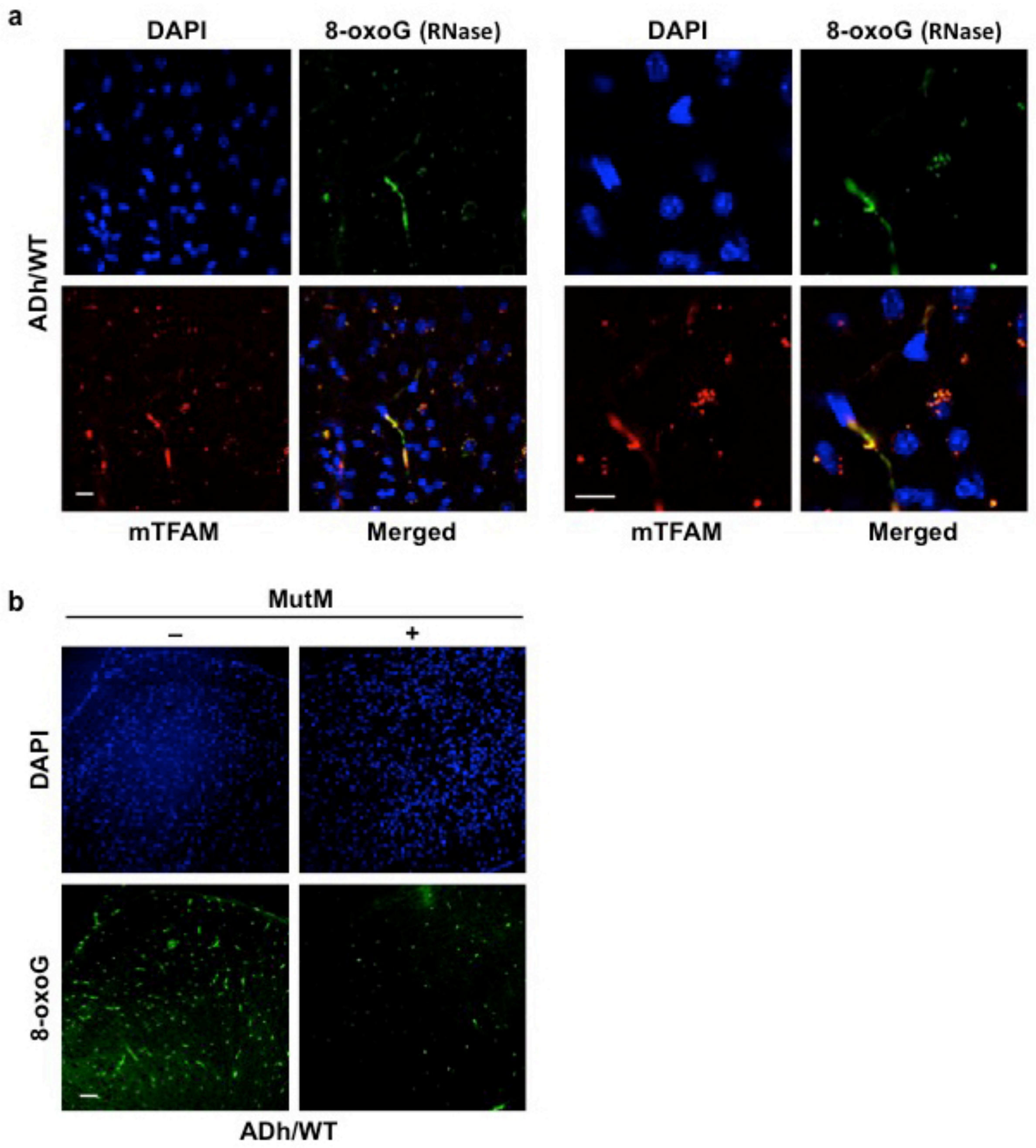

Supplementary Figure S5. Specificity of 8-oxoG immunoreactivity in mitochondrial DNA. (a) Mitochondrial localization of 8-oxoG immunoreactivity in cortical section from 14-month-old ADh/WT mice. Immunofluorescent signals for 8-oxoG are colocalized with those for mouse TFAM (mTFAM), which is a mitochondrial protein. (b) 8-OxoG immunoreactivities in the cortex were diminished by pretreatment with the MutM 8-oxoG DNA glycosylase. Cortical sections from 14-month-old ADh/WT mice pretreated with 5 mg/mL RNase were incubated with 10 μg/mL of MutM protein (F3174, Sigma-Aldrich) in nicking buffer (10 mM Tris-HCl [pH 7.5], 5 mM $ZnCl_2$, 0.5 mM DTT, 0.5 mM EDTA, 1.5% [v/v] glycerol, 100 μg/mL BSA) for 1 h at 37°C. Sections were then subjected to immunofluorescence microscopy with anti-8-oxo-dG. Nuclei were stained with DAPI. MutM treatment significantly decreases the intensity of 8-oxoG immunoreactivity in the cortex of ADh/WT mice. Scale bar, 20 μm.

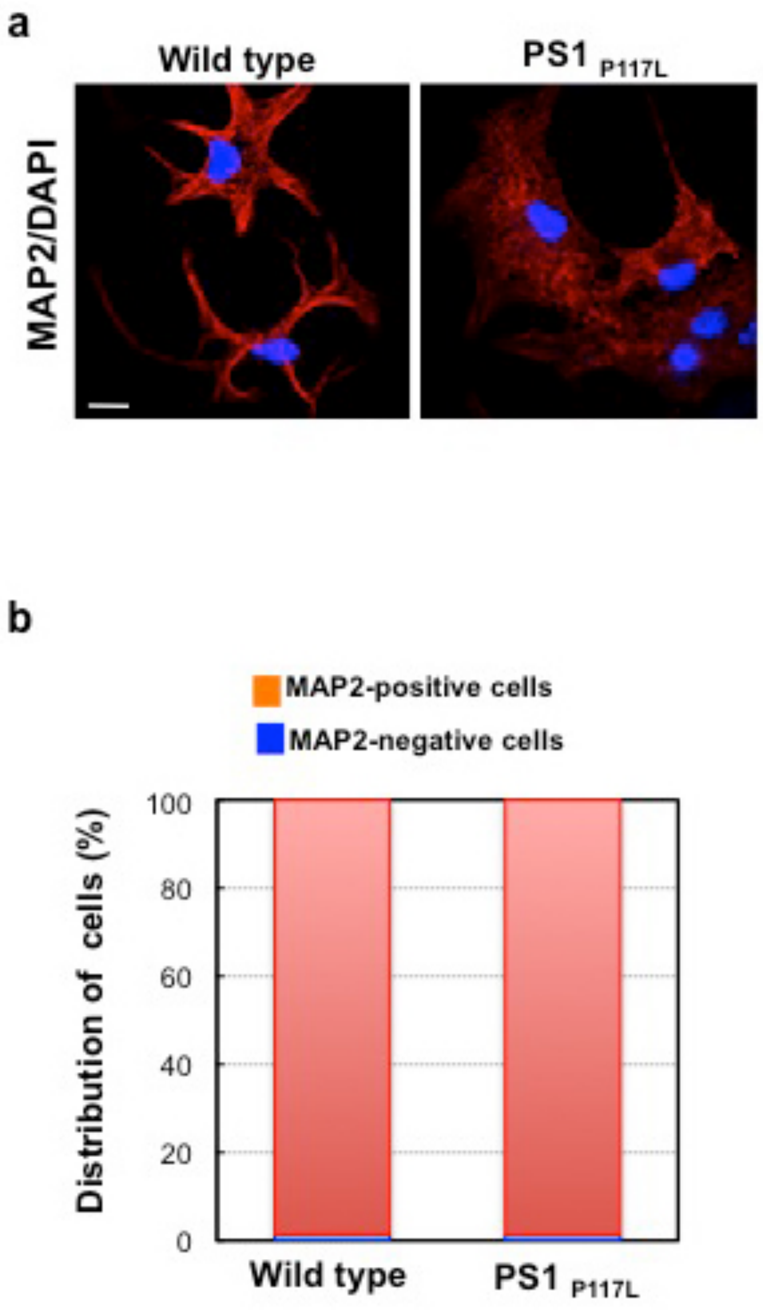

Supplementary Figure S6. A human cell culture model of AD using neurons derived from iPSCs. (a) Immunofluorescence microscopy with anti-MAP2 indicates neuronal phenotype of the cells derived from human iPSCs. Nuclei were stained with DAPI. Scale bar, 20 μm. (b) Quantitative assessment of neuronal population. More than 75 cells in each culture condition were examined for MAP2 immunoreactivity to confirm neuronal differentiation.

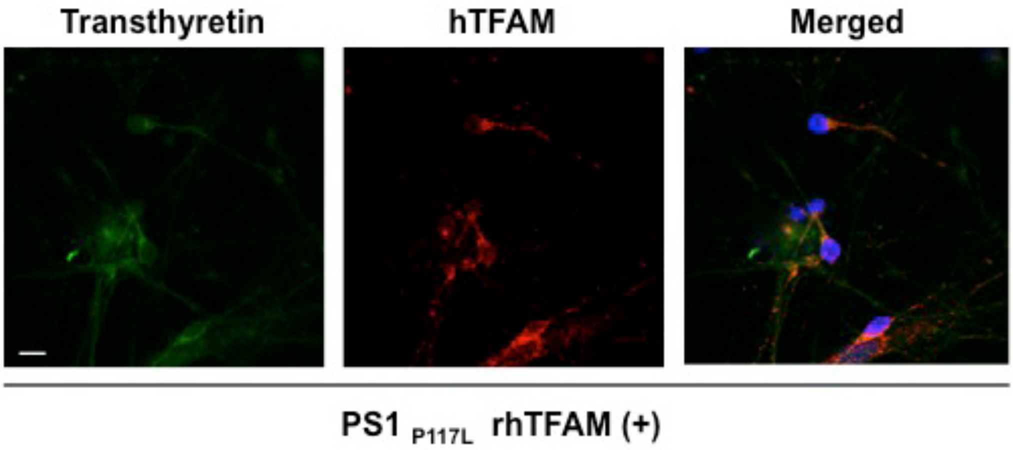

Supplementary Figure S7. Cytoplasmic transthyretin is co-localized with hTFAM. Neurons derived from $PS1_{P117L}$ iPSCs were treated with rhTFAM, then examined by immunofluorescence microscopy with anti-transthyretin and anti-hTFAM. Nuclei were stained with DAPI (blue). Scale bar, 20 μm.

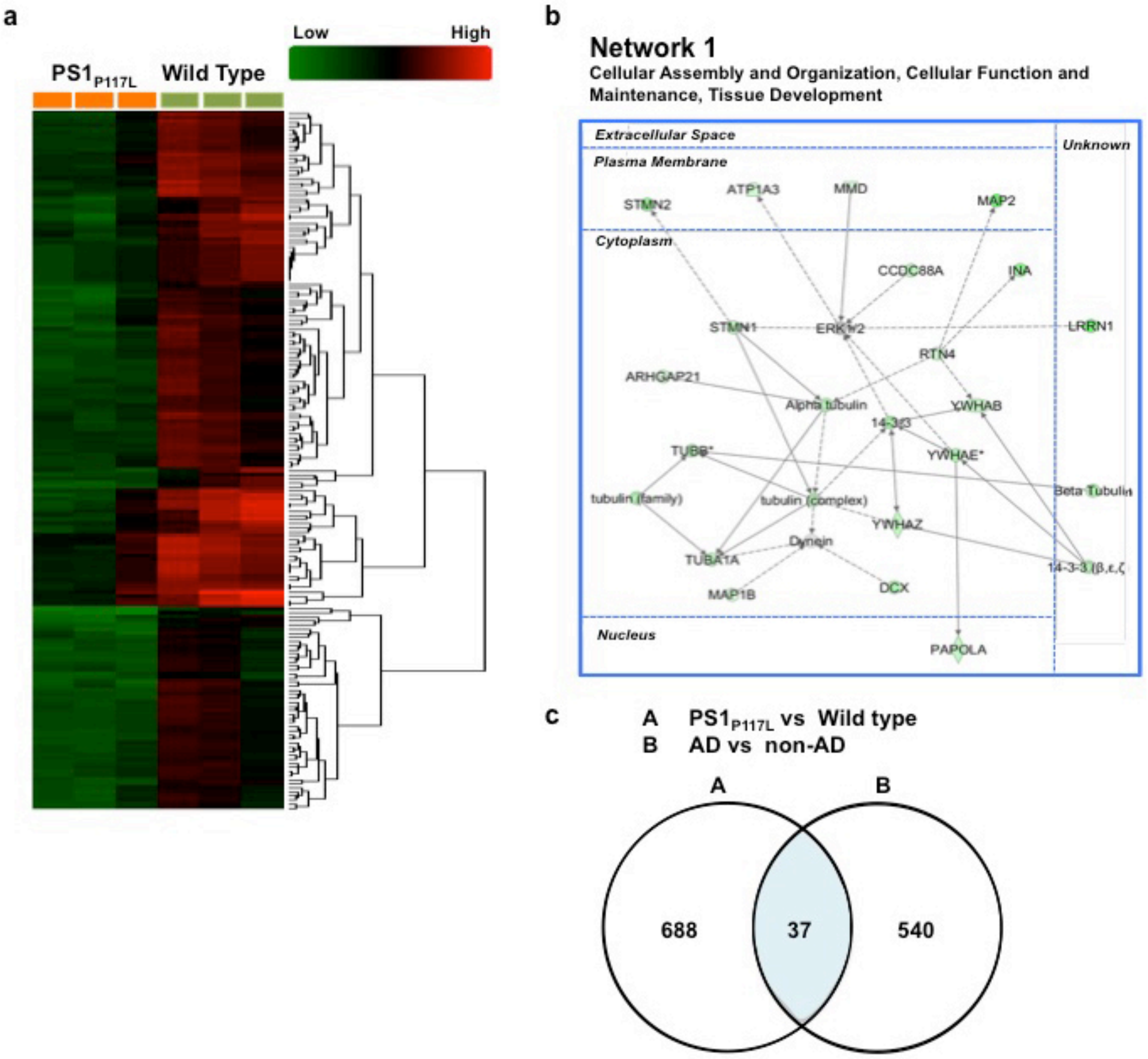

Supplementary Figure S8. **Significant alteration of gene expression in a human neuron model of AD.** (a) Hierarchical partitioning and clustering of 148 clusters exhibited 12-fold or more decreased expression (average raw expression level [$\log_2$] >6.6 in the wild type), in a human neuron model of AD. Student's t-test: $p < 0.05$, n = 3. (b) Top significant network consisted of 21-downregulated genes among 136 functional/pathway eligible genes in the IPA. (c) Comparison of significantly altered gene expression profiles generated by IPA in a human neuron model of AD and the hippocampus from an AD brain. Group A shows the altered gene profile in $PS1_{P117L}$ compared with wild-type cells. Among the clusters obtained with a fold-change >8 and raw expression level ($\log_2$) >2 in both wild-type and $PS1_{P117L}$ cells, 725 genes were eligible for comparison. Group B shows the altered gene profile in the hippocampus of an AD brain vs. that of a non-AD brain. The AD profile described previously[1] was reanalysed. One-way ANOVA was performed with the transcript clusters altered in the AD hippocampus, and the *p*-value for each comparison was determined by unpaired Student's t-test: $p < 0.05$. Fold-change >1.4, n = 3. Up- or downregulated genes in the hippocampus of the AD hippocampus are listed in Supplementary Tables S7 and S8. Among the clusters obtained with a fold-change >1.4 and raw expression level ($\log_2$) >2 in the AD or non-AD hippocampus, 577 genes were eligible for comparison. The 37 genes in common between Group A and B are shown in Supplementary Table S5.

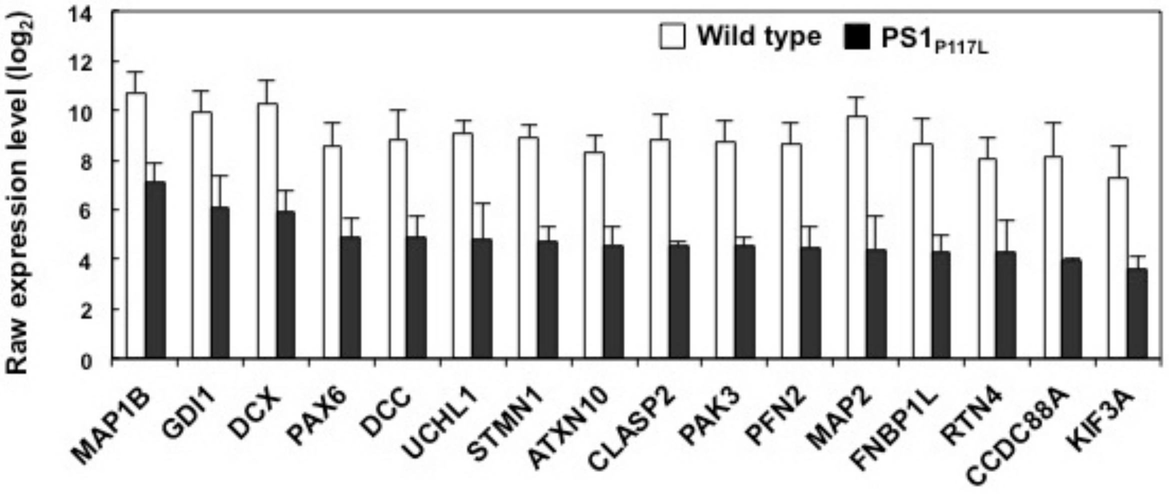

Supplementary Figure S9. Neurons derived from $PS1_{P117L}$ iPSCs exhibited impaired neuritogenesis. Sixteen neuritogenesis-related genes were significantly downregulatedin in neurons derived from $PS1_{P117L}$ iPSCs, compared with those from wild-type iPSCs ($p < 0.05$, Supplementary Table S3).

Supplementary Methods

Genotyping. Alkaline-extracted DNA from tail tissue was subjected to genomic PCR using MightyAmp DNA Polymerase (version 2) according to the manufacturer's instructions (Takara Bio Inc., Shiga, Japan). For genotyping 3xTg-AD mice, the *Psen1*$_{M146V}$ knock-in mutation was detected as previously described[2], and *APP*$_{Swe}$ and *MAPT*$_{P301L}$ transgenes were identified using the following specific primer sets: *APP*$_{Swe}$, 5′-GCTTGCACCAGTTCTGGATGG-3′, 5′-GAGGTATTCAGTCATGTGCT-3′; *MAPT*$_{P301L,}$ 5′-GAGGTATTCAGTCATGTGCT-3′, 5′-TTCAAAGTTCACCTGATAGT-3′. For detecting the hTFAM transgene, the specific primer set was 5′-GAAAGGATCTTCTACGTCGCACAA-3′ and 5′-CCAGCATATGGCATTGTTGCCAA-3′. To determine the copy number of transgenes *APP*$_{Swe}$ and *hTFAM*, we performed quantitative genomic PCR. The primers for quantitative genomic PCR were as follows: *APP*$_{Swe}$ (forward primer, 5′-ATTCAGATCCATCAGGGACCAA-3′; reverse primer, 5′-GCTTGCACCAGTTCTGGATGGT-3′); *hTFAM* (forward primer, 5′-GAAAGGATCTTCTACGTCGCACAA-3′; reverse primer, 5′-CCAGCATATGGCATATGTTGCCAA-3′). As a control, the primers for *RNase P* were used (forward primer, 5′-GCCGGAGCTTGGAACAGA-3′; reverse primer, 5′-GGTGCCTCACCTCAGCCAT-3′). Quantification was conducted with a Thermal Cycler Dice Real-Time System Single (Takara). Each reaction was performed with 25 ng of DNA, 200 nM primers, and 12.5 μL of 2× SYBR green ready reaction mix with Rox (Applied Biosystems, Foster City, CA, USA) in a total volume of 25 μL.

Antibodies. Anti-human Aβ (N) (82E1) mouse IgG monoclonal antibody (1:100), which recognizes the N-terminus of processed Aβ, was obtained from Immuno-Biological Laboratories Co., Ltd. (IBL, Gunma, Japan). The mouse monoclonal anti-8-oxo-dG antibody (N45.1) was obtained from the Japan Institute for the Control of Aging, Shizuoka, Japan. Rabbit polyclonal anti-neuron-specific nuclear protein (NeuN) (ABN78) and rabbit polyclonal anti-MAP2 (AB5622, 1:1,000) were obtained from Millipore (Billerica, MA, USA). Rabbit polyclonal anti-human TFAM (10 μg/mL) has been described27. Goat polyclonal anti-mouse TFAM as a mitochondrial marker (sc-23588) and mouse monoclonal anti-transthyretin (sc-377517) were obtained from Santa Cruz Biotechnology Inc. (Santa Cruz, CA, USA). Anti-mouse TFAM was used for western blot analysis (1:500) and immunostaining (1:500). Anti-transthyretin was used for western blot analysis (1:500) and immunostaining (1:50). Rabbit polyclonal anti-COX IV (4844S, 1:100) was obtained from Cell Signaling Technology Japan (Tokyo, Japan). Rabbit polyclonal anti-single-strand DNA (#18731) was purchased from IBL, and mouse polyclonal anti-VDAC (ab61273, 1:100) was from Abcam (Tokyo, Japan). Mouse anti-β-Actin antibody was obtained from Sigma-Aldrich (Tokyo, Japan). Antibodies against PCNA (sc-56, 1:2000, Santa Cruz) and HSP60 (LK-1, 1:1000, StressGen) were used for western blot analysis using a human neuron model of AD.

Immunoprecipitation. Cells from a human neuron model of AD (2.8 × 10^5 cells) were incubated in maturation medium (ReproNeuro Ach-AD: RCESDA105 kit) containing 100 nM rhTFAM protein for 24 h, and collected using a cell scraper after washing twice with PBS. Cells were fractionated to nuclear and mitochondrial fractions using a Mitochondria Isolation kit (Abcam). Mitochondrial pellets were resuspended in 50 μL of 2× SDS sample buffer and sonicated. The homogenates were diluted in 1× SDS sample buffer with 2-mercaptoethanol and bromophenol blue, and 10 μL of the homogenate was subjected to western blotting after boiling. Immunoprecipitation was performed using the reagents in the EZ ChIP kit (Millipore, Billerica, MA, USA) according to the manufacturer's protocol with some modifications. Briefly, mitochondrial pellets were resuspended in 1 mL of SDS lysis buffer containing 1× Protease Inhibitor Cocktail II (#20-283, Millipore, Billerica, MA, USA) and sonicated. The lysate was incubated for 1 h at 4°C with 60 μL of a 50% suspension of protein G agarose (#16-201, Millipore) and centrifuged. The supernatant was incubated with 0.5 μg of anti-human TFAM or normal mouse IgG (#12-371B, Millipore) for 24 h at 4°C with gentle shaking, and then 60 μL of a 50% suspension of protein G agarose was added and incubated for 1 h at 4°C. After centrifugation at 3,000 × *g* for 1 min, the proteins bound to protein G agarose were washed four times with lysis buffer and subjected to western blotting with anti-transthyretin.

Tissue processing. Animals deeply anesthetized with pentobarbital (75 mg/kg, intraperitoneal administration) were perfused intracardially with saline followed by cold 4% (w/v) paraformaldehyde (PFA) in 0.1 M phosphate-buffered saline (PBS). The brains were removed, immersed for 12 h in the same 4% (w/v) PFA fixative, and then in 20% (w/v) PFA, followed by 30% (w/v) sucrose in PBS at 4°C until the tissue sank. The brains were stored as paraffin-embedded blocks or frozen tissue blocks at −80°C.

Immunohistochemistry. Tissue sections (4 μm thick) were cut from paraffin-embedded blocks on a microtome and mounted from warm water (42°C) onto slides. Sections were allowed to dry overnight at room temperature, deparaffinized in xylene, and rehydrated through a graded ethanol series. Antigen retrieval was performed by boiling sections in plastic Coplin jars containing sodium citrate buffer (10 mM citric acid, 0.05% [v/v] Tween 20, pH 6.0) using a water bath (100°C) for 10 min, followed by cooling for 30 min to room temperature. Sections were blocked with a solution containing 1× Block Ace (Dainippon Pharmaceutical, Osaka, Japan) for 30 min at room temperature, incubated with primary antibodies in 10% (v/v) Block Ace at 4°C overnight, and then incubated with an Alexa Fluor-labeled secondary antibody (Invitrogen, Tokyo, Japan) in a solution containing DAPI (0.05 μg/mL, Sigma-Aldrich) for 45 min at room temperature. Confocal images were acquired using an LSM 510 META or LSM 700 Confocal Microscope system (Carl Zeiss MicroImaging, Tokyo, Japan). The intensity of immunofluorescence was measured in each digital image using ImageJ 1.49v (National Institutes of Health, NIH, Bethesda, MD, USA).

Supplementary References

1. Hokama, M. *et al.* Altered expression of diabetes-related genes in Alzheimer's disease brains: the Hisayama study. *Cereb Cortex* **24**, 2476-2488, doi:10.1093/cercor/bht101 (2014).

2. Guo, Q. et al. Increased vulnerability of hippocampal neurons to excitotoxic necrosis in presenilin-1 mutant knock-in mice. Nat Med **5**, 101-106, doi:10.1038/4789 (1999).
